# Direct formation and site-selective elaboration of methionine sulfoximine in polypeptides†

**DOI:** 10.1039/d2sc04220g

**Published:** 2022-11-14

**Authors:** Yuxuan Ding, Simon S. Pedersen, Alex Lin, Ruoyu Qian, Zachary T. Ball

**Affiliations:** Department of Chemistry, Rice University Houston Texas 77005 USA zb1@rice.edu; Carbon Dioxide Activation Center (CADIAC), Interdisciplinary Nanoscience Center, Department of Chemistry, Aarhus University Gustav Wieds Vej 14 8000 Aarhus C Denmark

## Abstract

Sulfoximines are emerging moieties for medicinal and biological chemistry, due in part to their efficacy in selective inhibition of amide-forming enzymes such as γ-glutamylcysteine synthetase. While small-molecule sulfoximines such as methionine sulfoximine (MSO) and its derivatives are well studied, structures with methionine sulfoximine residues within complex polypeptides have been generally inaccessible. This paper describes a straightforward means of late-stage one-step oxidation of methionine residues within polypeptides to afford NH-sulfoximines. We also present chemoselective subsequent elaboration, most notably by copper(ii)-mediated N–H cross-coupling at methionine sulfoximine residues with arylboronic acid reagents. This development serves as a strategy to incorporate diverse sulfoximine structures within natural polypeptides, and also identifies the methionine sulfoximine residue as a new site for bioorthogonal, chemoselective bioconjugation.

## Abstract 2

Sulfoximines are emerging moieties for medicinal and biological chemistry. This work describes the late-stage incorporation of methionine sulfoximine residues into polypeptides and chemoselective subsequent elaboration of NH-sulfoximines.

## Graphical abstract

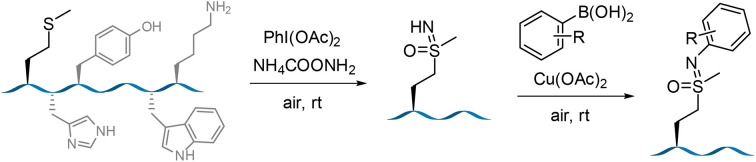

## Introduction

Sulfoximine compounds have recently received significant interest as bioactive agents.^1,2^ The sulfoximine group is often considered a tetrahedral transition-state mimetic in enzyme inhibition, and synthetic methods targeting small-molecule sulfoximines have been developed, especially in recent years.^3^ The first discovered sulfoximine was the amino acid derivative, methionine sulfoximine (MSO), which mimics glutamine and inhibits the enzymes γ-glutamylcysteine synthetase and glutamine synthetase.^4,5^ It was soon discovered that *N*-alkyl derivatives of the parent methionine sulfoximine, such as buthionine sulfoximine, have altered and potentially improved biological properties.^6,7^

Despite the unique chemical properties and biological activity of MSO and its N–H derivatives, the synthesis and reactivity of sulfoximines within methionine residues in larger polypeptides are largely unknown, owing to the synthetic challenges of polyfunctional macromolecules. While recent synthetic methods for sulfoximidation of thioether starting materials have significantly improved upon earlier harsh 2-step oxidation and imidation processes,^8,9^ only a few simple protected dipeptides substrates have been demonstrated.^10^ Similarly, elaboration of N–H sulfoximines,^3^ including by cross coupling reactions,^11–16^ has been studied in small molecule contexts. We are not aware of prior examples of sulfoximine synthesis or subsequent N–H elaboration reactions within larger peptides. In this article, we report selective methionine sulfoximidation within unprotected polypeptides, and demonstrate selective sulfoximine N–H modification *via* a Chan–Lam-type oxidative cross coupling^17,18^ with boronic acid reagents.

The method also serves as a two-step approach to bioconjugation at methionine. Methionine represents an attractive bioconjugation target, with unique chemical properties that complement the traditional nucleophilic bioconjugation targets of cysteine thiol and lysine amino groups.^19^ In spite of this, only in recent years have limited examples of methionine-selective bioconjugation approaches appeared. Although limited by weak nucleophilicity, the thioether group is readily oxidizable, and several groups have taken advantage of this to develop redox-based bioconjugation. Strained oxaziridine reagents selective for methionine imidation have been developed,^20^ and a hypervalent iodonium salt allows bioconjugation *via* an α-diazo sulfonium cation.^21^ In addition, photoredox alkylation selective for the methyl moiety in methionine residues has been demonstrated.^22^

## Results and discussion

**Fig. 1:**
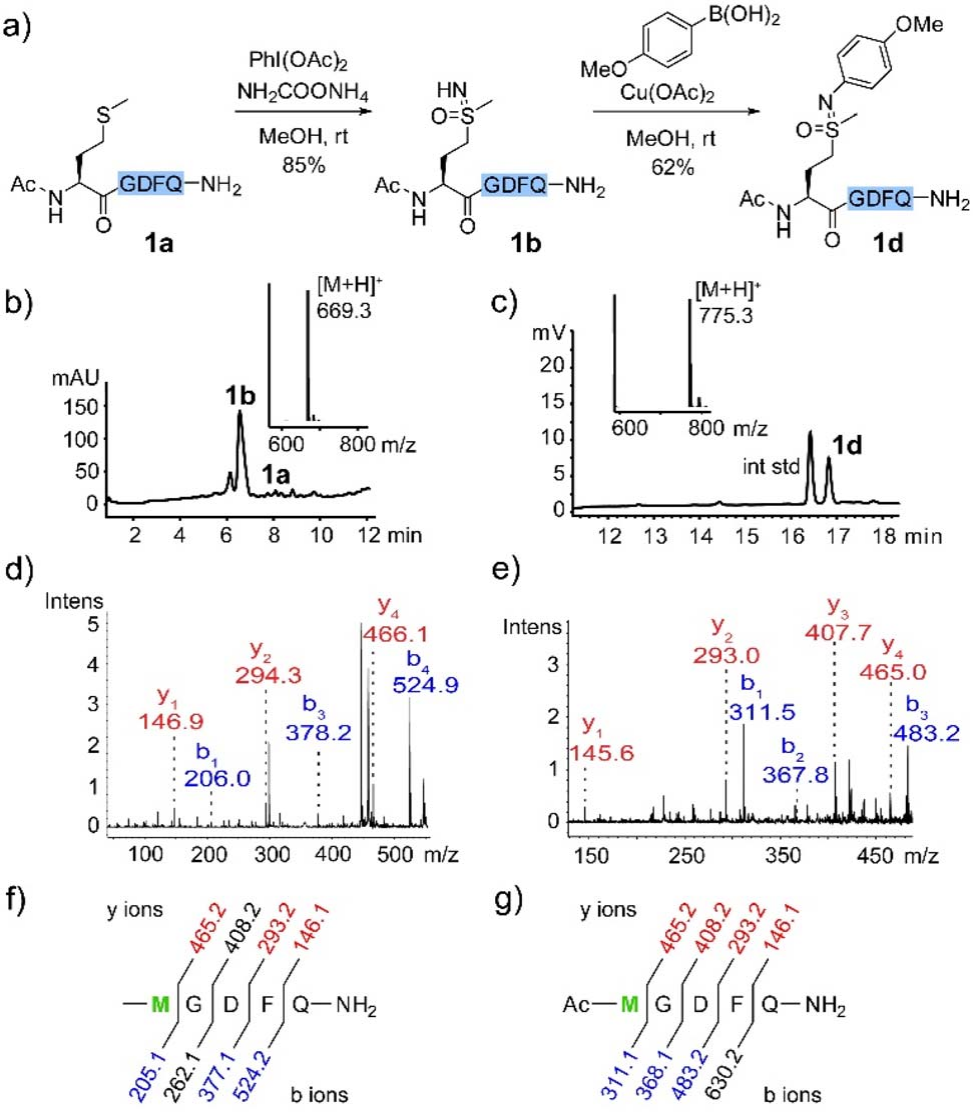
(a) Methionine sulfoximidation and N–H cross-coupling. Conditions: 1a (0.01 mmol), PhI(OAc)_2_ (0.025 mmol), and NH_2_COONH_4_ (0.2 mmol) in MeOH (1 mL); 1b (0.2 mM), 4-methoxylphenylboronic acid (2 mM), and Cu(OAc)_2_ (1 mM) in MeOH. (b) Crude HPLC trace and ESI-MS spectrum of oxidation reaction. (c) Crude HPLC trace and ESI-MS spectrum of coupling reaction (with an internal standard). (d) MALDI-MS/MS spectrum of 1b. (e) MALDI-MS/MS spectrum of 1d. (f) Sequence and fragmentation ladder of 1b. (g) Sequence and fragmentation ladder of 1d.

We first examined selective methionine sulfoximidation using a methionine-containing polypeptide 1a as model. Oxidation of thioethers with (diacetoxyiodo)benzene in the presence of an ammonium source was recently demonstrated to provide direct access to sulfoximine compounds in two simple protected dipeptides.^10^ We set out to explore whether a method based on this reagent might be useful for more diverse and complex polypeptide substrates. To our delight, after a brief optimization of oxidation conditions (Table S1†), we determined 2.5 equivalents of PhI(OAc)_2_ and 20 equivalents of NH_2_COONH_4_ provided the expected product 1b in 85% yield (Fig. 1a and b). MALDI-MS/MS fragmentation established methionine as the modified site (Fig. 1d and f).

Our lab has recently reported a variety of selective bioconjugation approaches based on Chan–Lam coupling of boronic acid reagents with peptide or protein X–H bonds.^23–27^ In a further orienting experiment, isolated sulfoximine peptide 1b proved to be a remarkably reactive substrate for Chan–Lam coupling mediated by Cu(OAc)_2_ in methanol solvent (Fig. 1a and c). Our efforts in this area were encouraged by a few reports of copper-catalyzed coupling with simple sulfoximine substrates.^28,29^ The structure of 1d was confirmed by MALDI-MS/MS (Fig. 1e and g) and ^1^H NMR of the isolated product (see ESI†). No evidence of coupling at any other sites, such as the aspartate carboxylate or any amide groups, was observed under these conditions.

With an initial proof of concept in hand, we examined the peptide scope of the methionine sulfoximidation reaction (Table 1). For peptides (2a, 7a, and 8a) with poor solubility in MeOH, 20% water was added as a co-solvent, although yields overall remain more modest for poorly-soluble substrates. In general, methionine sulfoximine residues were introduced to a range of unprotected polypeptides, including the natural methionine-containing peptides, substance P (7a) and α-MSH (α-melanocyte-stimulating hormone) (8a). This reaction tolerates a wide variety of amino acids, including adjacent bulky residues (leucine), charged residues (lysine, arginine, aspartate, and glutamate). Even the readily oxidizable residues tyrosine and tryptophan are tolerated under these conditions,^30,31^ although some byproduct oxidation was observed at prolonged reaction times and higher oxidant equivalents. Oxidation of cysteine was observed under the reaction conditions, and that residue was thus not explored further. HPLC analysis shows nearly full conversion in most cases. Including water as a co-solvent does seem to decrease reaction rates somewhat, and the modest yields observed in a few cases (*i.e.*2b, 7b) are the result of incomplete conversion.

**Table 1:** Peptide scope of methionine sulfoximidation^a^

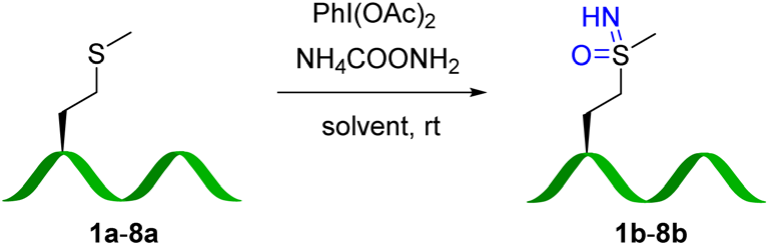		
Entry	Peptide	Yield^b^ (%)
1	Ac-**M**GDFQ-NH_2_	85
2^c^	Ac-**M**GKFQ-NH_2_	50
3	Ac-YG**M**LNP-NH_2_	92
4	Ac-VG**M**SWP-NH_2_	86
5	Ac-FPQSG**M**-NH_2_	84
6	Ac-MGRFTINP-NH_2_	67
7^c^	Ac-RPKPQQFFGL**M**-NH_2_ (*substance P*)	65
8^c^	Ac-SYS**M**EHFRWGKPV-NH_2_ (*α-MSH*)	76

a Conditions: peptides (1a–8a) (0.01 mmol), PhI(OAc)_2_ (0.025 mmol), and NH_2_COONH_4_ (0.2 mmol) in MeOH or MeOH : H_2_O 4 : 1 (1 mL) at room temperature for 16 h.

b Yield calculated by RP-HPLC.

c Reactions were performed in MeOH with 20% H_2_O as co-solvent.

**Fig. 2:**
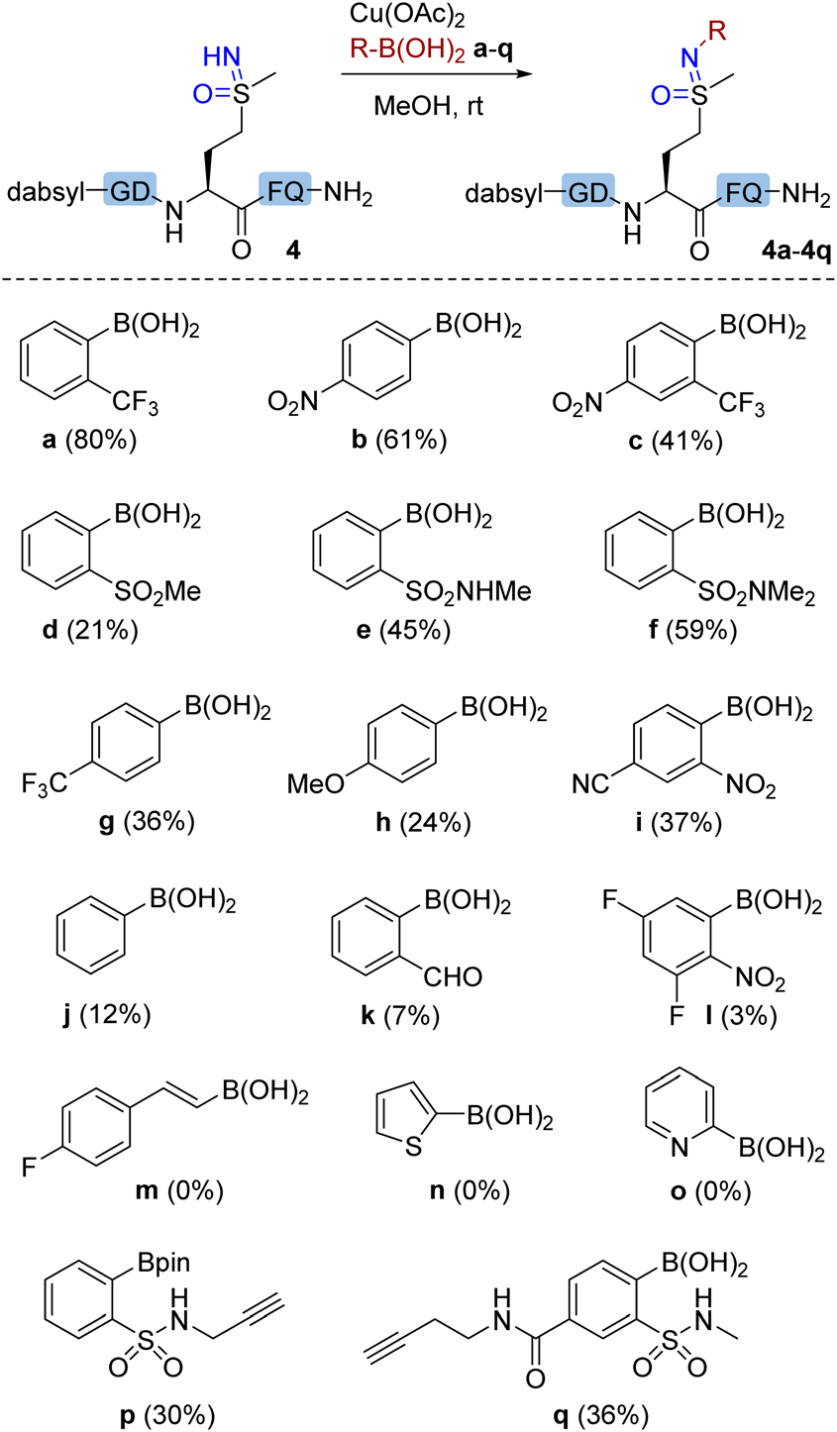
Scope of the boronic acid reagents. Conditions: dabsyl-labeled methionine sulfoximine-containing peptide (4) (0.2 mM), boronic acid a–q (4 mM), and Cu(OAc)_2_ (1 mM) in MeOH at room temperature for 16 h. Yields were determined by RP-HPLC using dabsyl glycine as an internal standard.

Next, we explored the scope of arylboronic acid reagents for copper(ii)-mediated N–H coupling of methionine sulfoximine-containing peptides. Using 1b as a model and after screening a series of solvents and metal salts (Tables S2 and S3†), MeOH and copper(ii) acetate were determined as the optimal choice and were used in subsequent studies. Optimization studies indicated that 20% aqueous conditions were similarly effective, but decreased reaction yields were seen as the amount of water was increased. A dabsyl-labeled methionine sulfoximine-containing peptide 4 was prepared and used as substrate to enable facile HPLC-based analysis of reaction efficiency (Fig. 2). A variety of arylboronic acids bearing electron-withdrawing *ortho* (a, c, d, e, f, and i) or *para* (b and g) substituents gave successful N–H coupling products. Arylation products were even observed with simple phenylboronic acid (j) and 4-methoxylphenyl boronic acid (h), in stark contrast to our previous studies of arylation at cysteine S–H,^25^ backbone amide N–H,^23^ or N-terminal amine sites.^26^ However, no product was observed with (*E*)-alkenyl boronic acid (m) or heteroaryl boronic acids (n and o). The successful coupling of alkyne-containing boronic acids (p and q) demonstrated the selectivity of Chan–Lam reactivity over well-known copper-mediated alkynyl C–H activation pathways and demonstrates the possibility of using sulfoximine elaboration to enable subsequent additional bioorthogonal elaboration.

For quantification purposes (Fig. S8†), 4-nitrophenyl boronic acid (b) was employed as the coupling reagent to test the peptide scope (Table 2), which allowed facile yield determination by HPLC analysis. Most sulfoximine peptides were converted to corresponding arylated products, although yields and reaction efficiency vary somewhat. Peptide 3b resulted in the lowest yield of the peptides 1b–8b. A structural basis for cross-coupling efficiency is difficult to ascertain, although steric bulk of a proximal leucine residue is one possible explanation. To our delight, this reaction showed good amino acid tolerance. HPLC and MS/MS analysis (Fig S73 and S75,†7c and 8c) identified the methionine sulfoximine residue as the only modification site.

**Table 2:** Peptide scope of N–H cross-coupling^a^

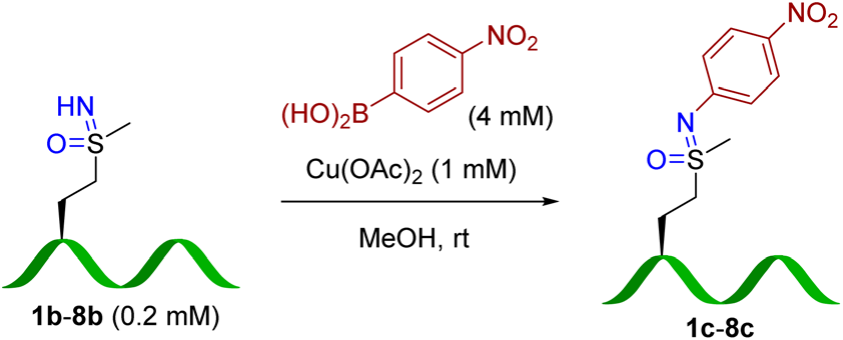		
Entry	Peptide (sulfoximine)	Yield^b^ (%)
1	Ac-**M**GDFQ-NH_2_	94
2	Ac-**M**GKFQ-NH_2_	61
3	Ac-YG**M**LNP-NH_2_	40
4	Ac-VG**M**SWP-NH_2_	75
5	Ac-FPQSG**M**-NH_2_	50
6	Ac-**M**GRFTINP-NH_2_	76
7	Ac-RPKPQQFFGL**M**-NH_2_ (*substance P*)	50
8	Ac-SYS**M**EHFRWGKPV-NH_2_ (*α-MSH*)	51

a Conditions: methionine sulfoximine-containing peptides (1b–8b) (0.2 mM), 4-nitrophenylboronic acid (4 mM), and Cu(OAc)_2_ (1 mM) in MeOH at room temperature for 16 h.

b Yield calculated by RP-HPLC.

**Fig. 3:**
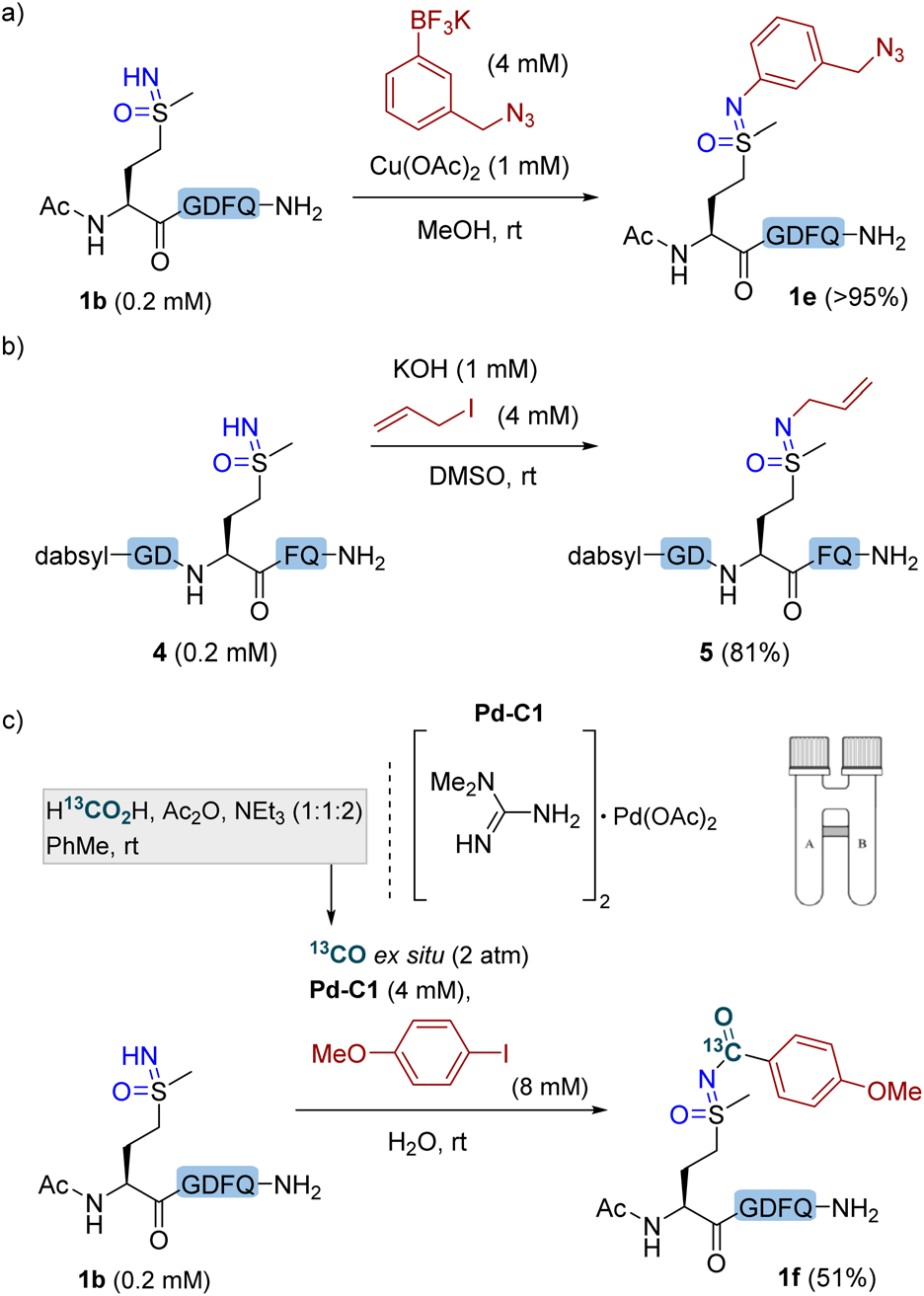
Site-selective elaboration of methionine sulfoximine in polypeptides (a) N–H cross-coupling with potassium trifluoroborate. Conditions: peptide 1b (0.2 mM), potassium 3-(azidomethyl)phenyltrifluoroborate (4 mM), and Cu(OAc)_2_ (1 mM) in MeOH at rt for 16 h. (b) NH-alkylation. Conditions: peptide 4 (0.2 mM), allyl iodide (4 mM), and KOH (1 mM) in DMSO at room temperature for 16 h. (c) Carbonylative coupling and ^13^C labeling. Conditions: peptide 1b (0.2 mM), Pd–C1 (4 mM), 4-iodoanisole (8 mM) and ^13^CO (2 atm) generated *ex situ* within a two-chamber system (see ESI†) in H_2_O at rt for 16 h. Yields were determined by RP-HPLC using internal standards.

Other structurally and chemically interesting site-selective modifications of methionine sulfoximine residues within polypeptides seem to be possible. An azide handle was readily incorporated to peptide 1b with a functionalized potassium trifluoroborate reagent (Fig. 3a). The reaction of sulfoximine peptide 4 with allyl iodide and potassium hydroxide resulted in NH-alkylation product 5 in 81% yield (Fig. 3b).^32^ In addition, inspired by the palladium(ii)-catalyzed carbonylation of NH-sulfoximines with aryl iodides,^14,33,34^ we achieved the carbonylation of methionine sulfoximine in polypeptide 1b by using a water-soluble Pd complex Pd–C1 as the catalyst under a CO atmosphere. In this case, CO was conveniently and safely generated within a two-chamber system,^35^ with aprotic organic conditions in one chamber allowing the reaction of formic acid, acetic anhydride and triethylamine in toluene to release CO,^36^ and an aqueous-phase carbonylative coupling in the other chamber. The aqueous conditions of this carbonylative cross-coupling demonstrate the potential of methionine sulfoximine as a bioorthogonal handle under biologically relevant conditions.

Using ^13^C-labeled formic acid as a ^13^CO source, it was possible to afford functionalized and isotopically labeled product 1f (Fig. 3c). In recent years, carbon isotope labeling has found increasing applications in the pharmacological industry for evaluation of drug candidates' metabolic profile and other important attributes, by means of positron emission tomography (PET) imaging,^37–39^ hyperpolarized magnetic resonance imaging (MRI),^40^ or accelerator mass spectrometry (AMS).^41^ It is noteworthy that the carbonylative coupling affords simultaneous isotope labeling and bioconjugation, yet avoids the need to separately synthesize an isotopically labeled bioconjugation reagent, since the isotope label (^13^CO) and chemical functionalization (aryl iodide) are separate entities in this three-component coupling.

**Fig. 4:**
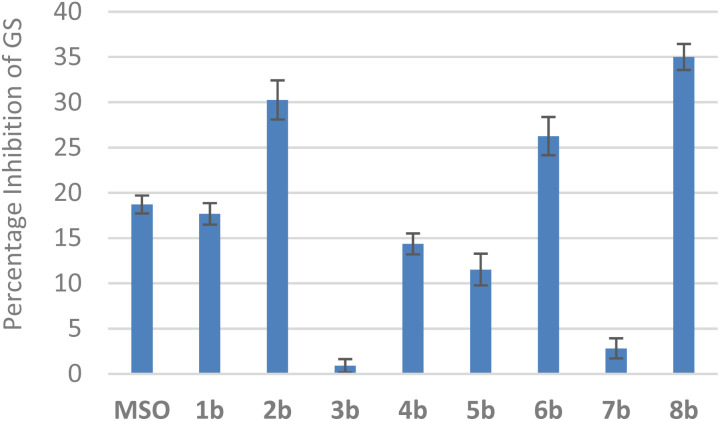
Inhibition of glutamine synthetase (GS) by methionine sulfoximine (MSO) and methionine sulfoximine-containing peptides (1b–8b). Assays were performed at 1 mM inhibitor concentration using a glutamine synthetase activity assay kit (Abcam).

To investigate the potential bioactivity of methionine sulfoximine-containing polypeptides, we screened peptides 1b–8b as potential inhibitors of glutamine synthetase (GS) activity (Fig. 4), and compared them to the amino acid, methionine sulfoximine (MSO), a typical inhibitor of GS activity. Several peptides showed inhibition of glutamine synthetase, and peptides 2b, 6b, and 8b exhibited inhibitory activity higher than that observed for MSO. These preliminary results indicate that incorporating the sulfoximine group within larger peptide structures is a useful strategy to improve inhibitor potency and selectivity.

## Conclusions

In conclusion, we demonstrated the late-stage incorporation of methionine sulfoximine residues into polypeptides, using (diacetoxyiodo)benzene under partially aqueous conditions. Furthermore, initial NH-sulfoximines can be subsequently elaborated into diverse sulfoximine derivatives. Sulfoximines have unique reactivity that allows site-selective copper(ii)-mediated N–H cross-coupling with arylboronic acids. Preliminary reaction screening indicates that sulfoximine structures may be uniquely suited as site-selective bioconjugation sites in a variety of diverse reaction mechanisms. Simple nucleophilic reactivity with an S_N_2 electrophile affords *N*-alkyl derivatives, and palladium-catalyzed carbonylative cross coupling with an aryl iodide affords an *N*-acyl sulfoximine without interference of other peptide side chains. Preliminary bioactivity screens indicate that methionine sulfoximine structures within complex polypeptides can result in useful enzyme inhibitory activity. These studies provide a foundation for the study of sulfoximine structures within polypeptides as bioactive agents or as handles for further chemical elaboration.

## Author contributions

Y. D. and Z. T. B. conceived the ideas, and Y. D., Z. T. B., and S. S. P. designed the experiments. Y. D., S. S. P., A. L., and R. Q. conducted the experiments. Y. D., Z. T. B., and S. S. P. analyzed the data and wrote the initial draft together. All authors reviewed and edited the writing.

## Conflicts of interest

There are no conflicts to declare.

## Supplementary Material

SC-013-D2SC04220G-s001
