# Supplementary material for: Direct formation and site-selective elaboration of methionine sulfoximine in polypeptides
Source: Chem Sci. 2022 Nov 14;13(47):14101–5. doi: 10.1039/d2sc04220g (PMC9728511; doi:10.1039/d2sc04220g)
Supplement: SC-013-D2SC04220G-s001 [file SC-013-D2SC04220G-s001.pdf]

Electronic Supplementary Material (ESI) for Chemical Science.
This journal is © The Royal Society of Chemistry 2022

Direct formation and site-selective elaboration of methionine sulfoximine in polypeptides

Yuxuan Ding,[a] Simon S. Pedersen,[a,b] Alex Lin,[a] Ruoyu Qian[a] and Zachary T. Ball [a]*

[a] Department of Chemistry, Rice University, Houston, Texas 77005, United States

[b] Carbon Dioxide Activation Center (CADIAC), Interdisciplinary Nanoscience Center, Department of Chemistry, Aarhus University, Gustav Wieds Vej 14, 8000 Aarhus C, Denmark

*zb1@rice.edu

Supplementary Information

Table of Contents

Table of Contents S2

General Information S3

HPLC Quantification S4

- **Sample Quantification for Table S1** S4
- **Sample Quantification for Table 1** S8
- **Sample Quantification for Table S2** S9
- **Sample Quantification for Figure 2** S10
- **Sample Quantification for Table 2** S11
- **Quantification for Figure 3** S12

Experimental Procedures S14

Preparation of Reagents S18

Supplementary Data S20

- **Cysteine tolerance test** S20
- **For Table S1** S21
- **For Table S2** S22
- **For Table S3** S23
- **For Table 1** S25
- **For Figure 2** S29
- **For Table 2** S45
- **For Figure 3** S53
- **For Figure 4** S59

Characterization Data S61

- **Synthesized peptide starting materials** S61
- **Methionine sulfoximine peptides** S68
- ***N*-Arylated peptides** S78
- **Small molecule reagents** S85

References S88

General Information

Chemicals

All chemicals were purchased from commercial suppliers and used without further purification. The peptide α-MSH (α-melanocyte-stimulating hormone, **8a**, Ac-SYSMEHFRWGKPV-NH_2) was purchased from MedChemExpress (HY-P0252). The glutamine synthetase activity assay kit was purchased from Abcam (ab284572). The ion-supported $PhI(OAc)_2$ analogue **S1** was prepared by a known protocol.[1] To prepare *N*-methylmorpholine (NMM) buffer, solid NMM (TCI, M0370) was added to MilliQ water, and the pH adjusted by addition of aq HCl (1 M soln). Other buffers were prepared analogously, adjusting pH as necessary with aq HCl (1 M soln) or aq NaOH (1 M soln).

Instrumentation

Reverse-phase HPLC (RP-HPLC) was performed on a Shimadzu Nexera instrument with Phenomenex Jupiter 4µ Proteo 90A (250 mm × 4.6 mm for analytical scale) and Phenomenex Jupiter 4µ Proteo 90A (250 × 15 mm for preparative scale) column. The flow rate was 1 mL/min for analytical scale and 8 mL/min for preparative scale. A gradient of acetonitrile/water with 0.1% trifluoroacetic acid (TFA) was employed. Compounds were detected by UV detector at the wavelength indicated.

ESI-MS was conducted on Agilent Single Quadrupole LC/MS spectrometer with an Agilent ZORBAX RRHD Eclipse Plus C18 column (95A, 2.1 x 50 mm, 1.8 um). The flow rate was 0.4 mL/min. A gradient of acetonitrile/water with 0.1% formic acid was employed.

MALDI-TOF MS and MS/MS were conducted on Bruker Daltonics Autoflex Speed-MALDI-TOF/TOF spectrometer. A sample was mixed with sinapic acid or α-cyano-4-hydroxy-cinnamic acid (CHCA) (20 mg/mL solution in 50:50:0.1 H_2O/MeCN/TFA) on a MALDI plate.

ESI-MS/MS and HRMS were conducted on Agilent Quadrupole-TOF LC/MS spectrometer.

^{1}H and ^{13}C NMR spectra were obtained on a Bruker AVANCE 600 spectrometer.

UV-Vis spectra were obtained on a Cary spectrophotometer. Absorbance at a specific wavelength was measured on a Thermo Scientific NanoDrop 2000c spectrophotometer.

HPLC Quantification

HPLC quantification for the optimization of methionine sulfoximidation (Table S1)

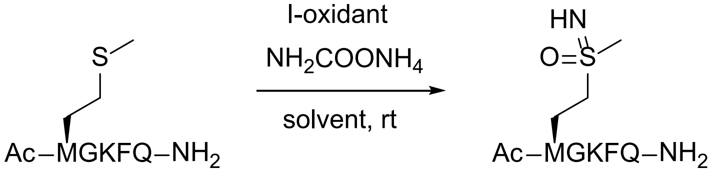

2a **2b**

As shown in Figure S1, iminodimethyl-λ[6]-sulfanone does not have a significant UV absorbance at 220 nm. Thus, HPLC yields were determined assuming that the molar extinction coefficients at 220 nm (ε_{220}) of starting material **2a** and product **2b** are the same. For optimization studies, 4-fluoro-2-nitrobenzaldehyde was used as an internal standard. The molar ratio $\frac{\text{n (SM)}}{\text{n (int std)}}$ and extinction coefficient ratio of starting material to internal standard $\frac{\varepsilon_{200}\ \text{(SM)}}{\varepsilon_{200}\ \text{(int std)}}$ were determined by ^{1}H NMR and RP-HPLC.

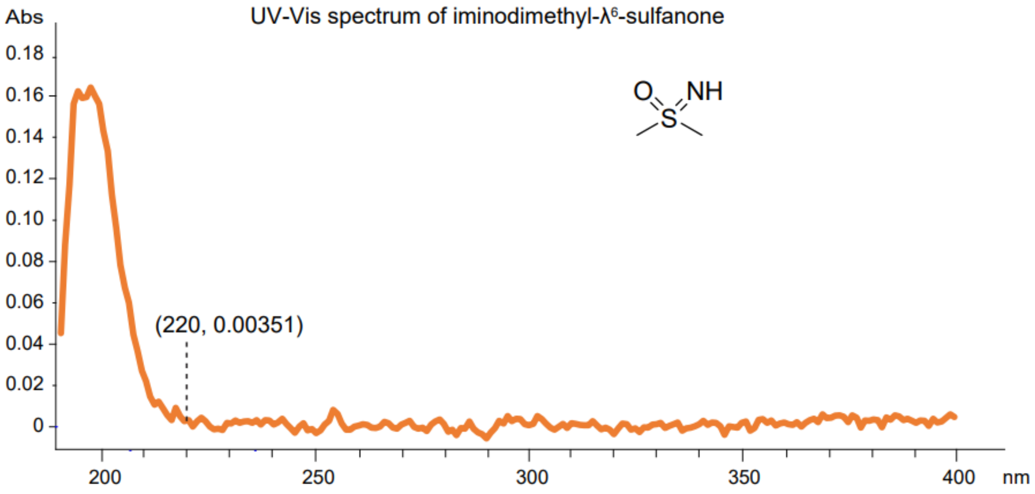

Figure S1. UV-Vis spectrum of iminodimethyl-λ[6]-sulfanone (0.2 mM).

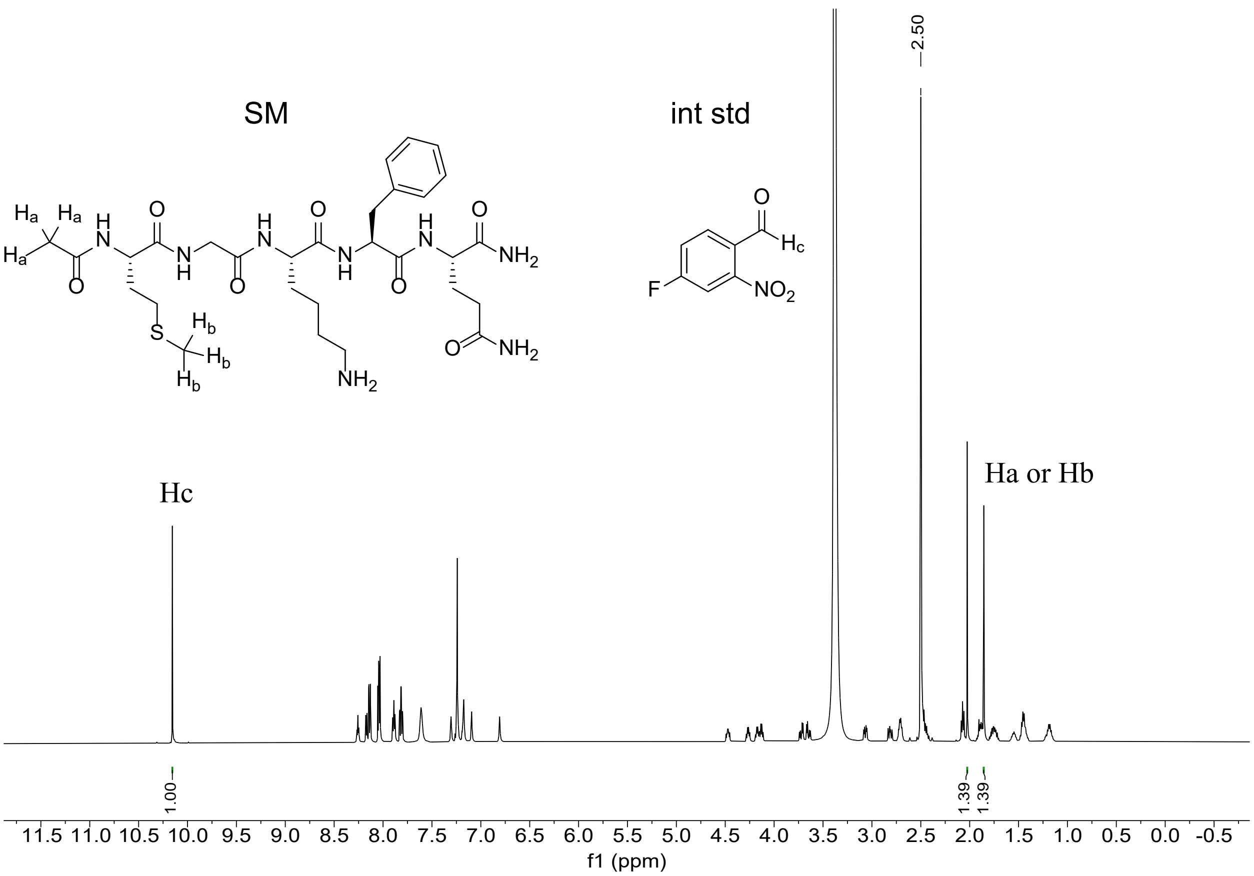

	peak area	proton number	amount	$\frac{\text{n (SM)}}{\text{n (int std)}}$
SM (Ha or Hb)	1.39	3	0.463	0.463
Int std (Hc)	1.00	1	1	

Figure S2. ^{1}H NMR spectrum of a mixture of starting material (**2a**) and internal standard 4-fluoro-2-nitrobenzaldehyde, and calculation of the molar ratio, $\frac{\text{n (SM)}}{\text{n (int std)}}$.

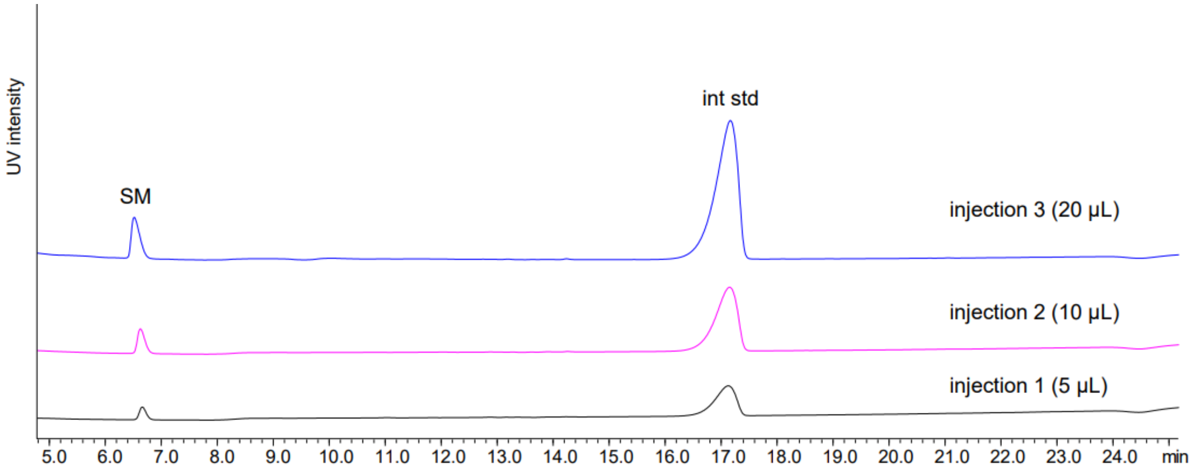

injection volume	peak area (SM)	peak area (int std)	Peak area ratio	amount ratio	extinction coefficient ratio	$\frac{\varepsilon 220\ (\text{SM})}{\varepsilon 220\ (\text{int std})}$ average
5 μL	1829208	16994914	1:9.29	0.463:1	1:4.30	1:4.14
10 μL	3769047	32277221	1:8.56		1:3.97	
20 μL	7247379	64712548	1:8.93		1:4.14	

Figure S3. HPLC traces (5-70% MeCN over 21 min at 220 nm) of the mixture of starting material (**2a**) and internal standard 4-fluoro-2-nitrobenzaldehyde, and calculation of the extinction coefficient ratio $\frac{\varepsilon_{200}\ (\text{SM})}{\varepsilon_{200}\ (\text{int std})}$.

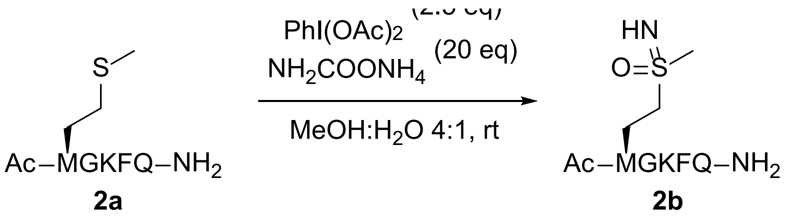

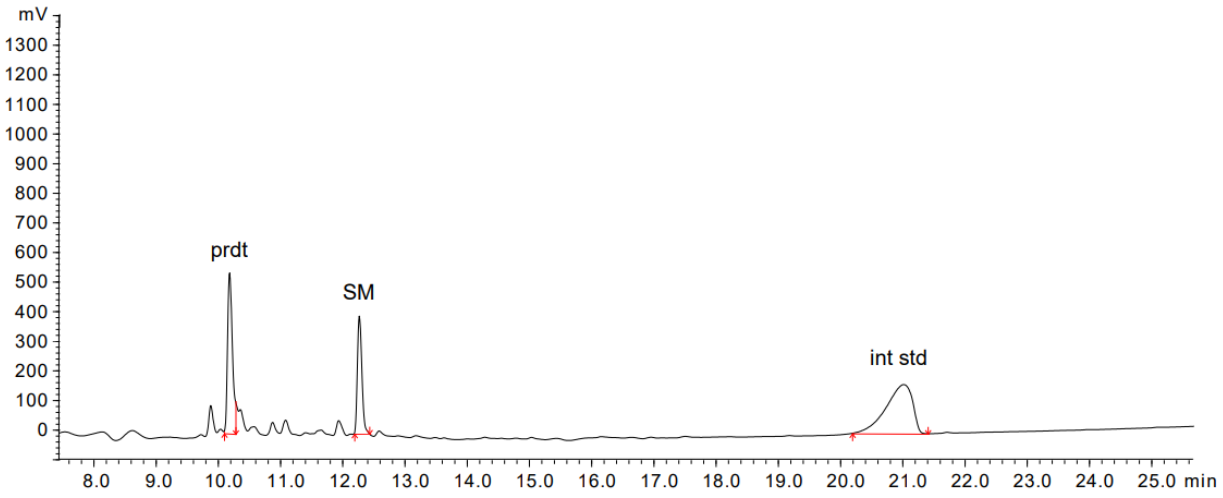

	peak area	yield
product	2948385	50%
int std	4901367	-

$$\text{yield} = \frac{\text{peak area (product)}}{\text{peak area (int std)}} \div \frac{\varepsilon 220\ (\text{SM})}{\varepsilon 220\ (\text{int std})} \div \frac{\text{concentration (SM)}}{\text{concentration (int std)}} \times 100\%$$

$$= \frac{2948385}{4901367} \div \frac{1}{4.14} \div \frac{0.01}{0.002} \times 100\% = 50\%$$

Figure S4. Sample calculation of yield calculated by analytical RP-HPLC (5-70% MeCN over 21 min at 220 nm) with 4-fluoro-2-nitrobenzaldehyde as an internal standard.

HPLC quantification for methionine sulfoximidation using $PhI(OAc)_2$ and NH_2COONH_4 (Table 1)

As mentioned before, iminodimethyl-λ^6-sulfanone does not have a significant UV absorbance at 220 nm (Figure S1). Thus, HPLC yields were determined assuming that the molar extinction coefficients at 220 nm (ε_{220}) of starting material **1a** and product **1b** are the same. A known concentration of **1a** was injected 3 times as a response factor calibration. The yield was then calculated by integration of the peak areas of product in the crude reaction.

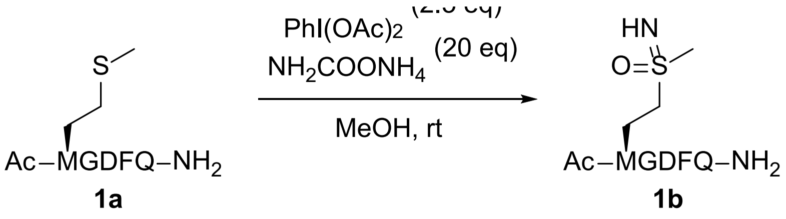

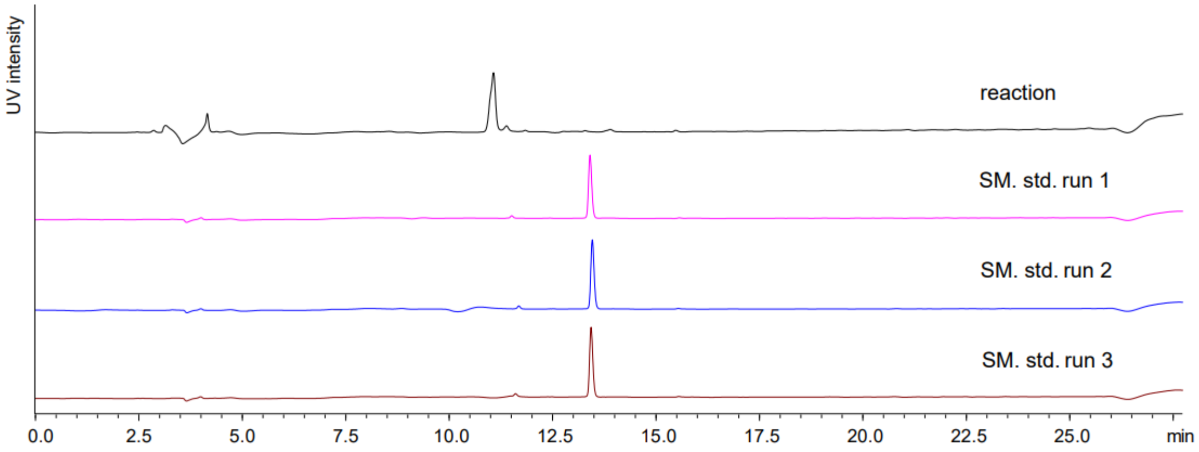

	peak area	average of run 1, 2, 3	yield
reaction (10 mM)	4350550	-	85%
SM std run 1 (10 mM)	4777250	5092865	-
SM std run 2 (10 mM)	5250448		
SM std run 3 (10 mM)	5250897		

$$\text{yield} = \frac{\text{peak area (product)}}{\text{peak area average (SM std)}} \div \frac{\text{concentration (reaction)}}{\text{concentration (SM std)}} \times 100\%$$

$$= \frac{4350550}{5092865} \div \frac{10}{10} \times 100\% = 85\%$$

Figure S5. Sample calculation of yield calculated by analytical RP-HPLC (5-70% MeCN over 21 min at 220 nm).

HPLC quantification for the optimization of N-H cross-coupling (Table S2)

((4-Methoxyphenyl)imino)dimethyl-λ^6-sulfanone **S2** was prepared as an internal standard. As peptide backbone or amino acid side chains do not show UV absorbance over 300 nm, yields of product **1d** are calculated based on absorbance at 310 nm relative to the internal standard **S2**.

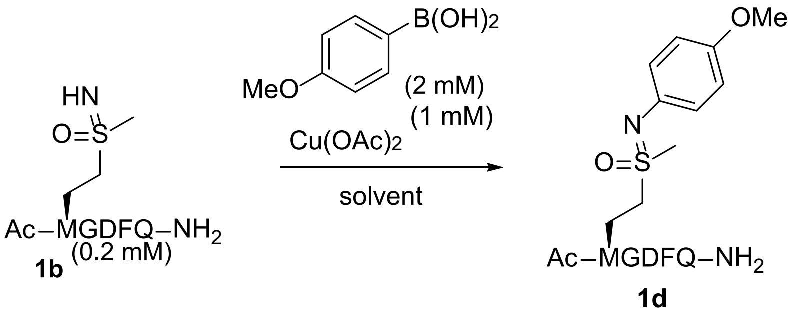

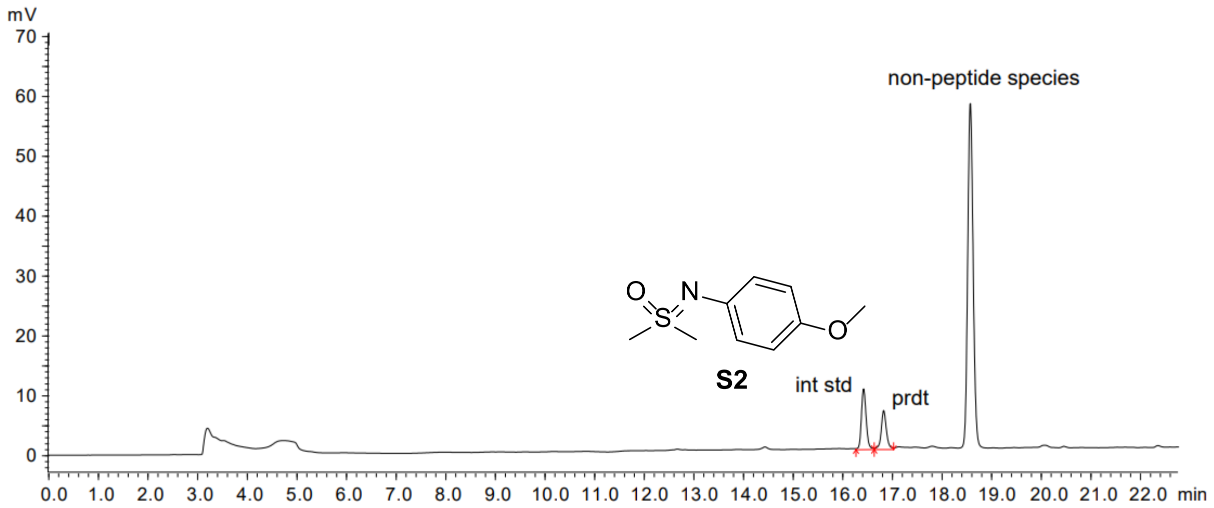

	peak area	yield
product (0.2 mM)	42348	62%
int std (0.2 mM)	68081	-

$$\text{yield} = \frac{\text{peak area (product)}}{\text{peak area (int std)}} \div \frac{\text{concentration (reaction)}}{\text{concentration (int std)}} \times 100\%$$

$$= \frac{42348}{68081} \div \frac{0.2}{0.2} \times 100\% = 62\%$$

Figure S6. Sample calculation of yield calculated by analytical RP-HPLC (5-50% MeCN over 18 min at 310 nm) with ((4-methoxyphenyl)imino)dimethyl-λ^6-sulfanone **S2** as an internal standard.

HPLC quantification for the boronic acid scope investigation of N-H cross-coupling (Figure 2)

A dabsyl-labeled methionine sulfoximine peptide **4** was prepared and used as the model for boronic acid scope investigation. Dabsyl glycine was used as an internal standard. Apart from the dabsyl group, peptide backbone, amino acid side chains or coupling reagents do not show UV absorbance over 500 nm, so product yields and starting material conversions could be reliably determined by comparing peak areas to that of the internal standard dabsyl glycine.

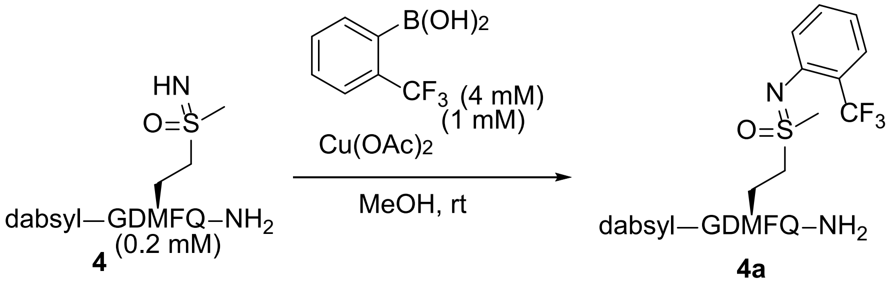

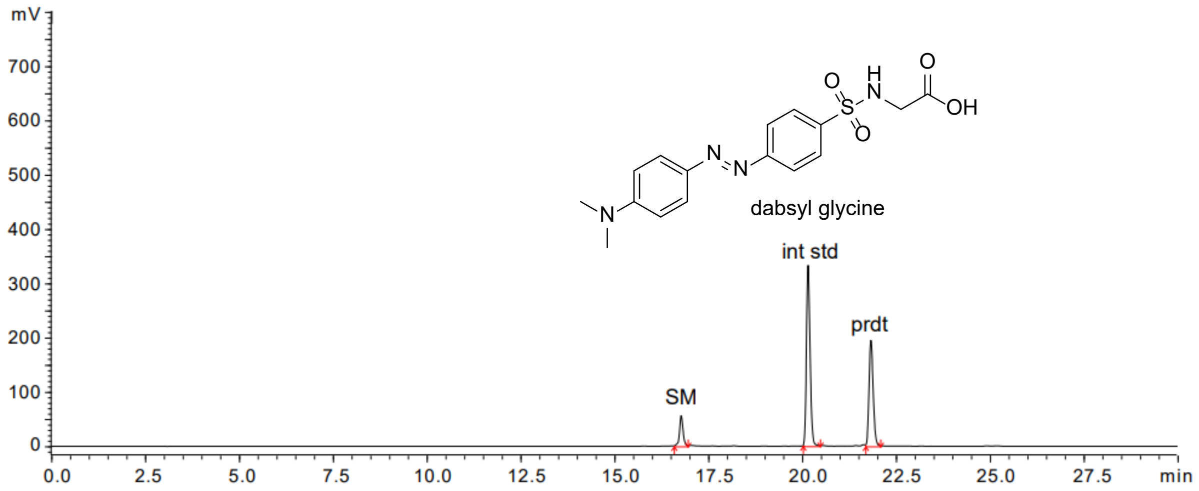

	peak area	yield
product (0.2 mM)	322490	80%
int std (0.2 mM)	404746	-

$$\text{yield} = \frac{\text{peak area (product)}}{\text{peak area (int std)}} \div \frac{\text{concentration (reaction)}}{\text{concentration (int std)}} \times 100\%$$

$$= \frac{322490}{404746} \div \frac{0.2}{0.2} \times 100\% = 80\%$$

Figure S7. Sample calculation of yield calculated by analytical RP-HPLC (5-70% MeCN over 21 min at 500 nm) with dabsyl glycine as an internal standard.

HPLC quantification for the N-H cross-coupling of methionine sulfoximine peptides with 4-nitrophenylboronic acid (Table 2)

Dimethyl((4-nitrophenyl)imino)-λ^6-sulfanone **S3** was prepared as an internal standard. As peptide backbone or amino acid side chains do not show UV absorbance over 300 nm, so product yields could be reliably determined by comparing peak areas to that of the internal standard **S3.**

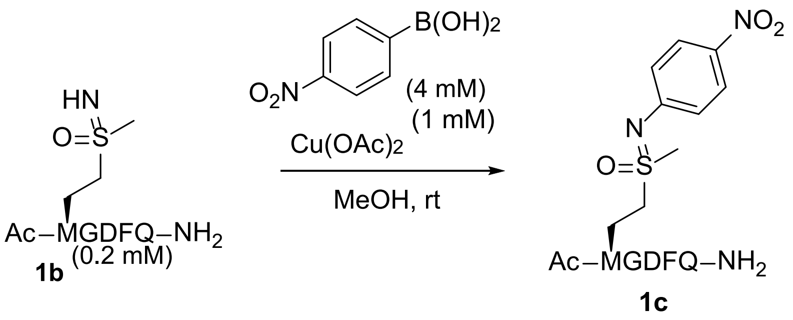

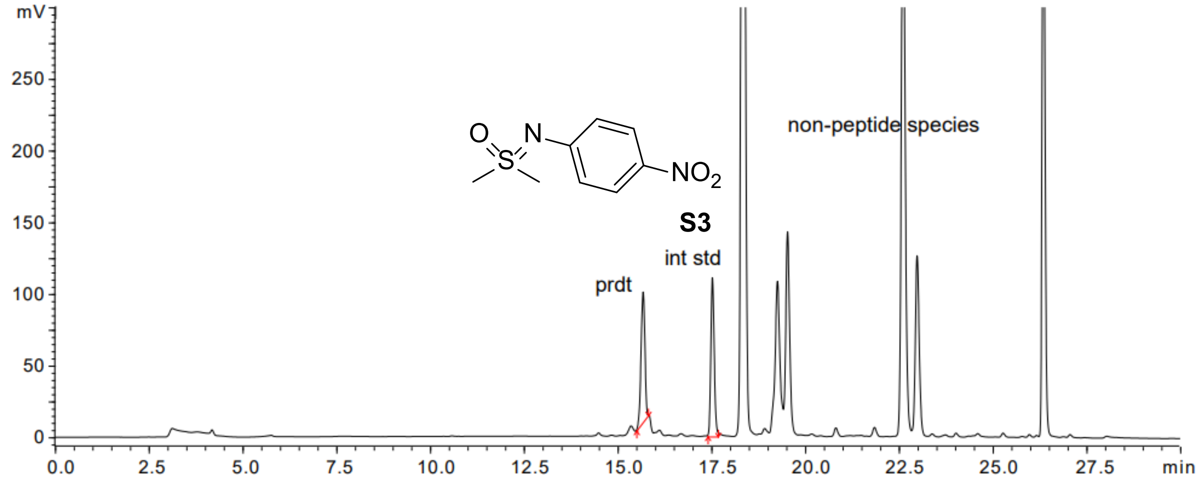

	peak area	yield
product	650991	94%
int std	692074	-

$$\text{yield} = \frac{\text{peak area (product)}}{\text{peak area (int std)}} \div \frac{\text{concentration (reaction)}}{\text{concentration (int std)}} \times 100\%$$

$$= \frac{650991}{692074} \div \frac{0.2}{0.2} \times 100\% = 94\%$$

Figure S8. Sample calculation of yield calculated by analytical RP-HPLC (5-70% MeCN over 21 min at 350 nm) with dimethyl((4-nitrophenyl)imino)-λ^6-sulfanone **S3** as an internal standard.

HPLC quantification for the NH-alkylation of methionine sulfoximine peptide (Figure 3, b)

Dabsyl-labeled methionine sulfoximine peptide **4** and dabsyl glycine were used as the substrate and internal standard for N–H-alkylation, respectively. The reaction mixture with internal standard was analyzed by RP-HPLC at 500 nm.

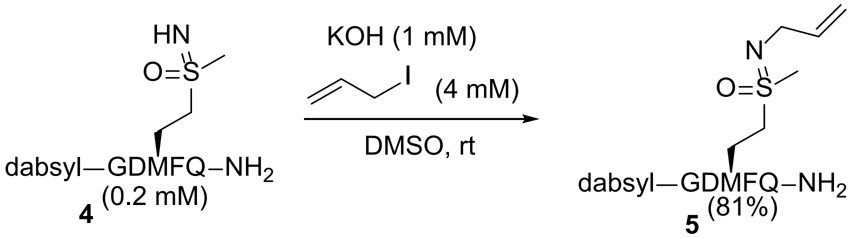

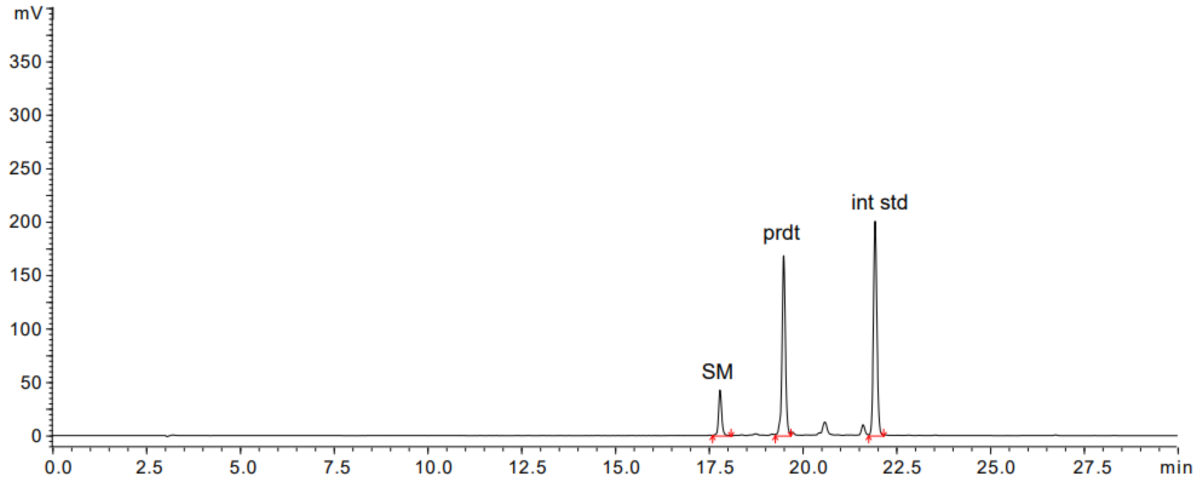

	peak area	yield
product	1065846	81%
int std	1313542	-

$$\text{yield} = \frac{\text{peak area (product)}}{\text{peak area (int std)}} \div \frac{\text{concentration (reaction)}}{\text{concentration (int std)}} \times 100\%$$

$$= \frac{1065846}{1313542} \div \frac{0.2}{0.2} \times 100\% = 81\%$$

Figure S9. Sample calculation of yield calculated by analytical RP-HPLC (5-65% MeCN over 21 min at 500 nm) with dabsyl glycine as an internal standard.

HPLC quantification for the carbonylation and ^{13}C labeling of methionine sulfoximine peptide (Figure 3, c)

The dimethyl sulfoximine **S4** was used as an internal standard. As peptide backbone or amino acid side chains do not show UV absorbance over 300 nm, product yields could be reliably determined by comparing peak areas to that of the internal standard **S4.**

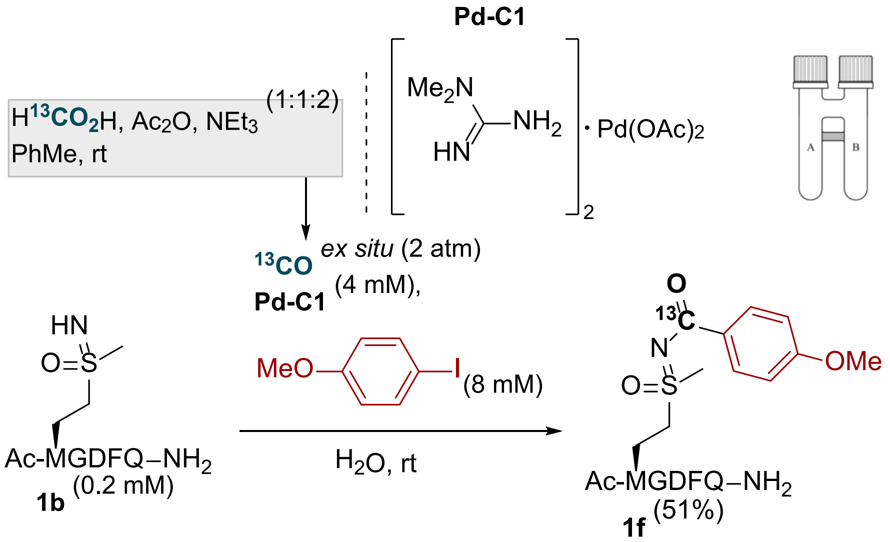

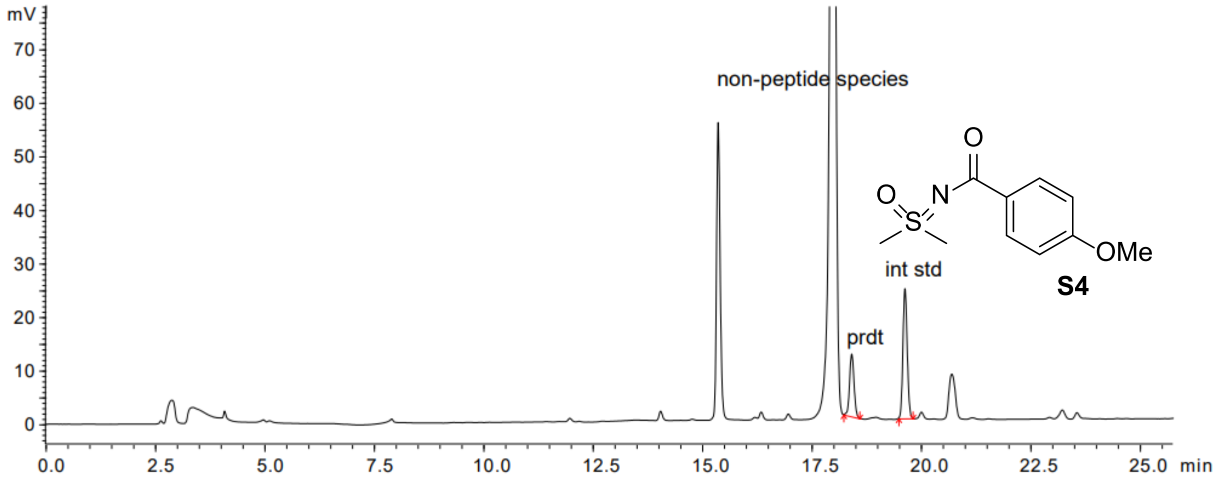

	peak area	yield
product	83030	51%
int std	164203	-

$$\text{yield} = \frac{\text{peak area (product)}}{\text{peak area (int std)}} \div \frac{\text{concentration (reaction)}}{\text{concentration (int std)}} \times 100\%$$

$$= \frac{83030}{164203} \div \frac{0.2}{0.2} \times 100\% = 51\%$$

Figure S10. Sample calculation of yield calculated by analytical RP-HPLC (5-50% MeCN over 21 min at 300 nm) with *N*-(dimethyl(oxo)-λ^6-sulfaneylidene)-4-methoxybenzamide **S4** as an internal standard.

Experimental Procedures

Peptide synthesis:

Besides α-MSH, all other peptides were synthesized using standard solid phase peptide synthesis protocols[2] using rink amide resin (P3Biosystems, 52001) and Fmoc-amino acids purchased from NovaBiochem and used without further purification. Peptides were purified by reverse-phase HPLC and characterized by ESI-MS.

Cysteine tolerance test

N-Acetyl-L-cysteine (16.3 mg, 0.10 mmol), $PhI(OAc)_2$ (80.5 mg, 0.25 mmol) and ammonium carbamate (15.6 mg, 0.20 mmol) were dissolved in MeOH (0.5 mL), and the resulting solution was stirred vigorously at room temperature for 3 h, and analyzed by LC-MS without purification.

General procedure for the optimization of methionine sulfoximidation (Table S1)

Peptide **2a** (6.51 mg, 0.010 mmol), hypervalent iodine reagent (0.025-0.10 mmol), and solid ammonium carbamate (0.1-0.5 mmol) were dissolved in solvent (1 mL), and the resulting solution was stirred vigorously at room temperature for 16 h. The reactions were quenched with a solution of phenyl disulfide in MeOH (1 M, 0.1 mL). To the reaction mixture was added 4-fluoro-2-nitrobenzaldehyde as an internal standard (40 μL, 50 mM in DMSO) and the yield was determined by RP-HPLC analysis (5-70% MeCN over 21 min) at 220 nm.

General procedure for methionine sulfoximidation in polypeptides using $PhI(OAc)_2$ and NH_2COONH_4 (Table 1)

General procedure A: peptide **1a**, **3a-6a** (0.01 mmol), $PhI(OAc)_2$ (8.1 mg, 0.025 mmol) and NH_2COONH_4 (15.6 mg, 0.20 mmol) were dissolved in MeOH (1 mL), and the resulting solution was stirred vigorously at room temperature for 16 h. The reactions were quenched with a solution of phenyl disulfide in MeOH (1 M, 0.1 mL). The yield was determined by RP-HPLC analysis (5-70% MeCN over 21 min or 5-50% MeCN over 21 min) at 220 nm by using the peptide starting material (known concentration) as an external standard. The product was then purified by RP-HPLC for preparatory scale (5-50% MeCN over 21 min) to afford the methionine sulfoximine peptides **1b**, **3b-6b** for future use.

General procedure B: peptide **2a**, **7a-8a** (0.01 mmol), $PhI(OAc)_2$ (8.1 mg, 0.025 mmol) and NH_2COONH_4 (15.6 mg, 0.20 mmol) were dissolved in aq MeOH (1 mL, 80% v/v soln), and the resulting solution was stirred vigorously at rt for 16 h. The reactions were quenched with a solution of phenyl disulfide in MeOH (1 M, 0.1 mL). The yield was determined by RP-HPLC analysis (5-70% MeCN over 21 min or 5-50% MeCN over 21 min) at 220 nm. The product was then purified by RP-HPLC for preparatory scale (5-50% MeCN over 21 min) to afford the methionine sulfoximine peptides **2b**, **7b-8b** for future use.

Preparation of dabsyl-labeled methionine sulfoximine peptide 4

In solid phase peptide synthesis, dabsyl chloride was reacted with the free N-terminus on resin using a previously reported protocol.[3] The cleaved and purified dabsyl-labeled peptide (8.8 mg, 0.01 mmol) in methanol (1.0 mL) was then treated with $PhI(OAc)_2$ (8.1 mg, 0.025 mmol) and NH_2COONH_4 (15.6 mg, 0.20 mmol), and the resulting mixture was stirred vigorously at room temperature for 16 h. The reactions were quenched with a solution of phenyl disulfide in MeOH (1 M, 0.1 mL). The reaction mixture was then directly injected onto a RP-HPLC column for preparative-scale purification (5-70% MeCN over 21 min) to afford the dabsyl-labeled methionine sulfoximine peptide **4** (5.8 mg, 63%) for boronic acid screen.

General procedure for the optimization of N-H cross-coupling (Table S2)

Methionine sulfoximine peptide **1b** (1.0 µL, 10 mM in H_2O) was incubated with 4-methoxyphenylboronic acid **h** (2.0 µL, 50 mM in DMSO) and $Cu(OAc)_2$ (1.0 µL, 50 mM in H_2O) in solvent (46 µL) at room temperature or 37 °C for 16 h. The reactions were quenched with EDTA (1 µL, 0.1 M aq soln). To the reaction mixture was added ((4-methoxyphenyl)imino)dimethyl-λ^6-sulfanone **S2** as an internal standard (1 µL, 10 mM in DMSO) and the yield was determined by RP-HPLC analysis (5-50% MeCN over 18 min) at 310 nm.

General procedure for the metal salt screen of N-H cross-coupling (Table S3)

Methionine sulfoximine peptide **1b** (1.0 µL, 10 mM in H_2O) was incubated with 4-methoxyphenylboronic acid **h** (2 µL, 50 mM in DMSO) and metal salts (1.0 µL, 50 mM in H_2O) in MeOH (46 µL) at room temperature for 16 h. The reactions were quenched with EDTA (1 µL, 0.1 M aq soln). The reaction mixture was then directly used for MALDI-MS analysis.

Preparative scale of N-H cross-coupling of methionine sulfoximine peptide 1b with 4-methoxyphenylboronic acid (Figure 1, a)

Methionine sulfoximine peptide **1b** (6.7 mg, 0.010 mmol), 4-methoxyphenylboronic acid **h** (7.6 mg, 0.050 mmol) and copper(II) acetate monohydrate (2.0 mg, 0.010 mmol) were dissolved in MeOH (1 mL), and the resulting solution was stirred vigorously at room temperature for 16 h. The product was then purified by RP-HPLC for preparatory scale (5-50% MeCN over 21 min) to afford the arylated methionine sulfoximine peptide **1d** (4.1 mg, 53%).

General procedure for the boronic acid scope investigation of N-H cross-coupling (Figure 2)

Dabsyl-labeled methionine sulfoximine peptide **4** (0.5 µL, 10 mM in DMSO) was incubated with boronic acid **a-q** (1 µL, 100 mM in DMSO) and $Cu(OAc)_2$ (0.5 µL, 50 mM in H_2O) in MeOH (23 µL) at room temperature for 16 h. The reactions were quenched with EDTA (1 µL, 0.1 M aq soln). To the reaction mixture was added dabsyl glycine as an internal standard (0.5 µL, 10 mM in DMSO) and the yield was determined by RP-HPLC analysis (5-70% MeCN over 21 min or 5-65% MeCN over 21 min) at 500 nm.

General procedure for the copper-mediated N-H cross-coupling of methionine sulfoximine peptides with 4-nitrophenylboronic acid (Table 2)

Methionine sulfoximine peptide **1b-8b** (0.5 µL, 10 mM in H_2O) was incubated with 4-nitrophenylboronic acid **b** (1 µL, 100 mM in DMSO) and $Cu(OAc)_2$ (0.5 µL, 50 mM in H_2O) in MeOH (23 µL) at room temperature for 16 h. The reactions were quenched with EDTA (1 µL, 0.1 M aq soln). To the reaction mixture was added dimethyl((4-nitrophenyl)imino)-λ^6-sulfanone **S3** as an internal standard (0.5 µL, 10 mM in DMSO) and the yield was determined by RP-HPLC analysis at 350 nm.

General procedure for preparative scale of N-H cross-coupling of methionine sulfoximine peptides with 4-nitrophenylboronic acid (Table 2)

Methionine sulfoximine peptide **7b-8b** (50 µL, 10 mM in H_2O) was incubated with 4-nitrophenylboronic acid **b** (100 µL, 100 mM in DMSO) and $Cu(OAc)_2$ (50 µL, 50 mM in H_2O) in MeOH (2.3 mL) at room temperature for 16 h. Coupling products were then purified by RP-HPLC (5-55% MeCN over 21 min) to afford **7c** (0.30 mg, 39%) and **8c** (0.39 mg, 43%).

Procedure for the N–H cross-coupling of methionine sulfoximine peptide 1b with potassium 3-(azidomethyl)phenyltrifluoroborate (Figure 3, a)

Methionine sulfoximine peptide **1b** (0.5 µL, 10 mM in H_2O) was incubated with potassium 3-(azidomethyl)phenyltrifluoroborate (1 µL, 100 mM in DMSO) and $Cu(OAc)_2$ (0.5 µL, 50 mM in H_2O) in MeOH (23 µL) at room temperature for 16 h. The reaction was quenched with EDTA (1 µL, 0.1 M aq soln), and analyzed by LC-MS and RP-HPLC (5-50% MeCN over 18 min at 254 nm).

Procedure for the NH-alkylation of methionine sulfoximine peptide (Figure 3, b)

Dabsyl-labeled methionine sulfoximine peptide **4** (0.5 µL, 10 mM in DMSO) was incubated with allyl iodide (1 µL, 100 mM in DMSO) and KOH (0.5 µL, 50 mM in DMSO) in DMSO (23 µL) at room temperature for 16 h. To the reaction mixture was added dabsyl glycine as an internal standard (0.5 µL, 10 mM in DMSO) and the yield was determined by RP-HPLC analysis (5-65% MeCN over 21 min) at 500 nm.

Procedure for the carbonylation and ^{13}C labeling of methionine sulfoximine peptide (Figure 3, c)

To a cut-open Eppendorf tube in chamber A of a two-chamber system (total volume = 7 mL) was added MilliQ water (12 µL), **Pd-C1** (10 µL, 10 mM in H_2O, 4.0 mM final concn), 4-iodoanisole (2.0 µL, 100 mM in DMSO, 8.0 mM final concn) and sulfoximine peptide (1.0 µL, 5 mM, 0.2 mM final concn). The chamber was sealed with a screwcap fitted with two silicone seals. To chamber B was added a magnetic stir bar, toluene (0.85 mL), [^{13}C]-HCO_2H or [^{12}C]-HCO_2H (21.6 µL, 0.573 mmol), Ac_2O (54.1 µL, 0.573 mmol) and lastly Et_3N (160 µL, 1.15 mmol) was carefully layered on top of the toluene. The chamber was then quickly sealed with a screwcap fitted with two silicone seals. Stirring was then initiated, and extensive bubbling was observed almost immediately in chamber B, indicating CO evolution. Stirring in chamber B only was continued overnight at rt. Thereafter, N-(dimethyl(oxo)-λ^6-sulfaneylidene)-4-methoxybenzamide **S4** (0.5 µL, 10 mM, 0.2 mM final concn) was added to the reaction mixture, and the yield was determined by RP-HPLC analysis (5-50% MeCN over 21 min) at 300 nm.

Handling of carbon monoxide

All carbonylation reactions were performed in a two-chamber system using adaptations of protocols that have been developed and tested elsewhere.[4] In this system, gaseous CO is released in one chamber and the carbonylative coupling occurs in a second chamber. The two-chamber system (COWare®) is depicted to the right and is composed of two glass vials (Chamber A and B) connected with a glass tube to allow gas-transfer (Total Volume = 7 mL (small scale) or 20 mL (large scale)). The chambers can be sealed with a screw cap and a Teflon® coated silicone seal. Small scale reactors are sealed with 2 silicone seals per chamber. CO-gas was released from formic acid[5] at rt under air and moisture.

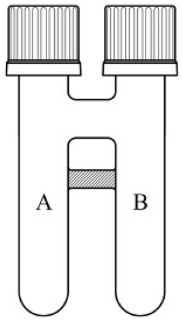

WARNING: Glassware under pressure!

- Glass equipment should always be examined for damages to its surface, which may weaken its strength.
- One must abide to all laboratory safety procedures and always work behind a shield when working with glass equipment under pressure.

COware is pressure tested to 224 psi, but should under no circumstances be operated above 60 psi (5 bar).

Procedure for the glutamine synthetase inhibition assay (Figure 4)

In this assay, glutamine synthetase catalyzes the transformation from glutamate and ATP to glutamine and ADP. ADP in the subsequent reaction, in presence of ADP converter, ADP developer and ADP probe, forms a colorimetric product that is measured at absorbance 570 nm.

Detailed procedure:

1. Stock solutions of glutamine synthetase, ATP, ADP converter, and ADP developer were prepared according to the protocol by Abcam:

https://www.abcam.com/ps/products/284/ab284572/documents/Glutamine-Synthetase-Activity-Assay-protocol-book-v1c-ab284572%20(website).pdf

GS assay buffer, glutamate, and ADP probe were used as supplied.

2. Glutamine synthetase (2 µL) was pre-incubated with methionine sulfoximine (MSO) (2 µL, 10 mM, 1 mM final concn) and methionine sulfoximine peptides **1b-8b** (2 µL, 10 mM, 1 mM final concn) at 37 ºC for 10 min, protected from light. For enzyme control, GS assay buffer (2 µL) was used instead of MSO or peptides **1b-8b**. For background control, GS assay buffer (4 µL) without enzyme was used.

3. Substrate mix was prepared by mixing GS assay buffer (96 µL), glutamate (24 µL), and ATP (24 µL). The resulting substrate mix (12 µL) was added to each sample.

4. ADP reaction mix was prepared by mixing GS assay buffer (42 µL), ADP probe (6 µL), ADP converter (6 µL), and ADP developer (6 µL). The resulting ADP reaction mix (4 µL) was added to each sample. The total reaction volume for each sample is 20 µL.

5. After incubation of the reaction mixture at 37 ºC for 60 min, the absorbance at 570 nm (A_{570}) was measured on a Thermo Scientific NanoDrop 2000c spectrophotometer. For each sample, A_{570} was measure three times for average.

6. The background control reading was subtracted from test sample readings and enzyme control readings.

7. Inhibition of glutamine synthetase was determined by comparing A_{570} (test sample) and A_{570} (enzyme control):

$$\text{Percentage inhibition} = \frac{\text{A570 (enzyme control)} - \text{A570 (test sample)}}{\text{A570 (enzyme control)}} \times 100\%$$

8. The assay was repeated for 3 times and the percentage inhibition values are mean ± SD of 3 independent experiments.

Preparation of Reagents

Synthesis of known compounds

Ion-supported $PhI(OAc)_2$ analogue *N*-Methyl-*N*-[3-(4'-diacetoxyiodo)phenoxy-1-propyl]pyrrolidinium 4''-Methylbenzenesulfonate **S1** was prepared according to a previously reported protocol.[1]

Boronic acids *N*-(prop-2-yn-1-yl)-2-(4,4,5,5-tetramethyl-1,3,2-dioxaborolan-2-yl)benzenesulfonamide **p** and (4-(but-3-yn-1-ylcarbamoyl)-2-(N-methylsulfamoyl)phenyl)boronic acid **q** were prepared according to a previously reported protocol.[6]

((4-methoxyphenyl)imino)dimethyl- λ^6-sulfanone (S2)

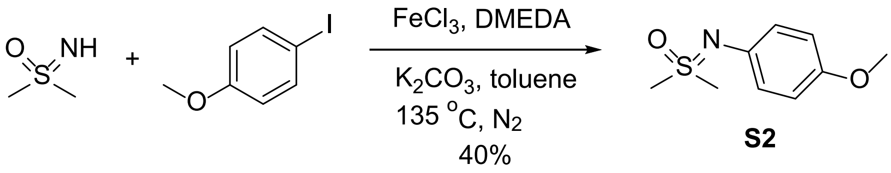

S2

The synthesis was conducted by adapting a reported protocol.[7] A sealable 4 mL vial was charged with iminodimethyl-λ^6-sulfanone (46.6 mg, 0.5 mmol), anhydrous $FeCl_3$ (16.2 mg, 0.1 mmol) and K_2CO_3 (138.2 mg, 1.0 mmol), and a nitrogen atmosphere was established. 4-Iodoanisole (101.6 µL, 0.75 mmol), *N,N'*-dimethylethylenediamine (DMEDA) (21.8 µL, 0.2 mmol) and toluene (1 mL) were added via syringe. The vial was placed in a 135 °C oil bath. After stirring at this temperature for 24 h, the heterogeneous mixture was cooled to room temperature and diluted with dichloromethane. The resulting solution was directly filtered through a pad of celite and concentrated to deliver the product, which was purified by silica gel chromatography (hexane/EtOAc, 1:1) to yield **S2** as a white solid (39.9 mg, 40%). Spectral data is consistent with that previously reported.[8] ^{1}H NMR (600 MHz, $CDCl_3$) δ 7.02 – 6.99 (m, 2H), 6.81 – 6.78 (m, 2H), 3.76 (s, 3H), 3.11 (s, 6H). ^{13}C NMR (151 MHz, $CDCl_3$) δ 156.2, 135.5, 125.3, 114.7, 55.6, 41.5.

Dimethyl((4-nitrophenyl)imino)-λ^6-sulfanone (S3)

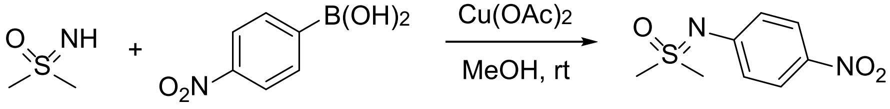

S3

The synthesis was conducted by adapting a reported protocol.[9] Iminodimethyl-λ^6-sulfanone (46.6 mg, 0.5 mmol), 4-nitrophenylboronic acid (200.3 mg, 1.2 mmol) and copper(II) acetate monohydrate (10.0 mg, 0.05 mmol) were dissolved in MeOH (2 mL). After stirring vigorously at room temperature for 16 h, the solvent was removed under reduced pressure. The resulting residue was purified by silica gel column chromatography (hexane/EtOAc, 3:7) to afford **S3** as a light-yellow solid (59.7 mg, 56%). Spectral data is consistent with that previously reported.[10] ^{1}H NMR (600 MHz, $CDCl_3$) δ 8.12 (d, *J* = 9.0 Hz, 2H), 7.12 (d, *J* = 9.0 Hz, 2H), 3.25 (s, 6H). ^{13}C NMR (151 MHz, $CDCl_3$) δ 151.4, 142.6, 125.5, 122.4, 42.9.

***N*-(Dimethyl(oxo)-λ^6-sulfaneylidene)-4-methoxybenzamide (S4)**

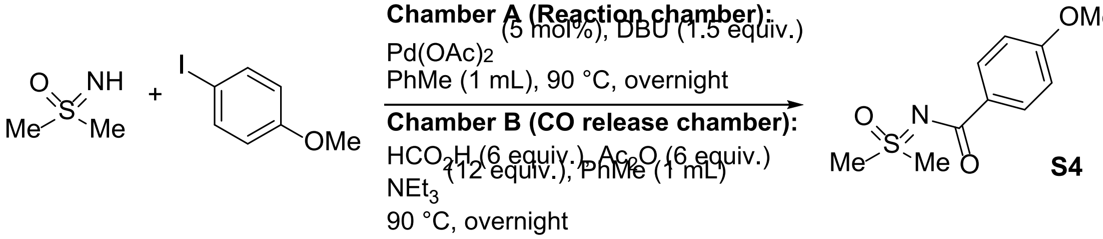

The synthesis was conducted by adapting a reported protocol.[11] To chamber A of a two-chamber system (20 mL total volume) was added iminodimethyl-λ^6-sulfanone (37 mg, 0.40 mmol), 4-iodoanisole (112 mg, 0.480 mmol, 1.2 equiv.), $Pd(OAc)_2$ (4.5 mg, 0.02 mmol, 5.0 mol%), toluene (1 mL) and lastly DBU (89.6 µL, 0.600 mmol, 1.5 equiv.). The chamber was sealed with a screwcap fitted with a Teflon® seal. To chamber B was added toluene (1 mL), HCO_2H (90.5 µL, 2.40 mmol, 6.0 equiv.) and Ac_2O (227 µL, 2.40 mmol, 6.0 equiv.). The chamber was sealed with a screwcap fitted with a pierceable Teflon® seal through which NEt_3 (669 µL, 4.80 mmol, 12 equiv.) was added to chamber B carefully on top of the toluene. The two-chamber system was heated to 90 °C in an oil bath and stirred overnight. The reaction mixture was subsequently passed through celite and purified by flash column chromatography (hexane/EtOAc 8:2 to pure EtOAc) to yield the product **S4** as a white solid (33 mg, 36%). ^{1}H NMR (600 MHz, $CDCl_3$) δ 8.08 (d, J = 8.9 Hz, 2H), 6.89 (d, J = 8.9 Hz, 2H), 3.86 (s, 3H), 3.38 (s, 6H). ^{13}C NMR (151 MHz, $CDCl_3$) δ 173.8, 163.0, 131.4, 128.2, 113.4, 55.5, 42.0. HRMS $C_{10}H_{14}NO_3S^+$ $[M+H]^+$ calcd 228.0689, found 228.0690.

Supplementary Data

Cysteine tolerance test

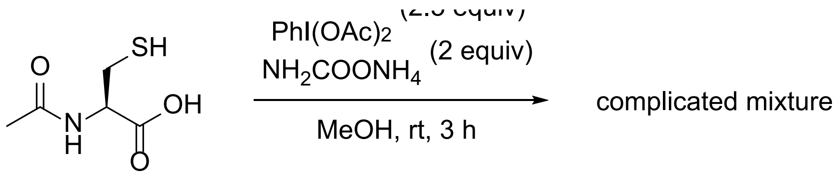

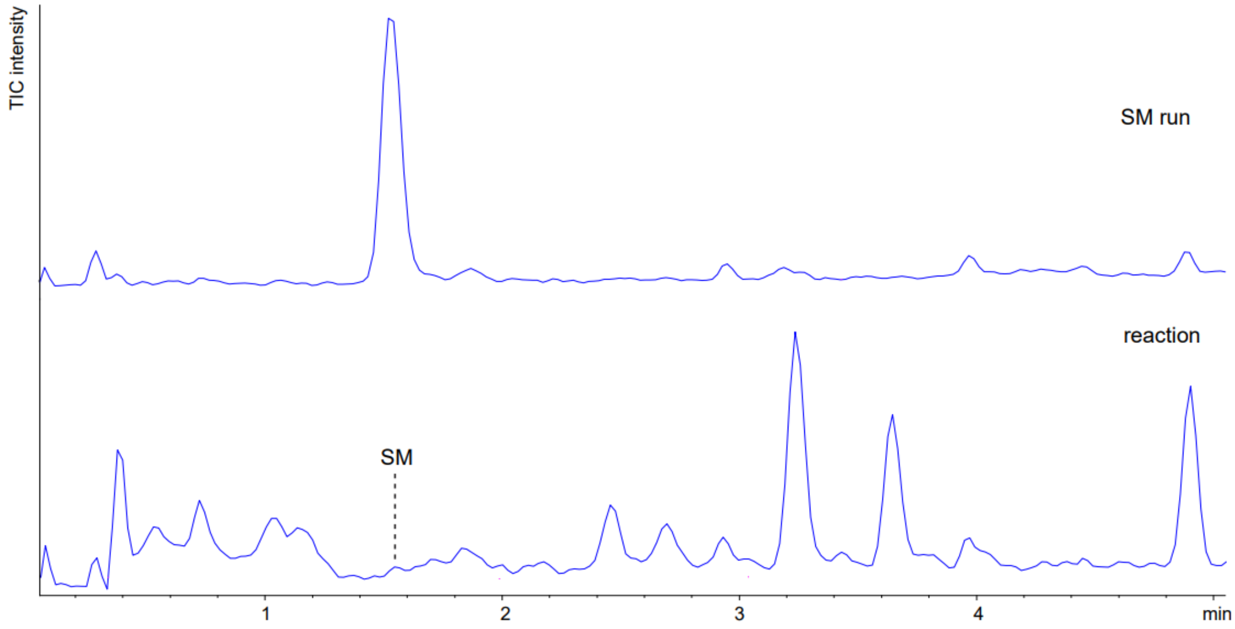

Figure S11. LC-MS trace (0-15% MeCN in H_2O over 5 min) of *N*-Acetyl-L-cysteine (above) and the cysteine oxidation reaction (below).

Table S1. Optimization of methionine sulfoximidation

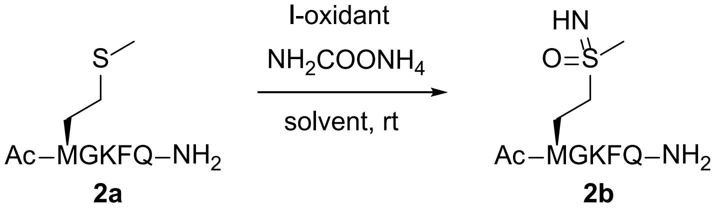

**Entry**	**Oxidant (eq)**	**NH_4COONH_2 (eq)**	**Solvent**	**Yield (%)**
1	$PhI(OAc)_2$ (2.5)	10	MeOH	18
2	$PhI(OAc)_2$ (2.5)	10	MeOH:H_2O 4:1	14
3	$PhI(OAc)_2$ (2.5)	20	MeOH:H_2O 4:1	50
3	$PhI(OAc)_2$ (2.5)	50	MeOH:H_2O 4:1	42
4	$PhI(OAc)_2$ (2.5)	20	MeOH:H_2O 9:1	35
5	$PhI(OAc)_2$ (2.5)	20	TFE	-
6	$PhI(OAc)_2$ (5.0)	20	MeOH:H_2O 4:1	39
7	$PhI(OAc)_2$ (10)	20	MeOH:H_2O 4:1	-
8	IBX (2.5)	20	MeOH:H_2O 4:1	<5
9	PhI(OH)(OTs) (2.5)	20	MeOH:H_2O 4:1	25
10	**S1** (2.5)	20	MeOH:H_2O 4:1	30
11	**S1** (2.5)	20	H_2O	-

Ion-supported $PhI(OAc)_2$ analogue **S1**:

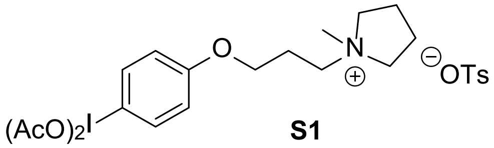

S1

Table S2. Optimization of N-H cross-coupling

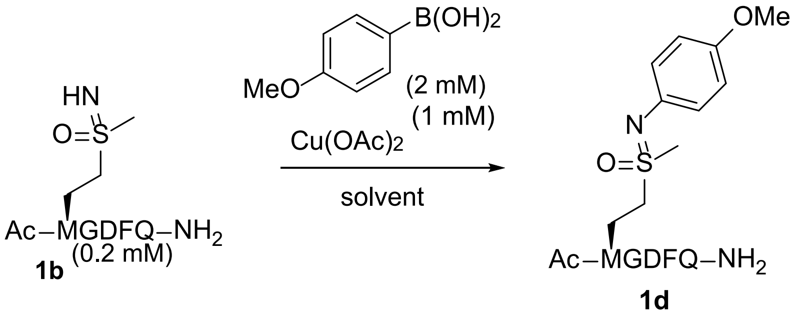

Entry	Solvent	Temp. (°C)	Yield (%)
1	MeOH	rt	62
2	MeOH	37	65
3	NMM (10 mM, pH 7.4)	rt	7
4	Tris (10 mM, pH 7.4)	rt	7
5	HEPES (10 mM, pH 7.4)	rt	-
6	NMM w/ 30% MeOH	rt	10
7	Tris w/ 30% MeOH	rt	9
8	HEPES w/ 30% MeOH	rt	-
9	MeOH w/ 20% NMM	rt	34
10	MeOH w/ 20% Tris	rt	20
11	MeOH w/ 20% HEPES	rt	-
12	MeOH w/ 20% H_2O	rt	66
13	MeOH w/ 50% H_2O	rt	33
14	MeOH w/ 70% H_2O	rt	19
15	H_2O	rt	7

Table S3. Metal salt screen of N-H cross-coupling

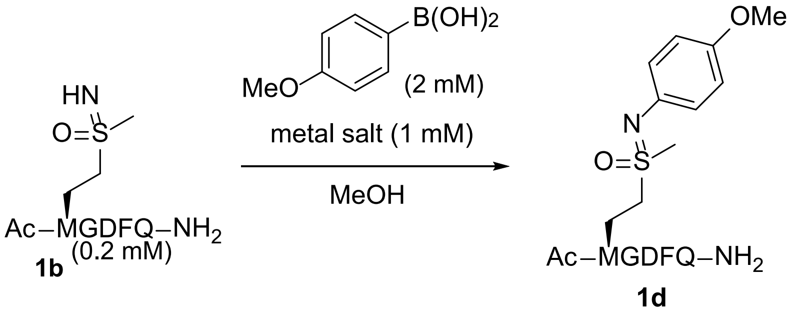

Entry	Metal	Yield (%)
1	$Cu(OAc)_2$	62
2	$Ni(OAc)_2$	-
3	$Co(OAc)_2$	-
4	$Mg(OAc)_2$	-
5	$CaCl_2$	-
6	$Mn(OAc)_2$	-
7	$Zn(OAc)_2$	-
8	AgOAc	-

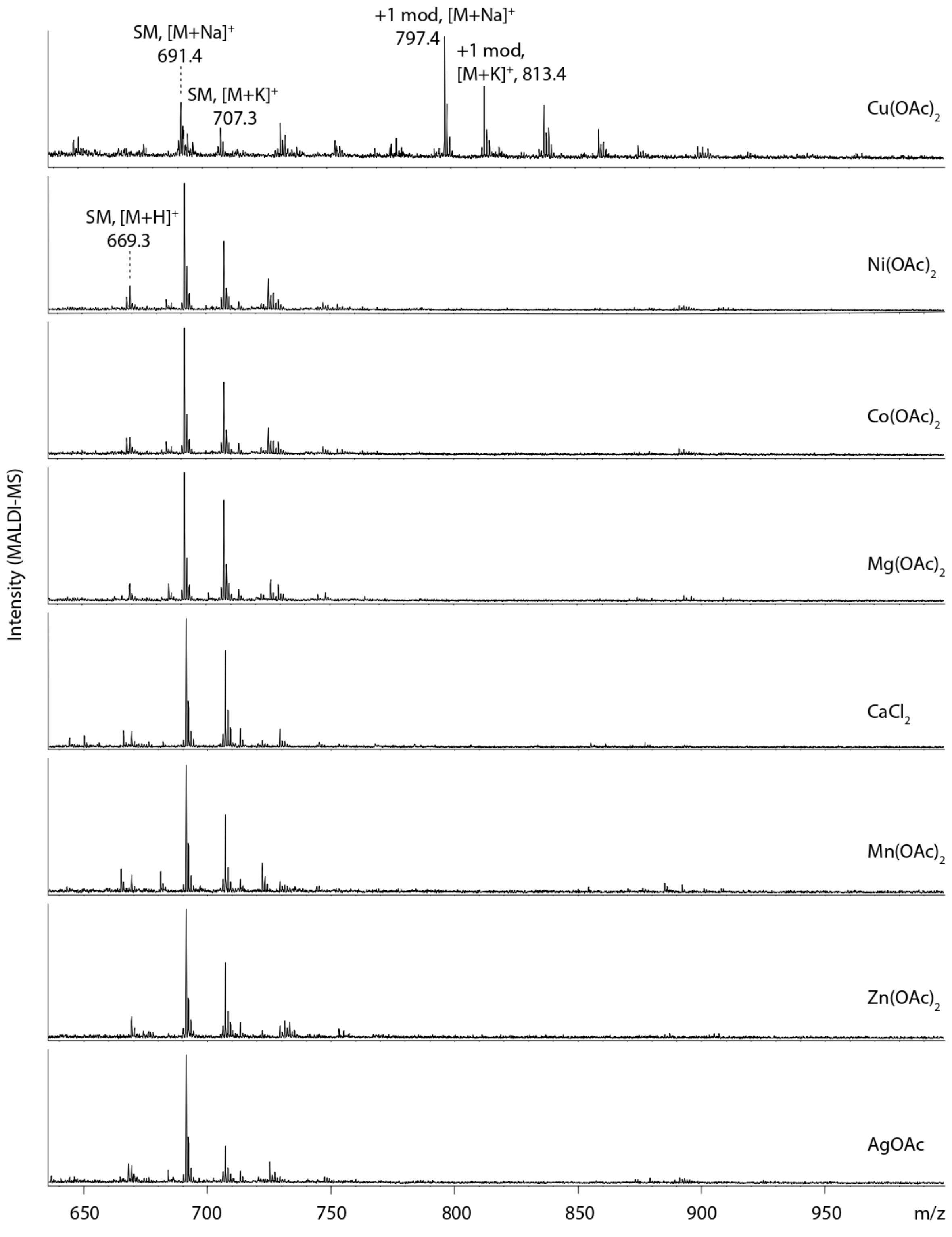

Figure S12. MALDI-MS spectra for the crude reaction mixture of **1b** with various metal salts and 4-methoxyphenylboronic acid **h**. Unmodified peptide was observed at 669.3 ([M+H]$^+$) and 691.4 ([M+Na]$^+$). Modified peptide **1d** was observed at 797.4 ([M+Na]$^+$) and 813.4 ([M+K]$^+$). α-Cyano-4-hydroxy-cinnamic acid (CHCA) was used as a matrix.

For Table 1

Synthesis of peptide 1b: Methionine sulfoximidation of peptide 1a

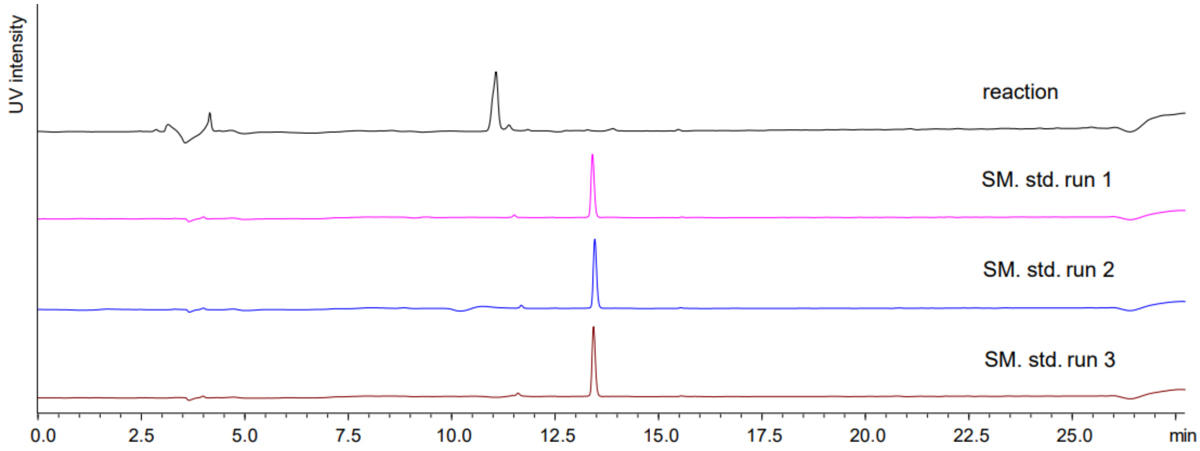

	peak area	average of run 1, 2, 3	yield
reaction (10 mM)	4350550	-	85%
SM std run 1 (10 mM)	4777250	5092865	-
SM std run 2 (10 mM)	5250448		
SM std run 3 (10 mM)	5250897		

Figure S13. RP-HPLC trace of the reaction of peptide **1a** at 220 nm (5-70% MeCN over 21 min).

Synthesis of peptide 2b: Methionine sulfoximidation of peptide 2a

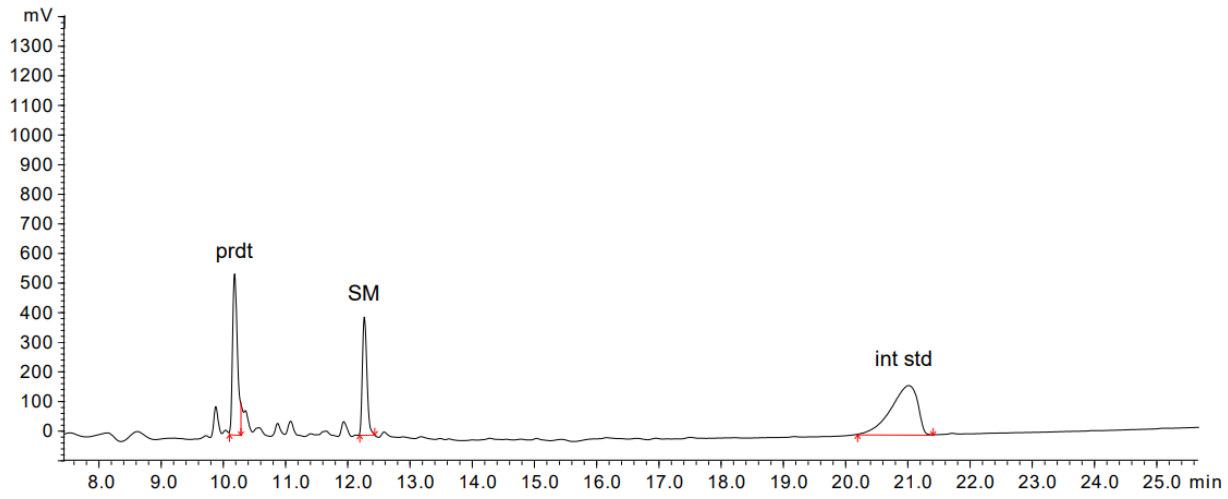

	peak area	yield
product	2948385	50%
int std	4901367	-

Figure S14. RP-HPLC trace of the reaction of peptide **2a** at 220 nm (5-70% MeCN over 21 min). Detailed calculations were shown in "sample calculation" section in page S4-S7.

Synthesis of peptide 3b: Methionine sulfoximidation of peptide 3a

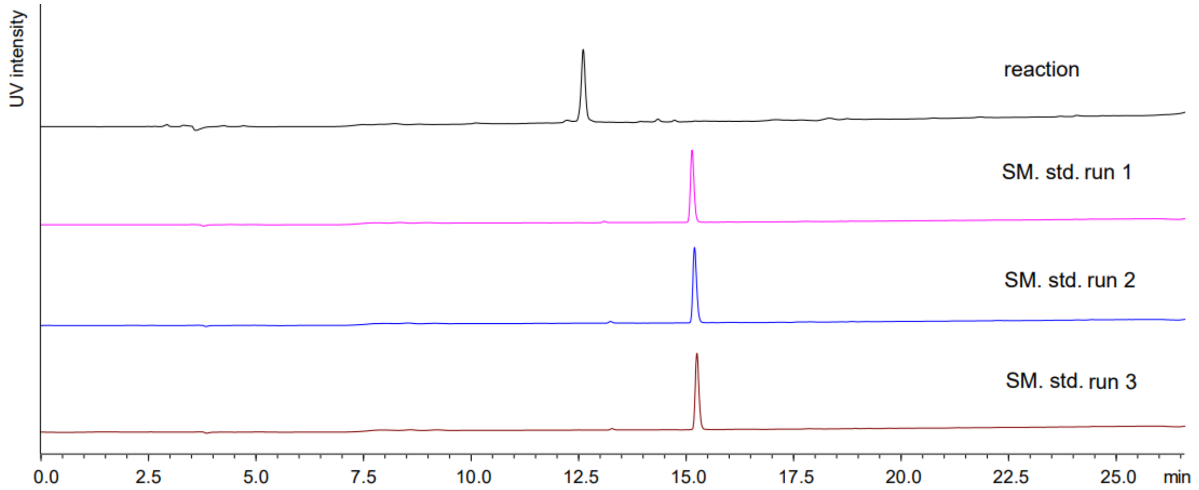

	peak area	average of run 1, 2, 3	yield
reaction (10 mM)	6861925	-	92%
SM std run 1 (10 mM)	7113274	7468865	-
SM std run 2 (10 mM)	7669384		
SM std run 3 (10 mM)	7623936		

Figure S15. RP-HPLC trace of the reaction of peptide **3a** at 220 nm (5-70% MeCN over 21 min).

Synthesis of peptide 4b: Methionine sulfoximidation of peptide 4a

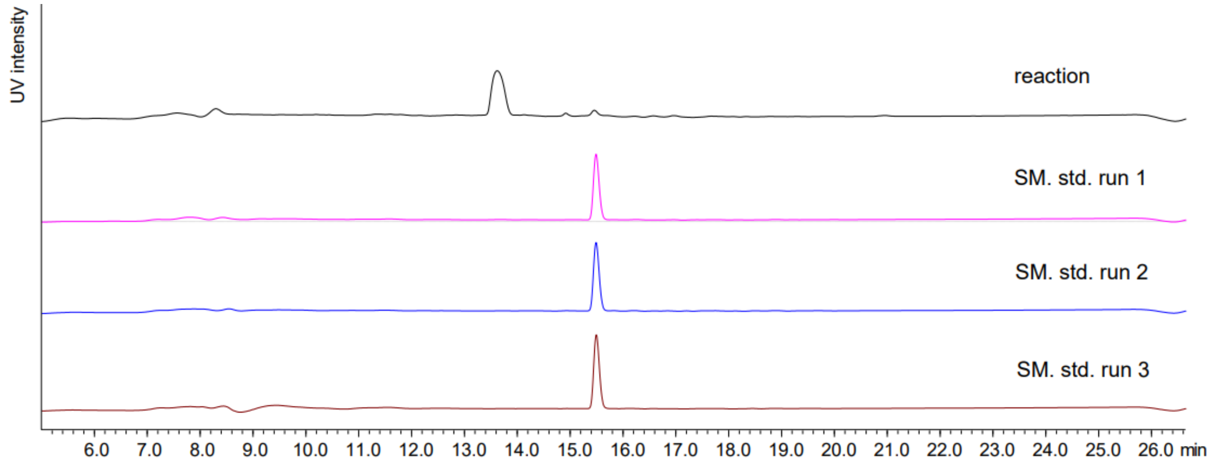

	peak area	average of run 1, 2, 3	yield
reaction (10 mM)	4499171	-	86%
SM std run 1 (10 mM)	5043057	5236859	-
SM std run 2 (10 mM)	5353343		
SM std run 3 (10 mM)	5314178		

Figure S16. RP-HPLC trace of the reaction of peptide **4a** at 220 nm (5-70% MeCN over 21 min).

Synthesis of peptide 5b: Methionine sulfoximidation of peptide 5a

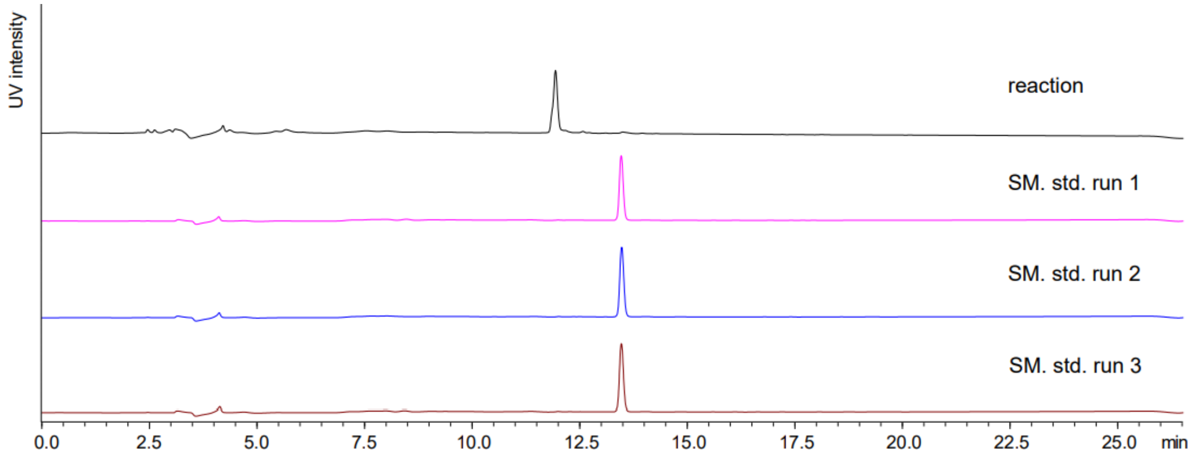

	peak area	average of run 1, 2, 3	yield
reaction (10 mM)	7976431	-	84%
SM std run 1 (10 mM)	8965167	9452987	-
SM std run 2 (10 mM)	9777533		
SM std run 3 (10 mM)	9616261		

Figure S17. RP-HPLC trace of the reaction of peptide **5a** at 220 nm (5-70% MeCN over 21 min).

Synthesis of peptide 6b: Methionine sulfoximidation of peptide 6a

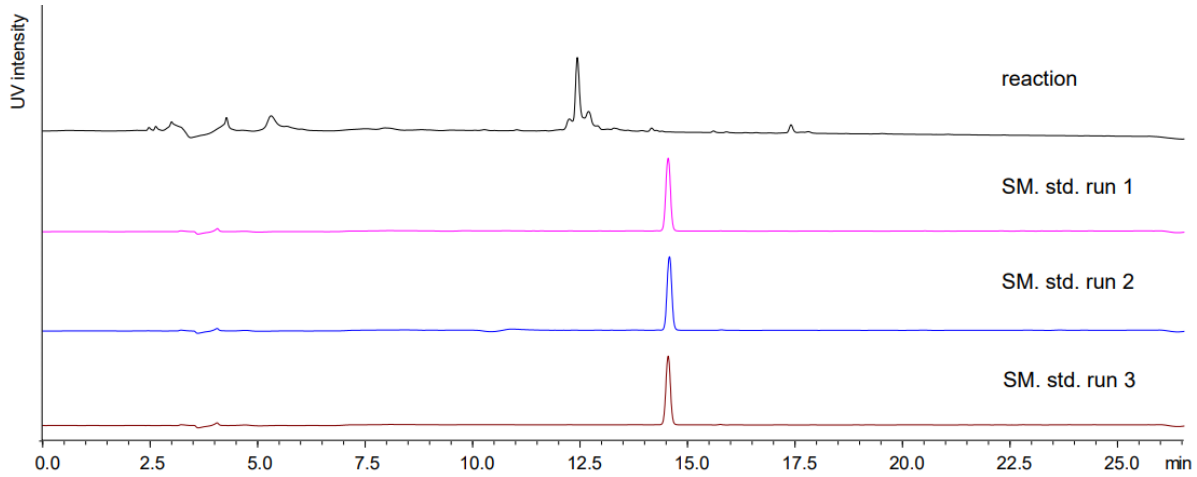

	peak area	average of run 1, 2, 3	yield
reaction (10 mM)	8764848	-	67%
SM std run 1 (10 mM)	13243586	13156016	-
SM std run 2 (10 mM)	13410189		
SM std run 3 (10 mM)	12814272		

Figure S18. RP-HPLC trace of the reaction of peptide **6a** at 220 nm (5-70% MeCN over 21 min).

Synthesis of peptide 7b: Methionine sulfoximidation of peptide 7a

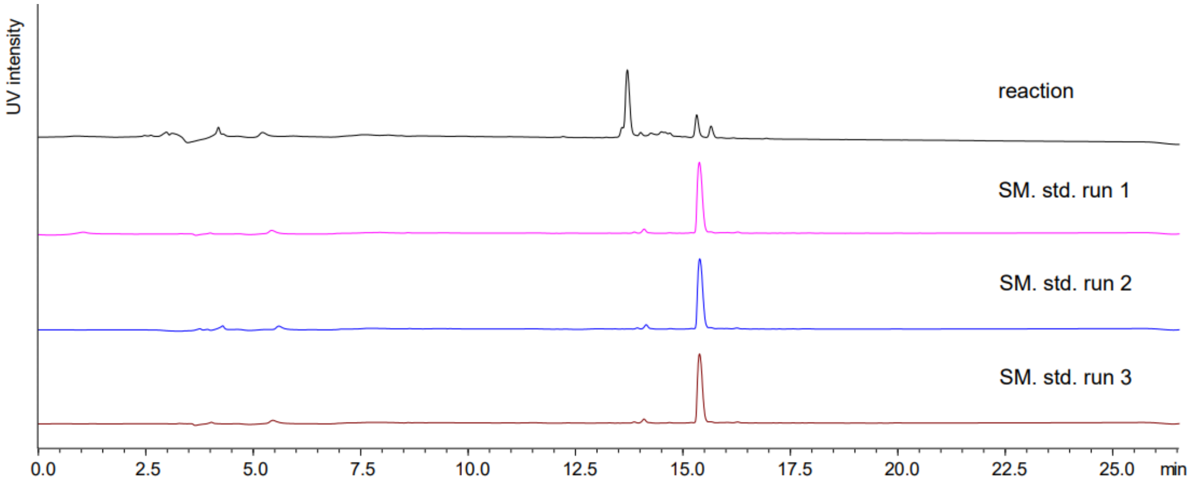

	peak area	average of run 1, 2, 3	yield
reaction (10 mM)	8946731	-	65%
SM std run 1 (10 mM)	13977460	13682784	-
SM std run 2 (10 mM)	13862734		
SM std run 3 (10 mM)	13208157		

Figure S19. RP-HPLC trace of the reaction of peptide **7a** at 220 nm (5-70% MeCN over 21 min).

Synthesis of peptide 8b: Methionine sulfoximidation of peptide 8a

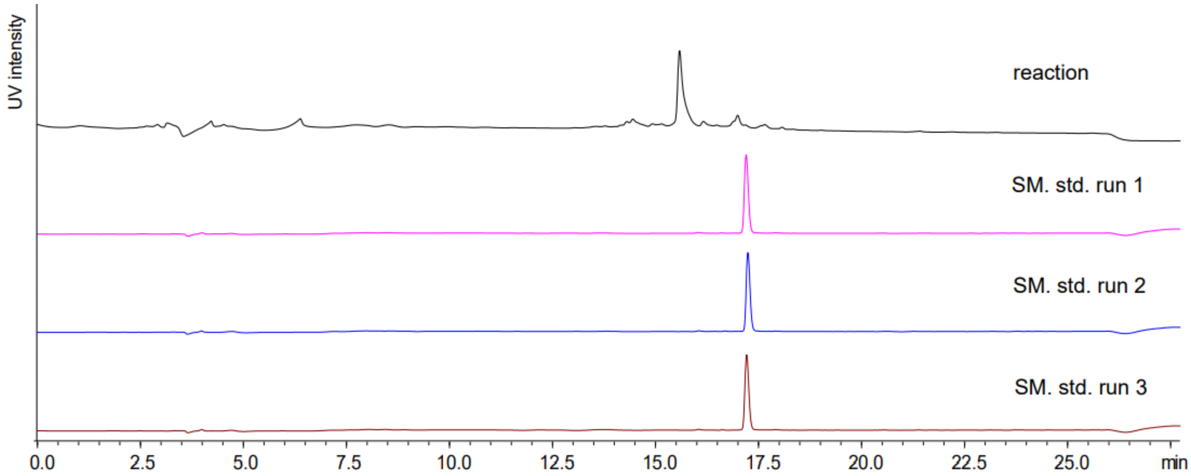

	peak area	average of run 1, 2, 3	yield
reaction (10 mM)	7124456	-	76%
SM std run 1 (10 mM)	9578378	9341974	-
SM std run 2 (10 mM)	9273950		
SM std run 3 (10 mM)	9173593		

Figure S20. RP-HPLC trace of the reaction of peptide **8a** at 220 nm (5-50% MeCN over 21 min).

For Figure 2

Synthesis of peptide 4a: Peptide 4 coupled with boronic acid a

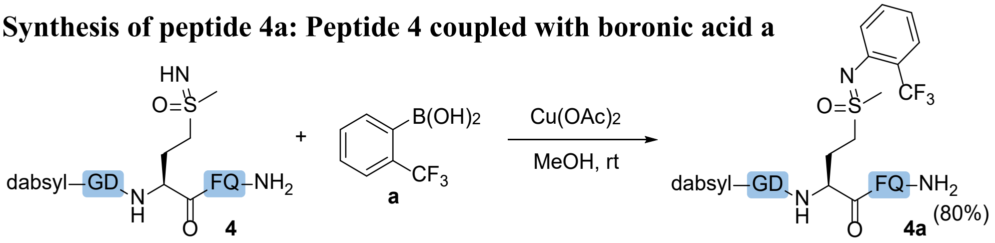

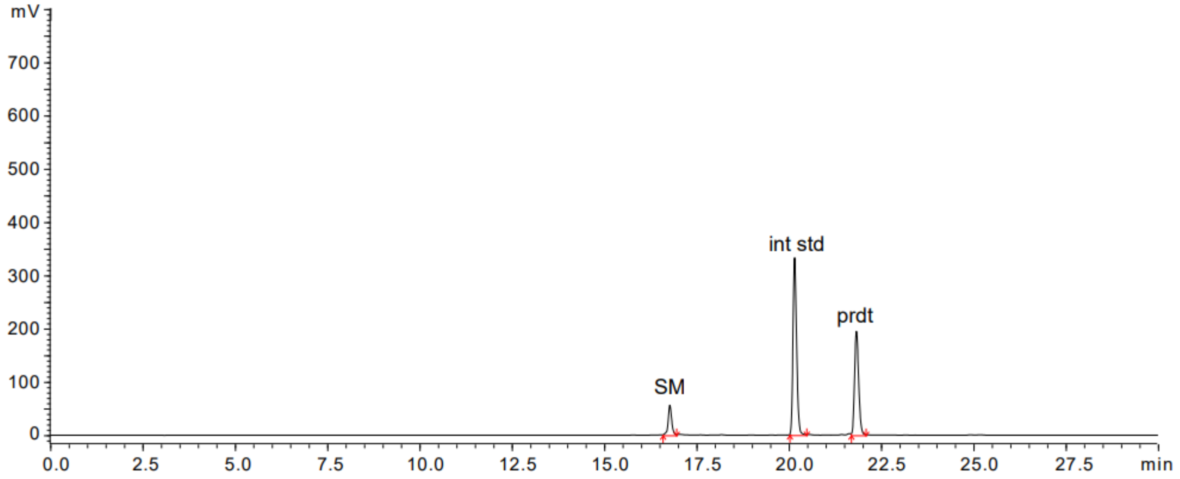

	peak area	yield
product (0.2 mM)	322490	80%
int std (0.2 mM)	404746	-

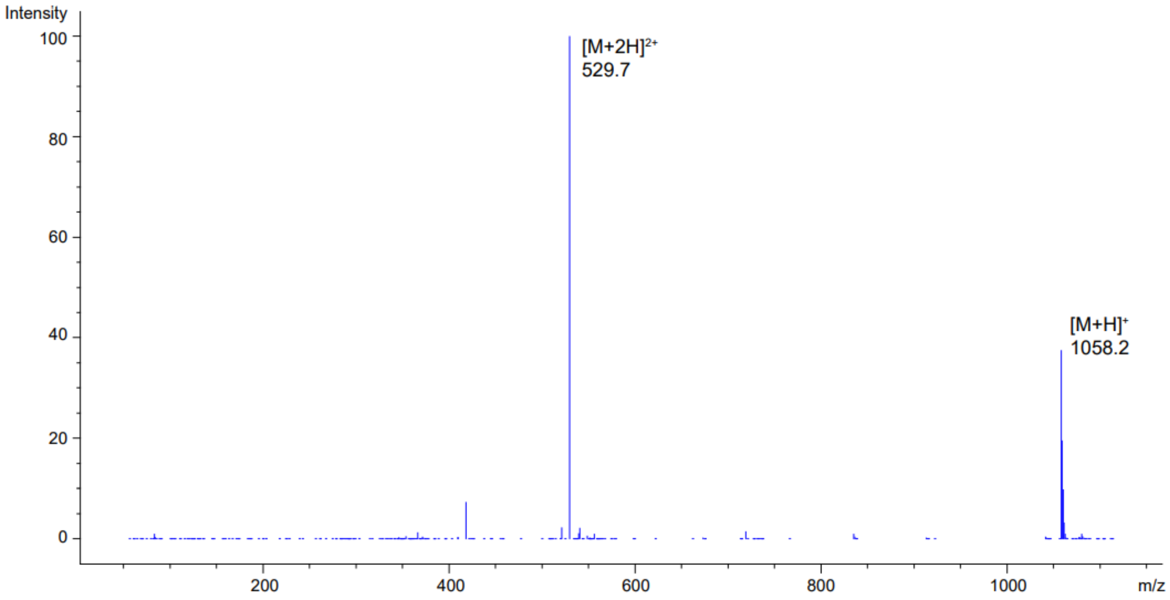

Figure S21. RP-HPLC trace of the reaction of dabsyl-labeled methionine sulfoximine peptide **4** with boronic acid **a** at 500 nm (5-70% MeCN over 21 min) and ESI-MS spectrum of product **4a**. m/z 529.7 and 1058.2 correspond to $[M+2H]^{2+}$ and $[M+H]^{+}$, respectively.

Synthesis of peptide 4b: Peptide 4 coupled with boronic acid b

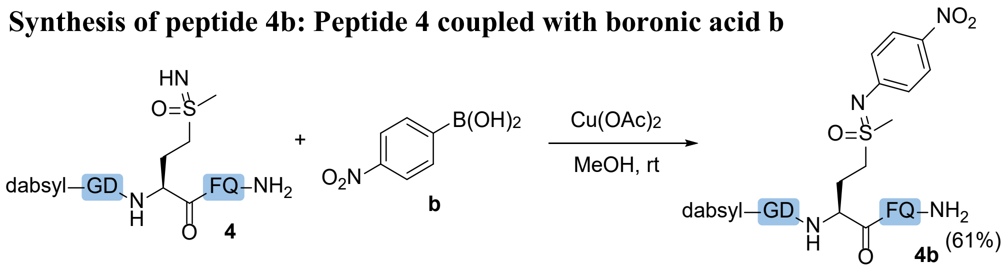

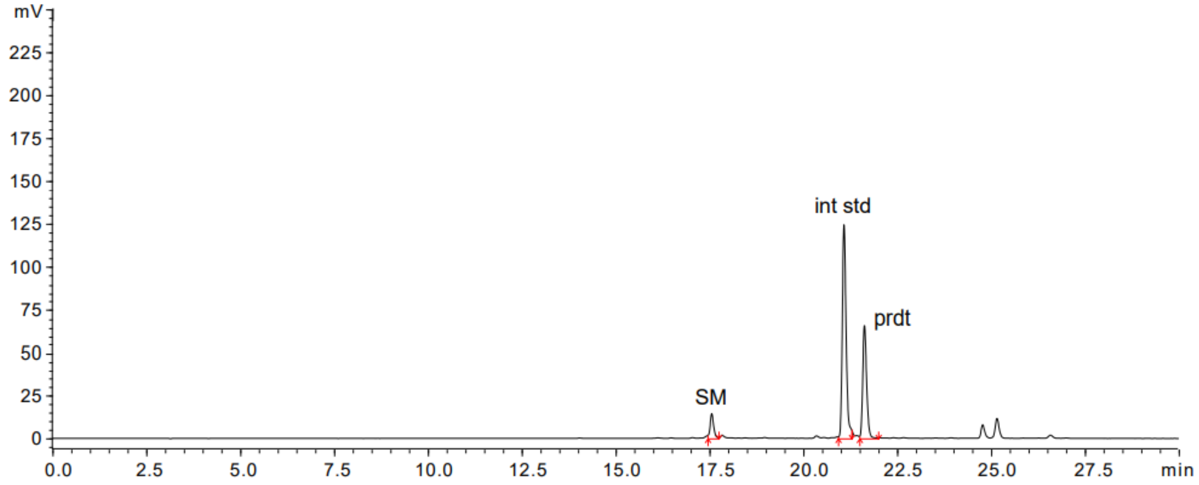

	peak area	yield
product (0.2 mM)	536625	61%
int std (0.2 mM)	882753	-

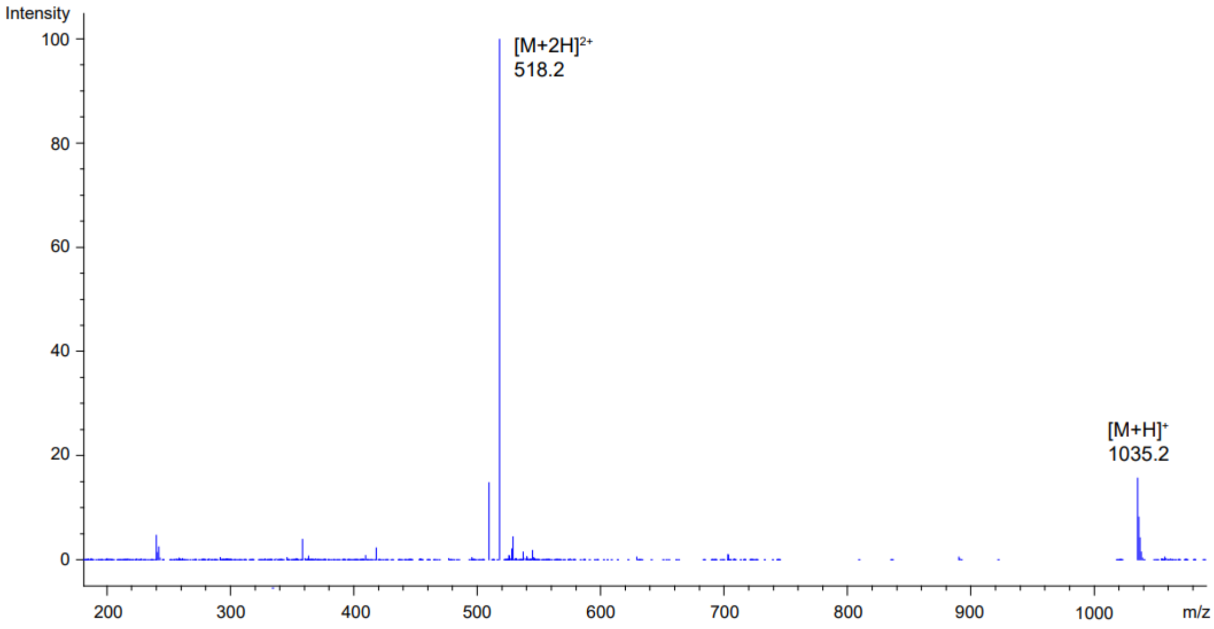

Figure S22. RP-HPLC trace of the reaction of dabsyl-labeled methionine sulfoximine peptide **4** with boronic acid **b** at 500 nm (5-70% MeCN over 21 min) and ESI-MS spectrum of product **4b**. m/z 518.2 and 1035.2 correspond to $[M+2H]^{2+}$ and $[M+H]^{+}$, respectively.

Synthesis of peptide 4c: Peptide 4 coupled with boronic acid c

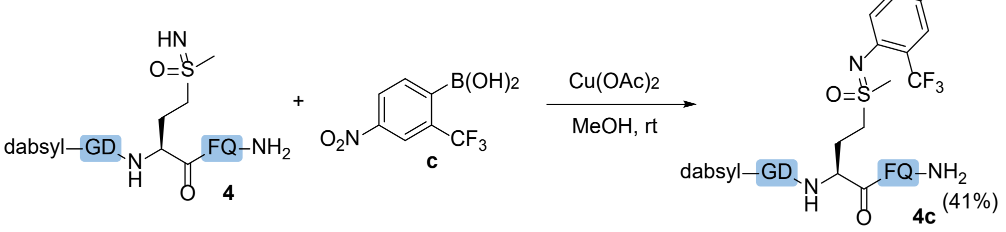

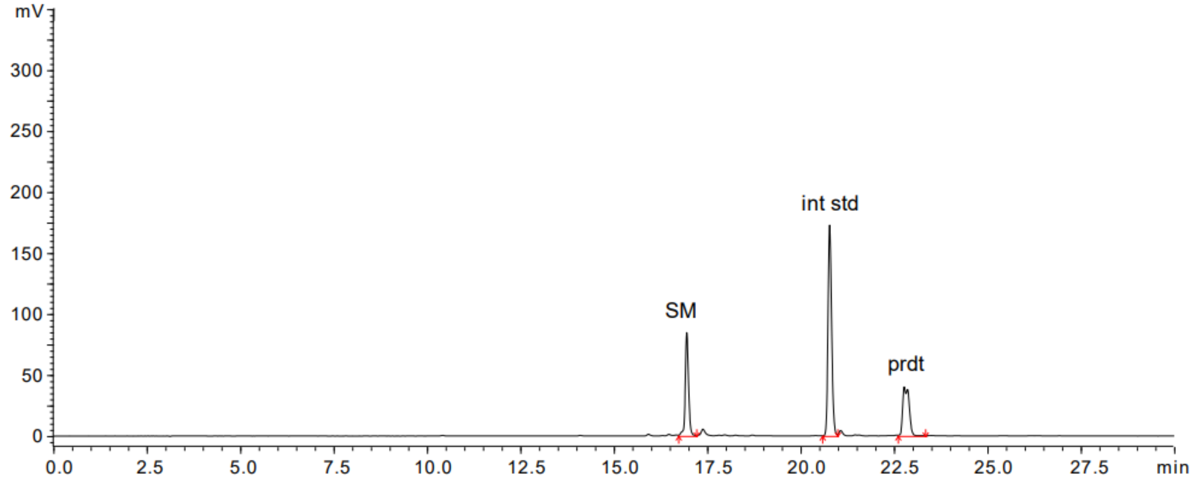

	peak area	yield
product (0.2 mM)	482481	41%
int std (0.2 mM)	1167170	-

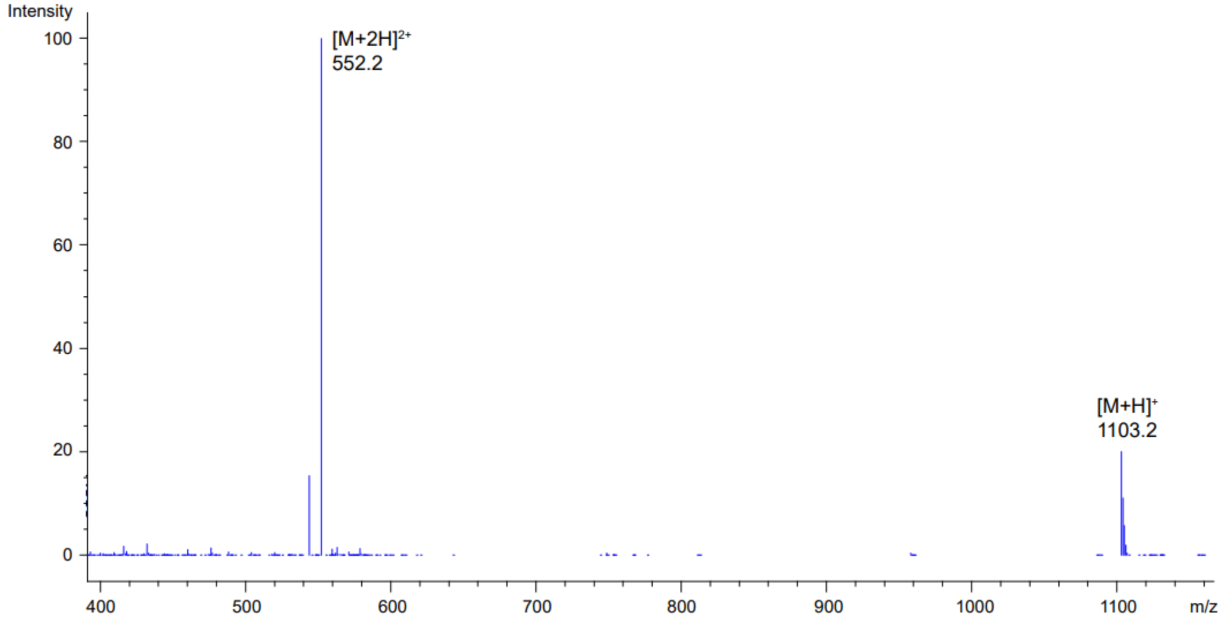

Figure S23. RP-HPLC trace of the reaction of dabsyl-labeled methionine sulfoximine peptide **4** with boronic acid **c** at 500 nm (5-70% MeCN over 21 min) and ESI-MS spectrum of product **4c**. m/z 552.2 and 1103.2 correspond to $[M+2H]^{2+}$ and $[M+H]^{+}$, respectively.

Synthesis of peptide 4d: Peptide 4 coupled with boronic acid d

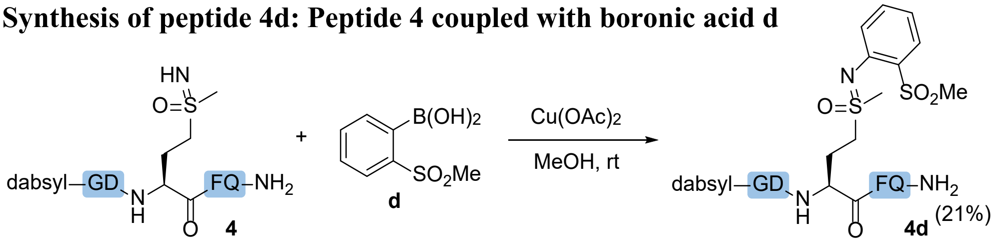

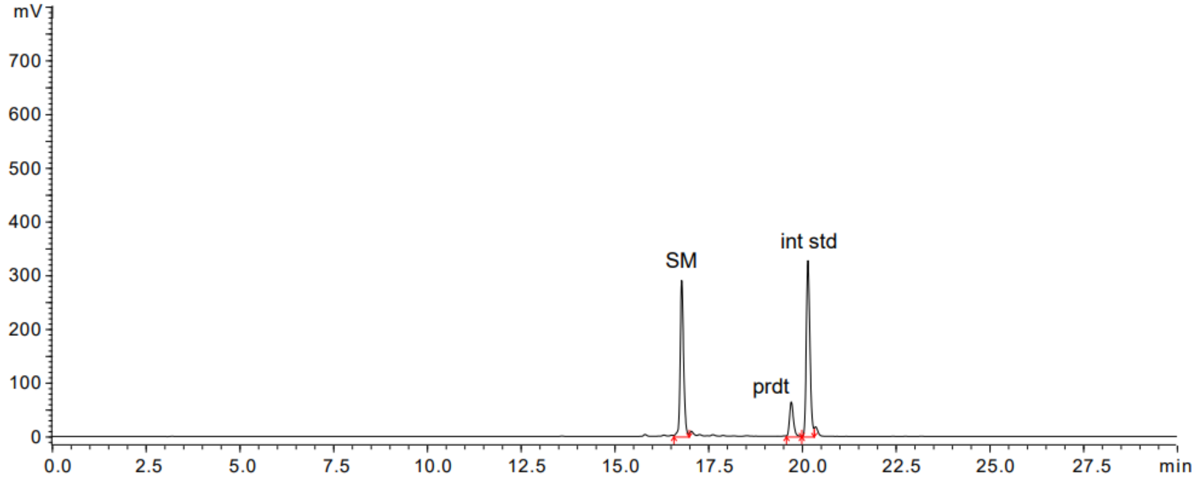

	peak area	yield
product (0.2 mM)	463787	21%
int std (0.2 mM)	2233828	-

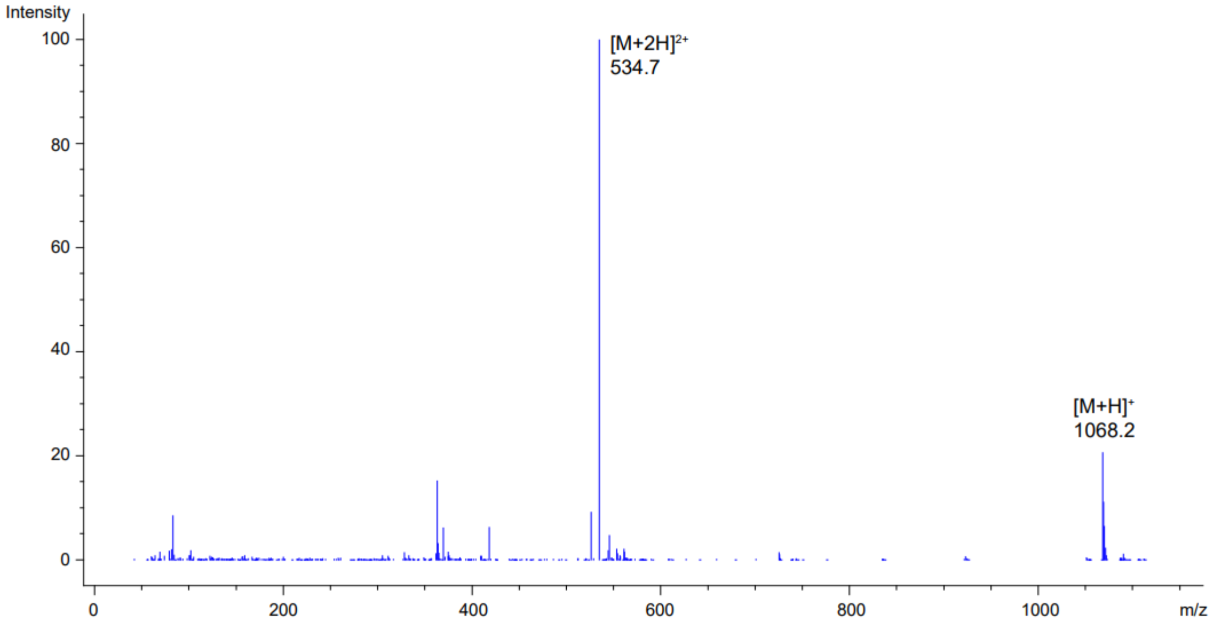

Figure S24. RP-HPLC trace of the reaction of dabsyl-labeled methionine sulfoximine peptide **4** with boronic acid **d** at 500 nm (5-70% MeCN over 21 min) and ESI-MS spectrum of product **4d**. m/z 534.7 and 1068.2 correspond to $[M+2H]^{2+}$ and $[M+H]^{+}$, respectively.

Synthesis of peptide 4e: Peptide 4 coupled with boronic acid e

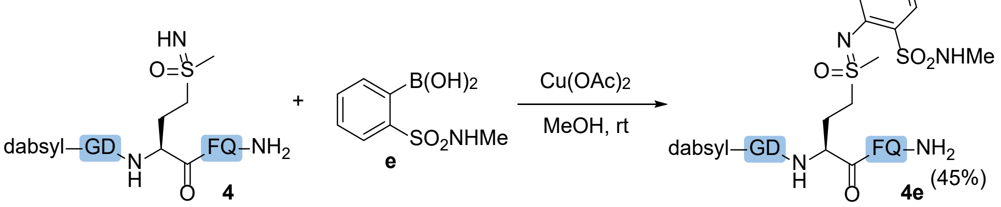

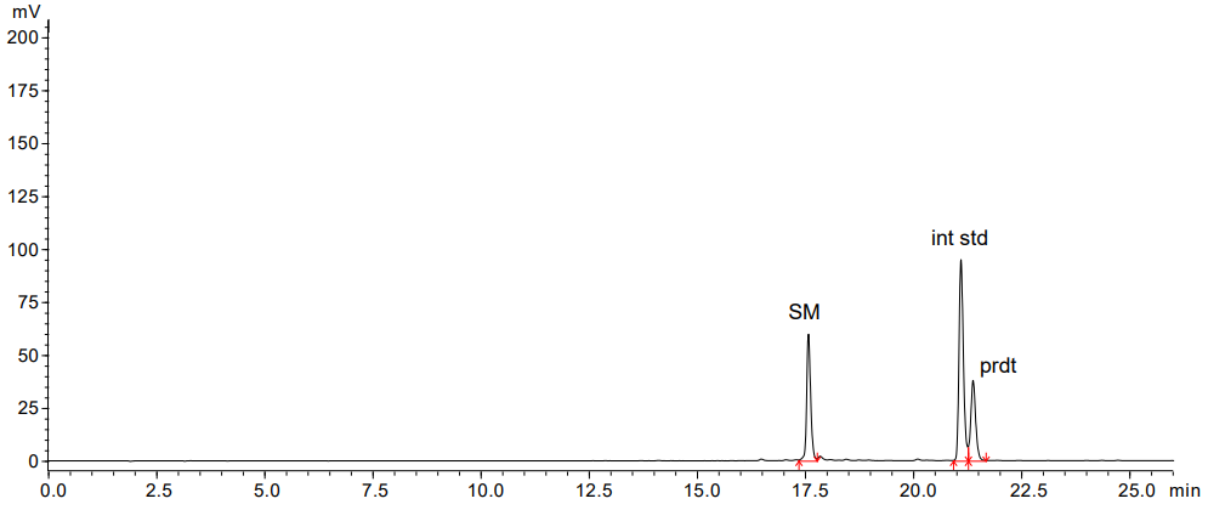

	peak area	yield
product (0.2 mM)	301038	45%
int std (0.2 mM)	672326	-

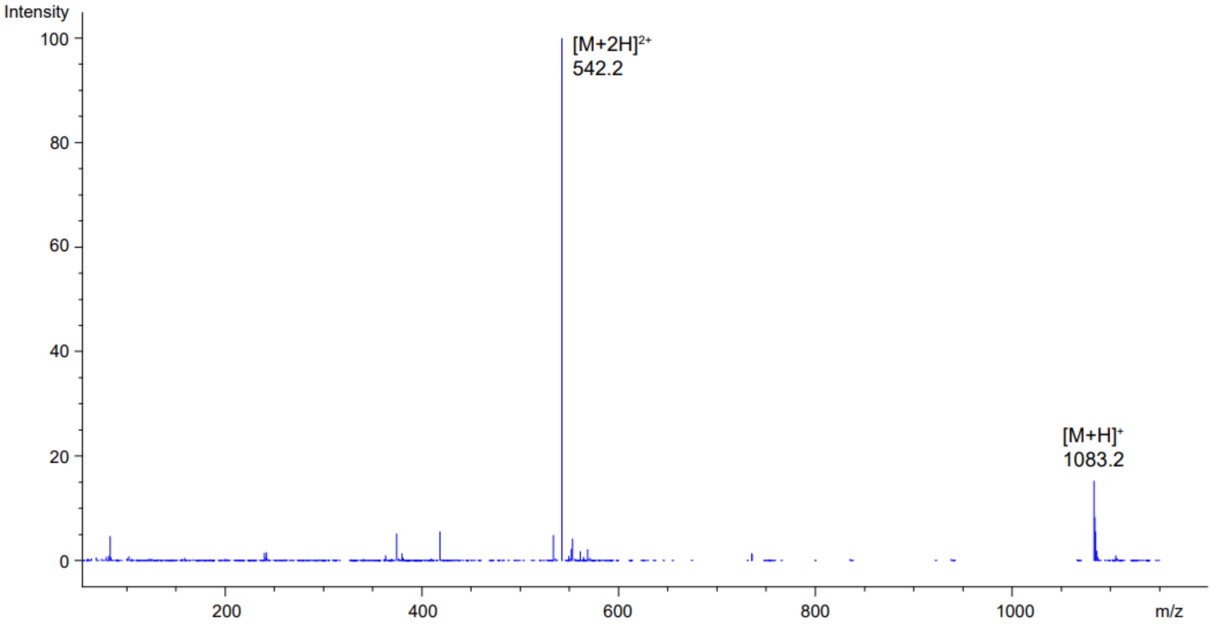

Figure S25. RP-HPLC trace of the reaction of dabsyl-labeled methionine sulfoximine peptide **4** with boronic acid **e** at 500 nm (5-65% MeCN over 21 min) and ESI-MS spectrum of product **4e**. m/z 542.2 and 1083.2 correspond to $[M+2H]^{2+}$ and $[M+H]^{+}$, respectively.

Synthesis of peptide 4f: Peptide 4 coupled with boronic acid f

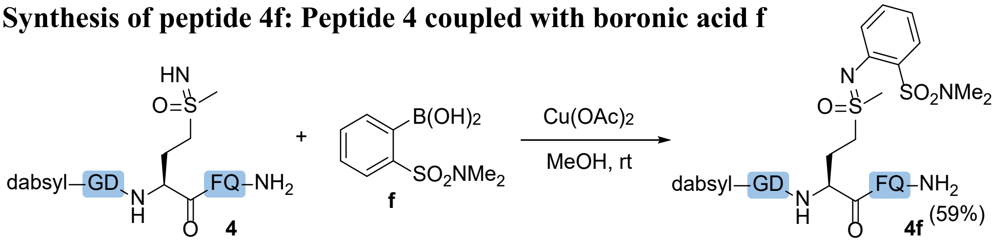

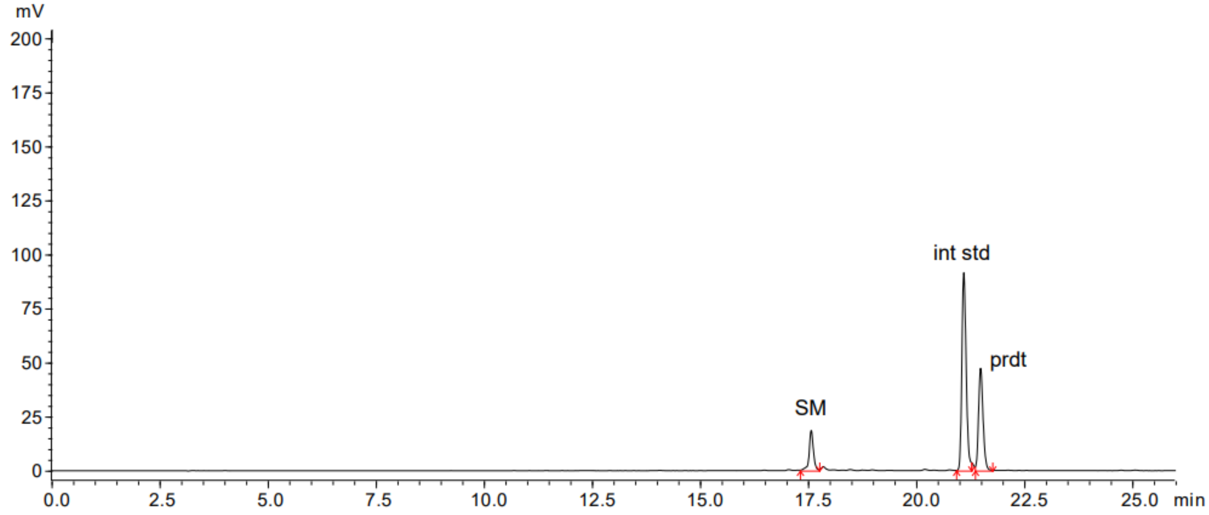

	peak area	yield
product (0.2 mM)	376903	59%
int std (0.2 mM)	639591	-

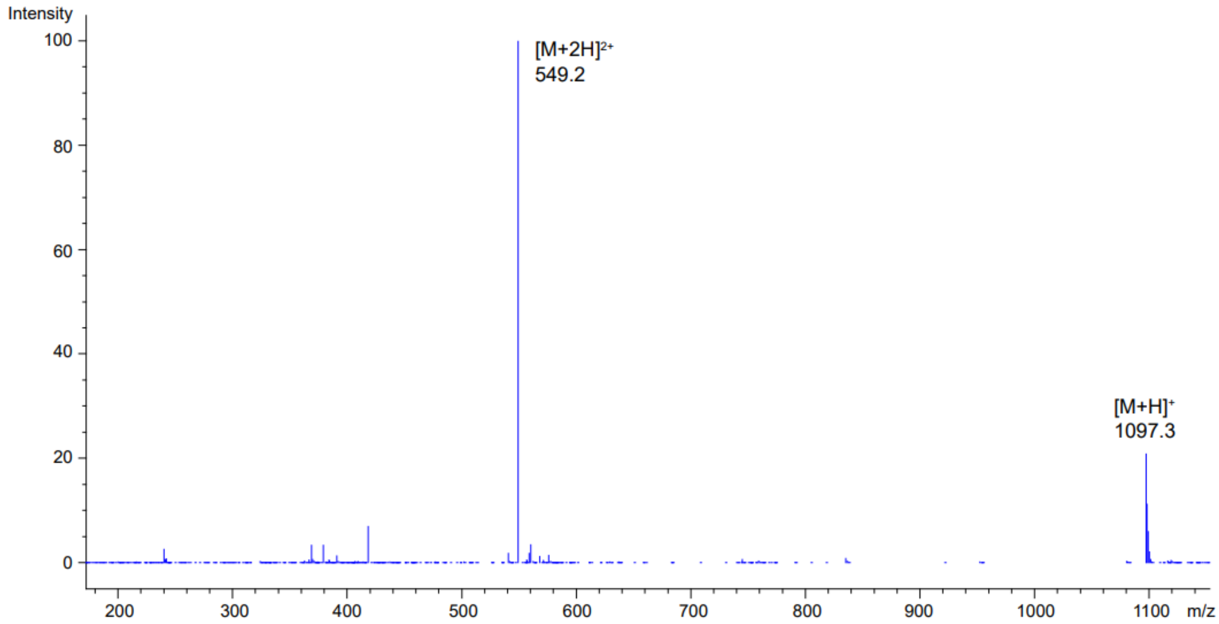

Figure S26. RP-HPLC trace of the reaction of dabsyl-labeled methionine sulfoximine peptide **4** with boronic acid **f** at 500 nm (5-65% MeCN over 21 min) and ESI-MS spectrum of product **4f**. m/z 549.2 and 1097.3 correspond to $[M+2H]^{2+}$ and $[M+H]^{+}$, respectively.

Synthesis of peptide 4g: Peptide 4 coupled with boronic acid g

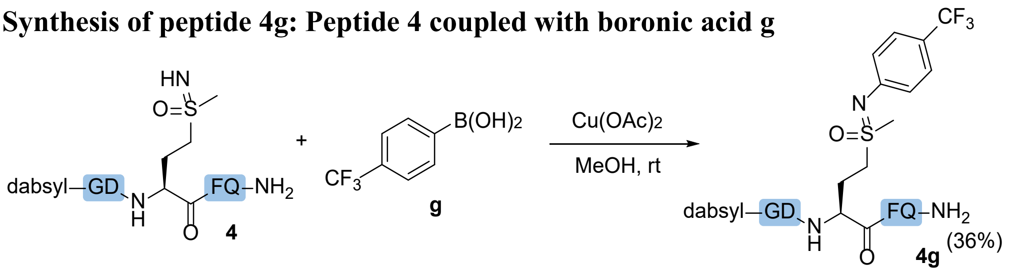

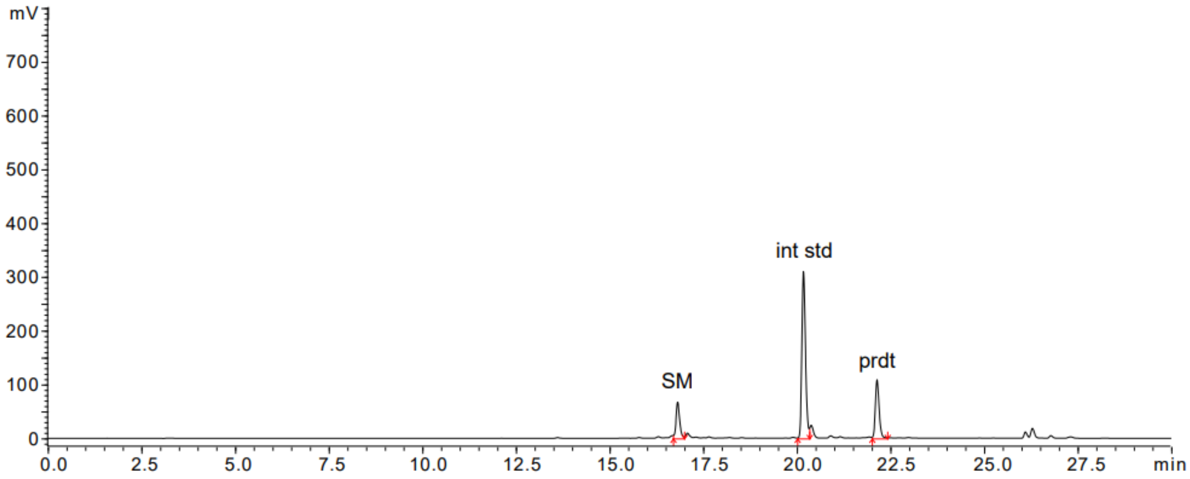

	peak area	yield
product (0.2 mM)	765649	36%
int std (0.2 mM)	2124652	-

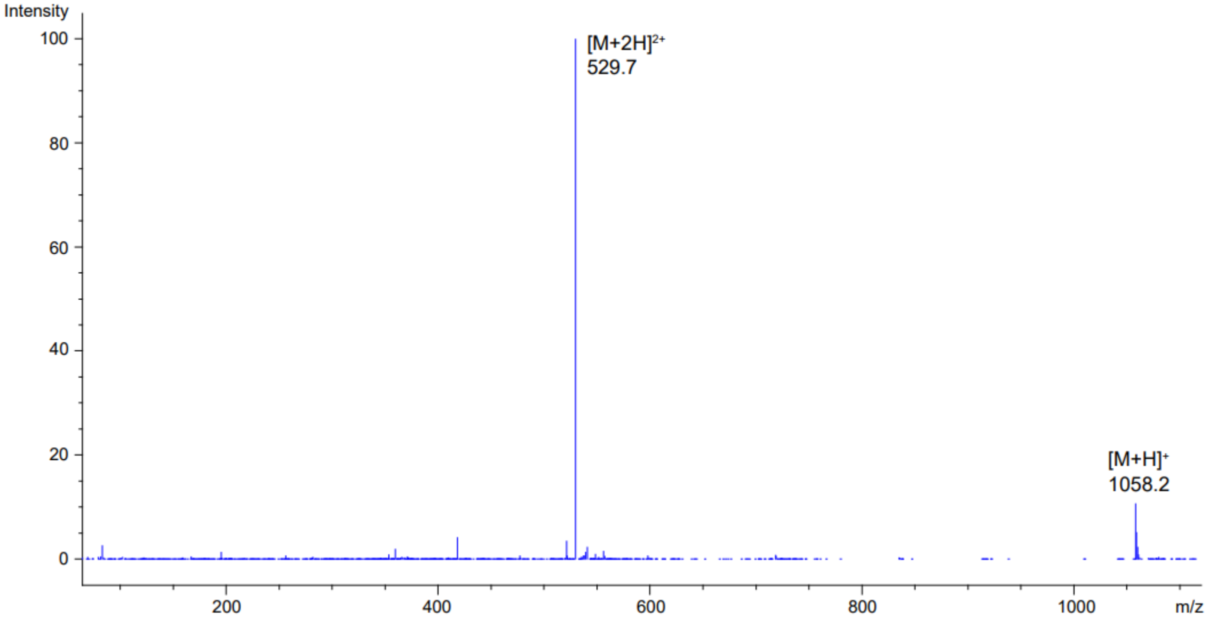

Figure S27. RP-HPLC trace of the reaction of dabsyl-labeled methionine sulfoximine peptide **4** with boronic acid **g** at 500 nm (5-70% MeCN over 21 min) and ESI-MS spectrum of product **4g**. m/z 529.7 and 1058.2 correspond to $[M+2H]^{2+}$ and $[M+H]^{+}$, respectively.

Synthesis of peptide 4h: Peptide 4 coupled with boronic acid h

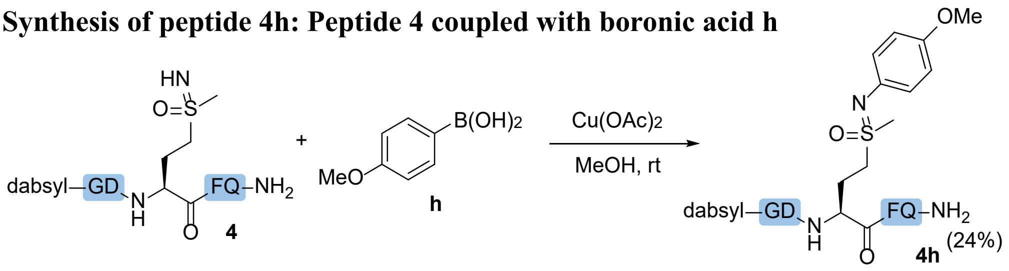

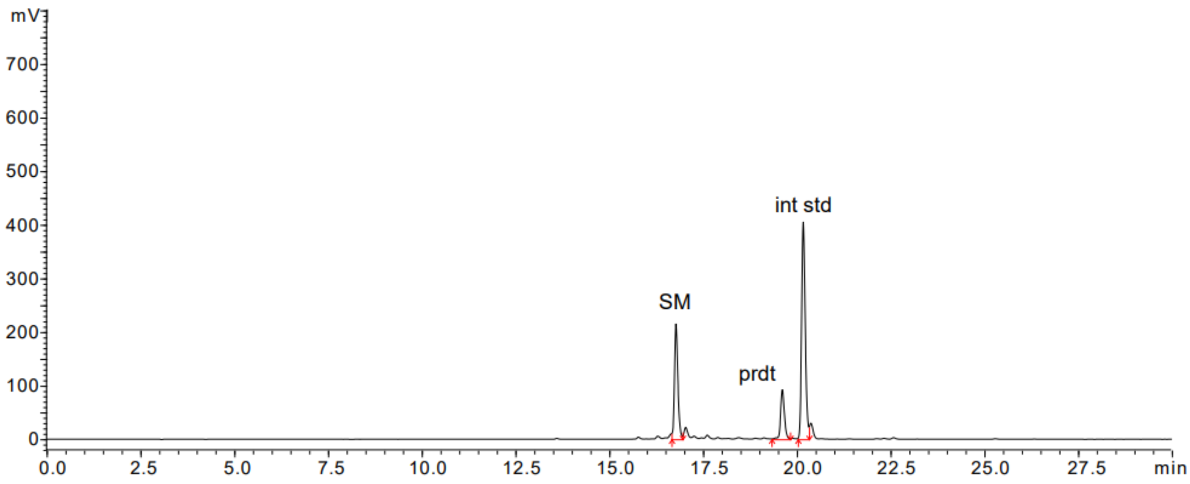

	peak area	yield
product (0.2 mM)	660861	24%
int std (0.2 mM)	2779907	-

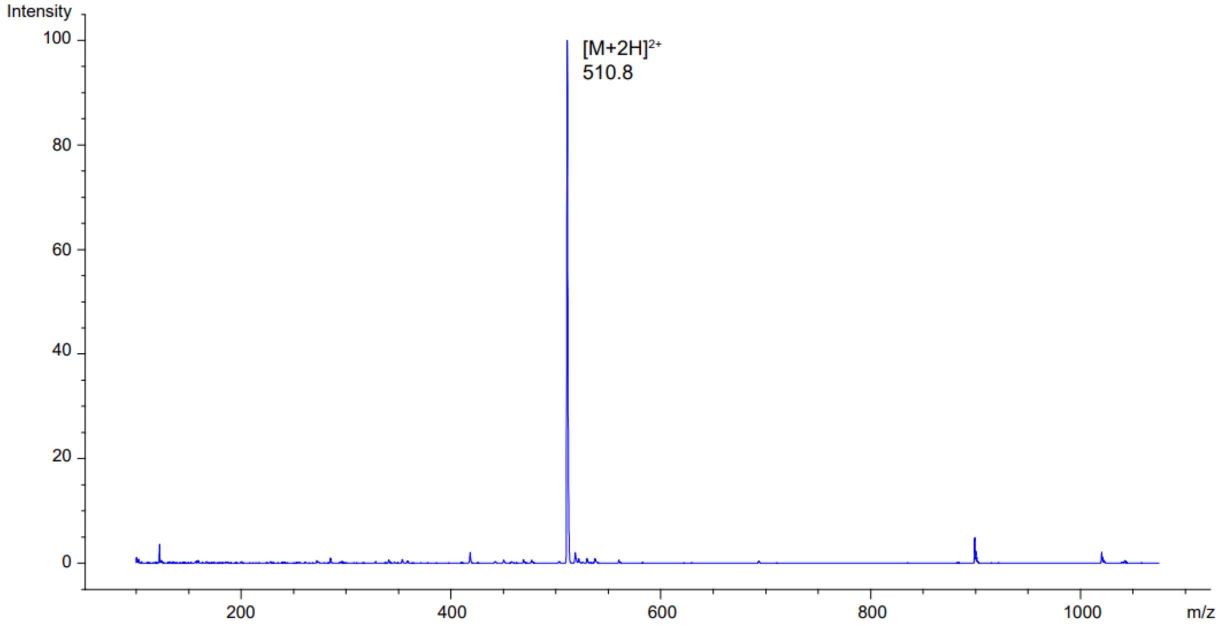

Figure S28. RP-HPLC trace of the reaction of dabsyl-labeled methionine sulfoximine peptide **4** with boronic acid **h** at 500 nm (5-70% MeCN over 21 min) and ESI-MS spectrum of product **4h**. m/z 510.8 correspond to $[M+2H]^{2+}$.

Synthesis of peptide 4i: Peptide 4 coupled with boronic acid i

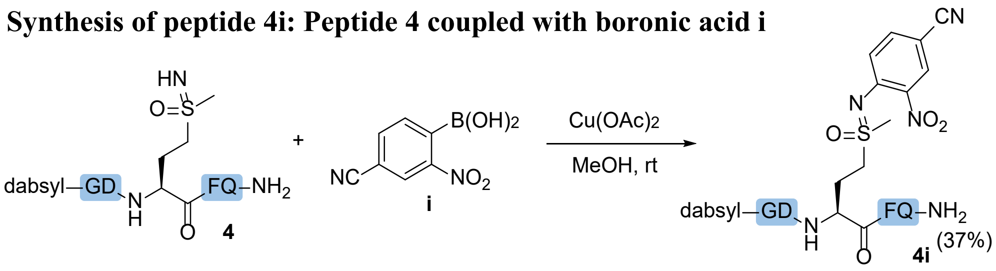

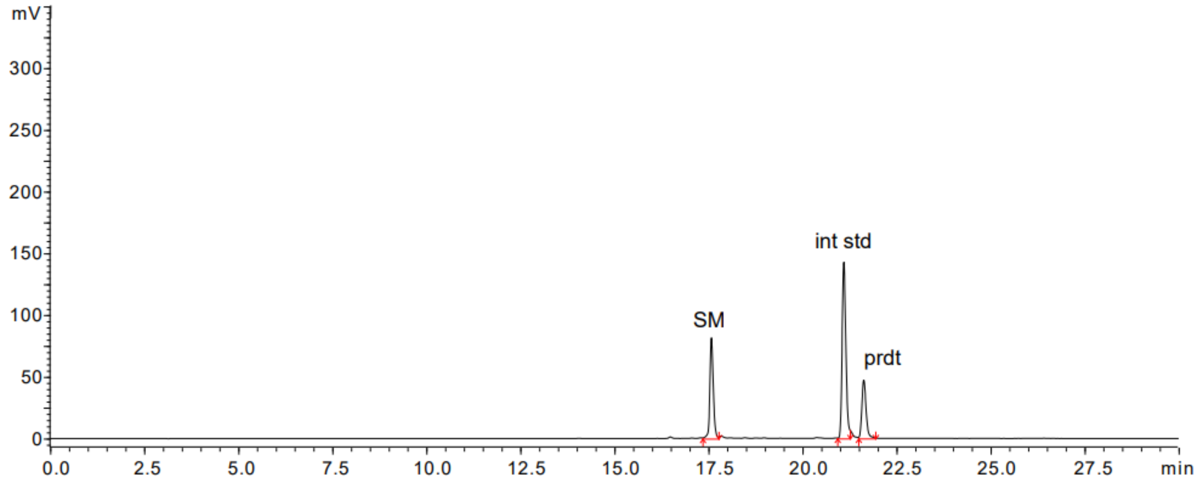

	peak area	yield
product (0.2 mM)	368091	37%
int std (0.2 mM)	991626	-

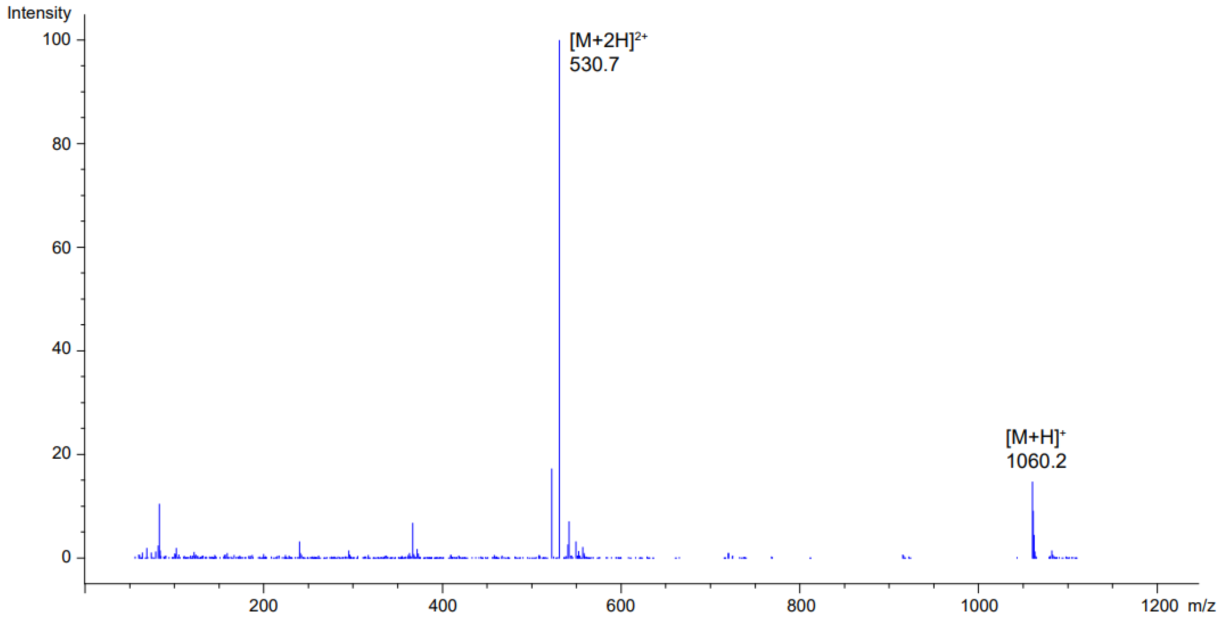

Figure S29. RP-HPLC trace of the reaction of dabsyl-labeled methionine sulfoximine peptide **4** with boronic acid **i** at 500 nm (5-65% MeCN over 21 min) and ESI-MS spectrum of product **4i**. m/z 530.7 and 1060.2 correspond to $[M+2H]^{2+}$ and $[M+H]^{+}$, respectively.

Synthesis of peptide 4j: Peptide 4 coupled with boronic acid j

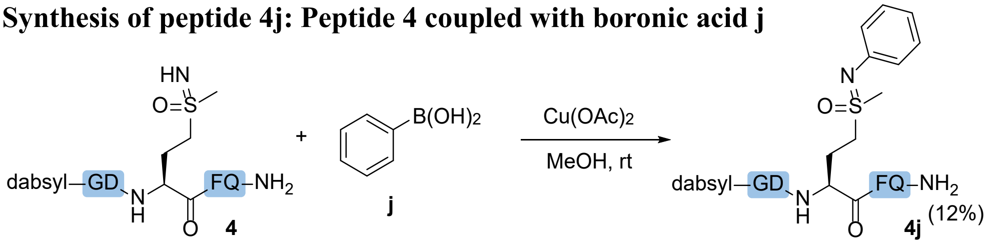

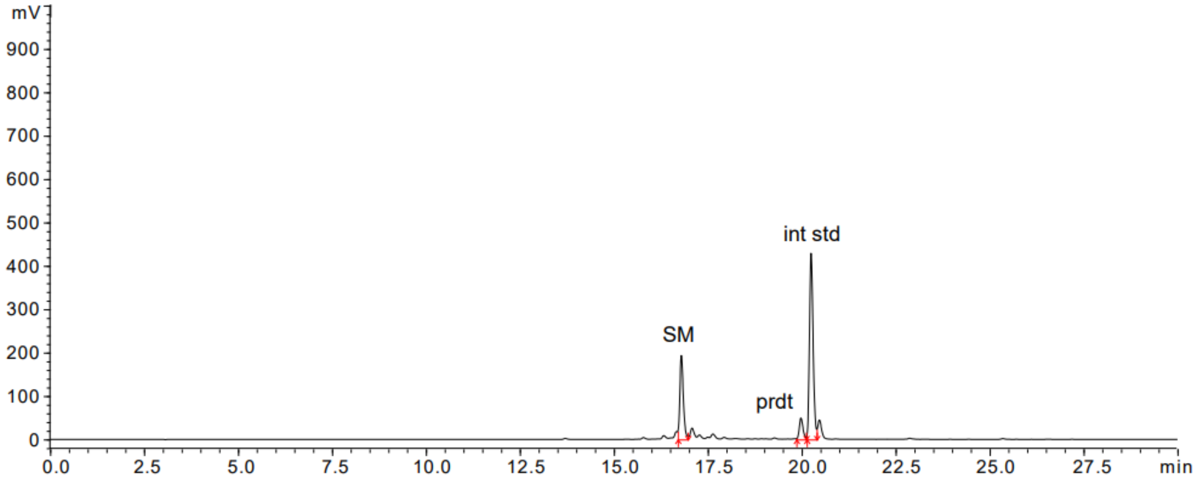

	peak area	yield
product (0.2 mM)	336608	12%
int std (0.2 mM)	2916990	-

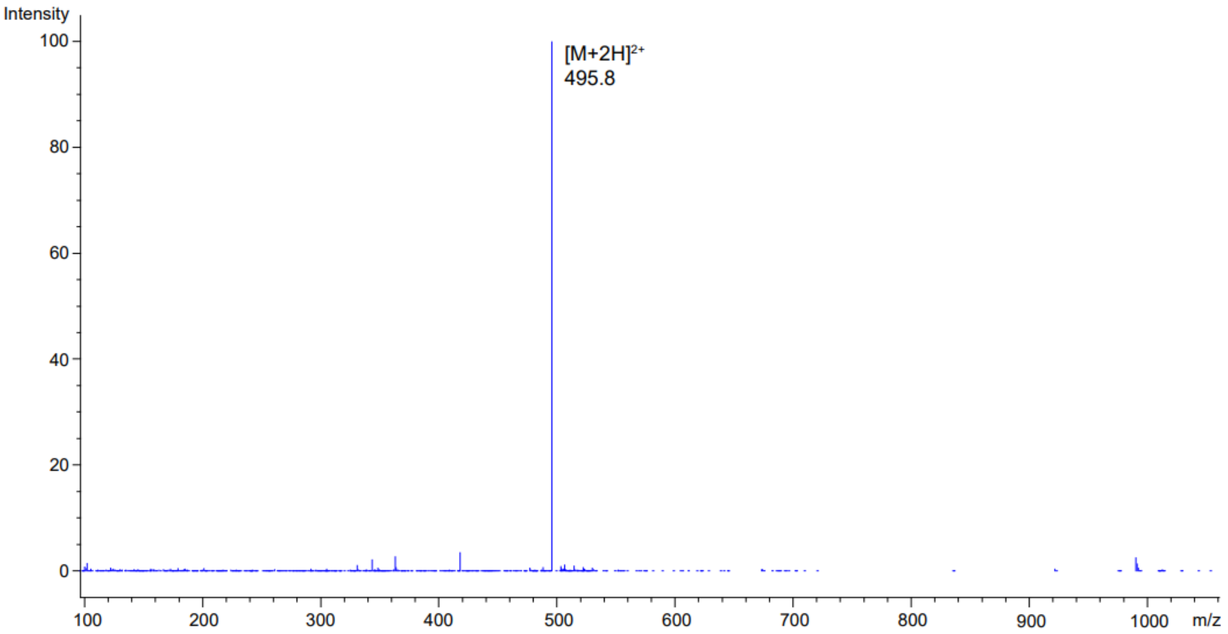

Figure S30. RP-HPLC trace of the reaction of dabsyl-labeled methionine sulfoximine peptide **4** with boronic acid **j** at 500 nm (5-70% MeCN over 21 min) and ESI-MS spectrum of product **4j**. m/z 495.8 correspond to $[M+2H]^{2+}$.

Synthesis of peptide 4k: Peptide 4 coupled with boronic acid k

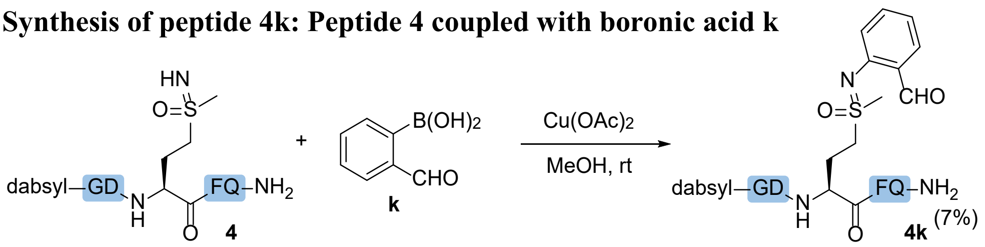

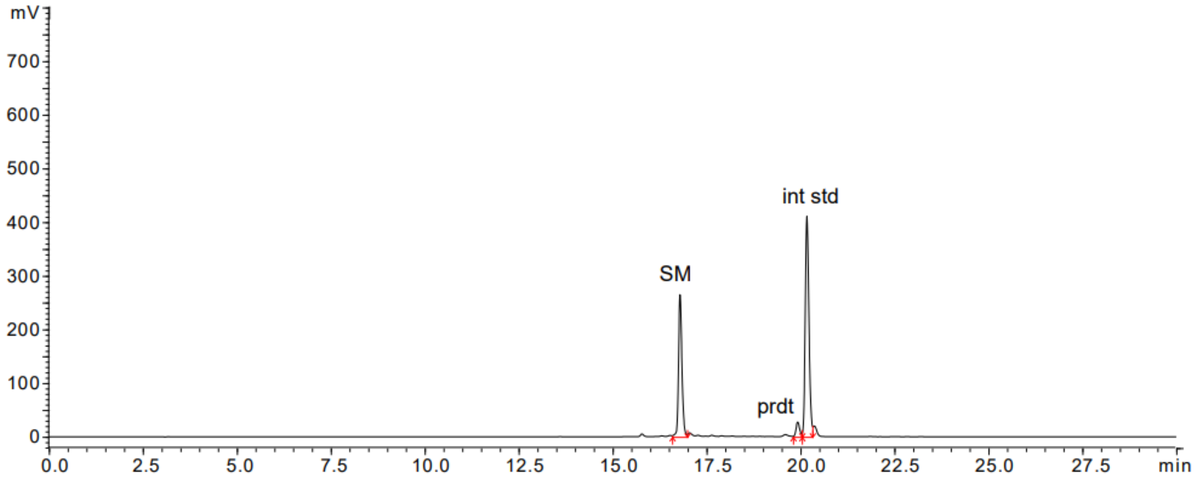

	peak area	yield
product (0.2 mM)	183965	7%
int std (0.2 mM)	2829672	-

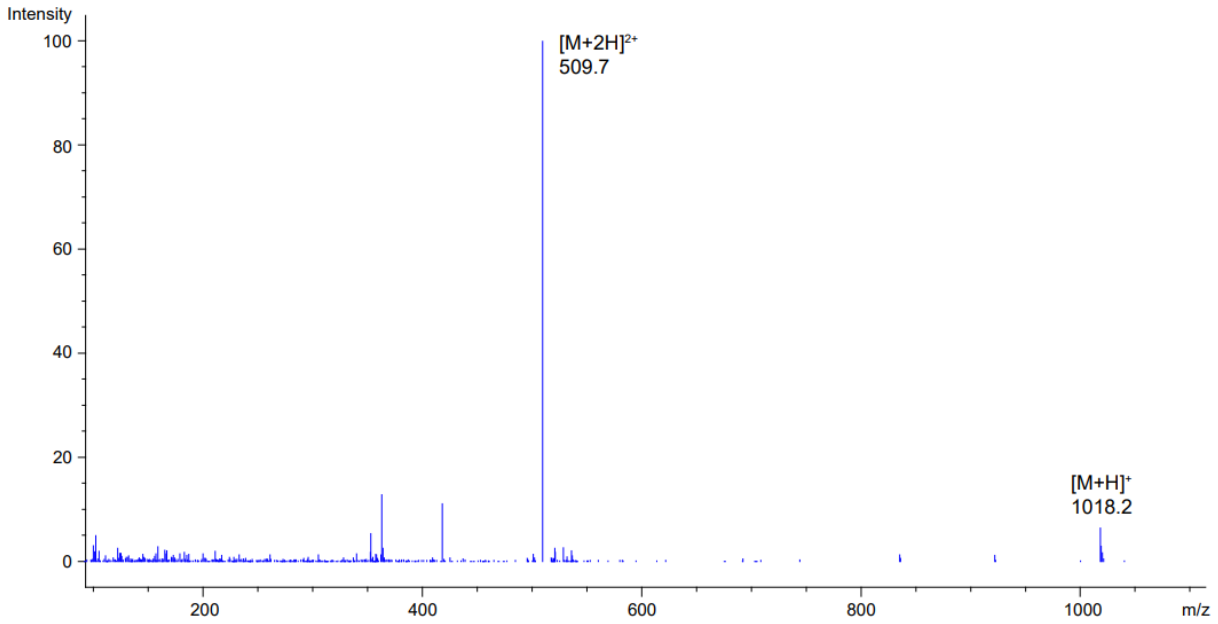

Figure S31. RP-HPLC trace of the reaction of dabsyl-labeled methionine sulfoximine peptide **4** with boronic acid **k** at 500 nm (5-70% MeCN over 21 min) and ESI-MS spectrum of product **4k**. m/z 509.7 and 1018.2 correspond to $[M+2H]^{2+}$ and $[M+H]^{+}$, respectively.

Synthesis of peptide 4l: Peptide 4 coupled with boronic acid l

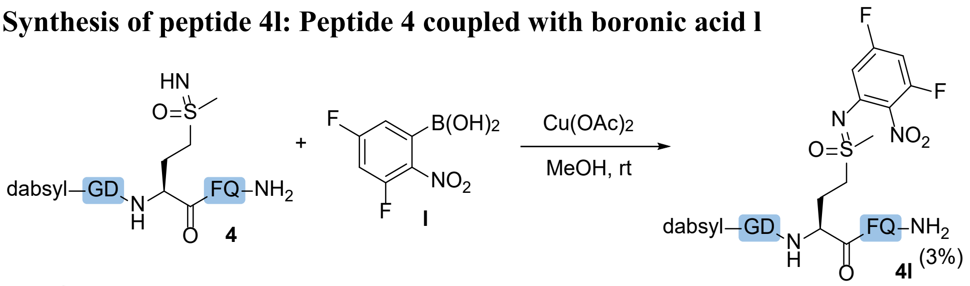

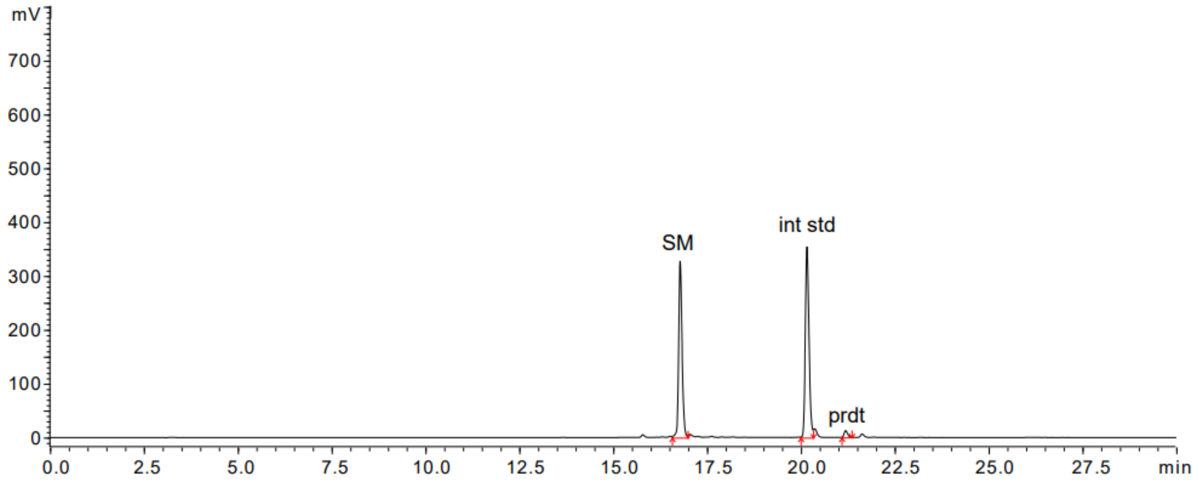

	peak area	yield
product (0.2 mM)	80026	3%
int std (0.2 mM)	2437335	-

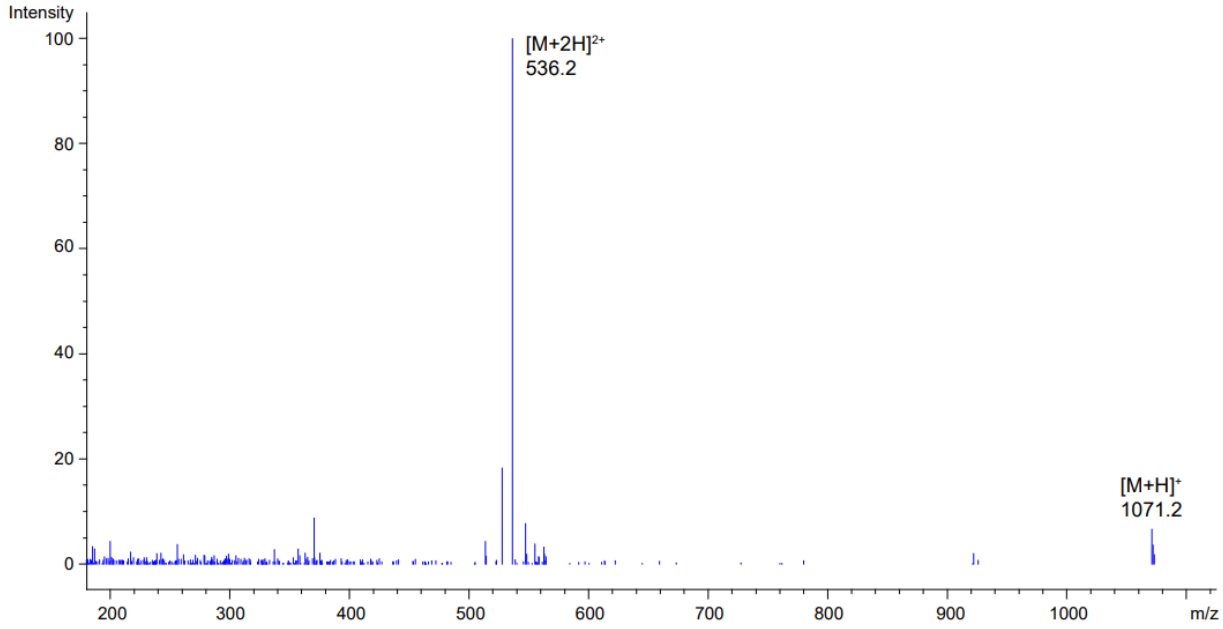

Figure S32. RP-HPLC trace of the reaction of dabsyl-labeled methionine sulfoximine peptide **4** with boronic acid **l** at 500 nm (5-70% MeCN over 21 min) and ESI-MS spectrum of product **4l**. m/z 536.2 and 1071.2 correspond to $[M+2H]^{2+}$ and $[M+H]^{+}$, respectively.

Synthesis of peptide 4m: Peptide 4 coupled with boronic acid m

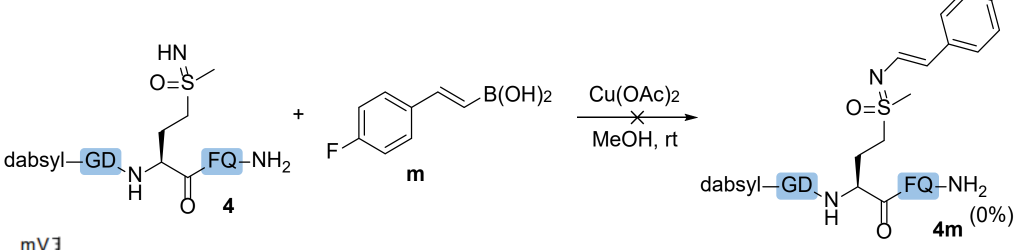

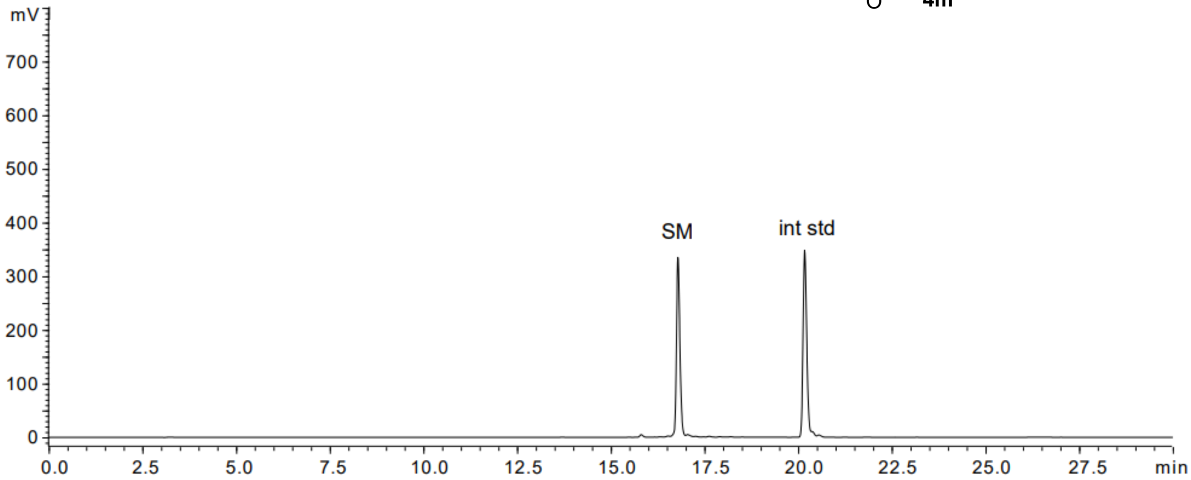

Figure S33. RP-HPLC trace of the reaction of dabsyl-labeled methionine sulfoximine peptide **4** with boronic acid **m** at 500 nm (5-70% MeCN over 21 min). No product was detected.

Synthesis of peptide 4n: Peptide 4 coupled with boronic acid n

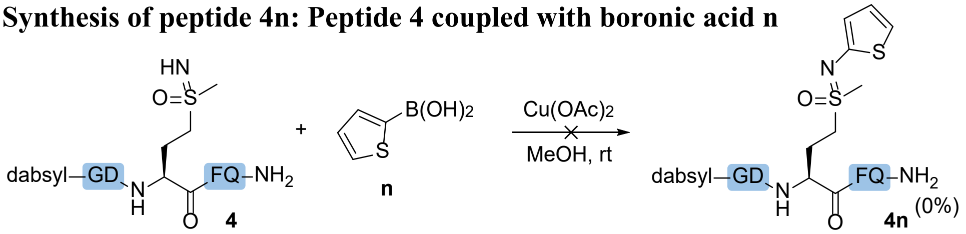

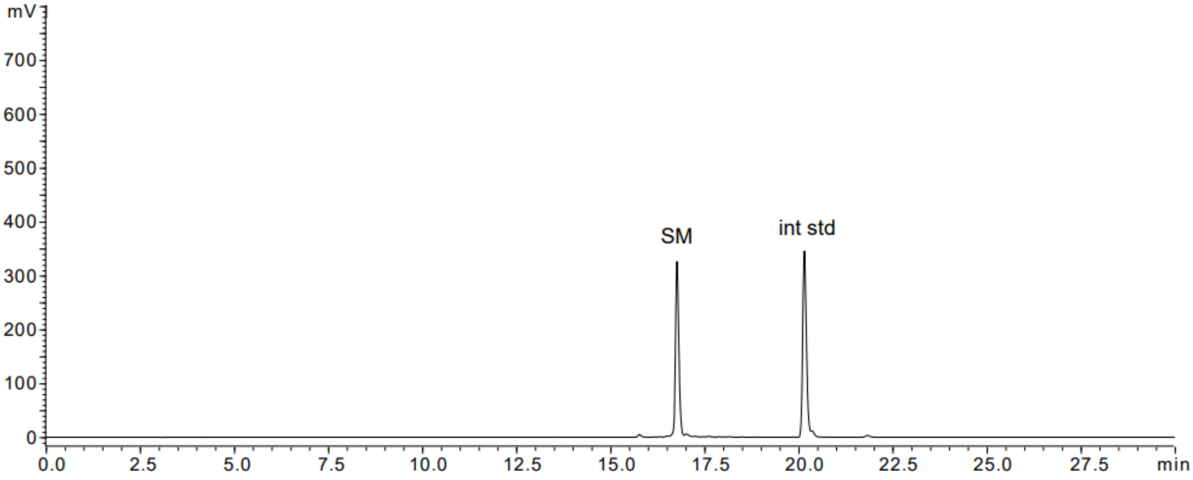

Figure S34. RP-HPLC trace of the reaction of dabsyl-labeled methionine sulfoximine peptide **4** with boronic acid **n** at 500 nm (5-70% MeCN over 21 min). No product was detected.

Synthesis of peptide 4o: Peptide 4 coupled with boronic acid o

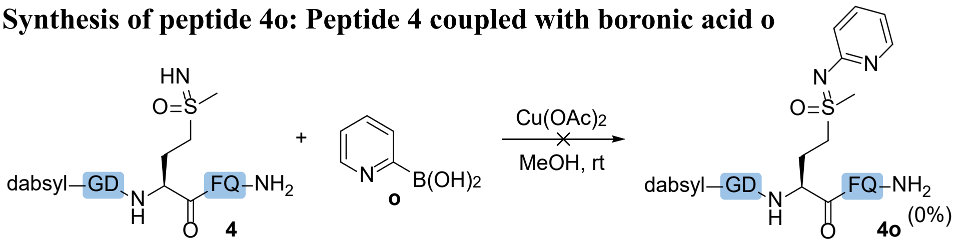

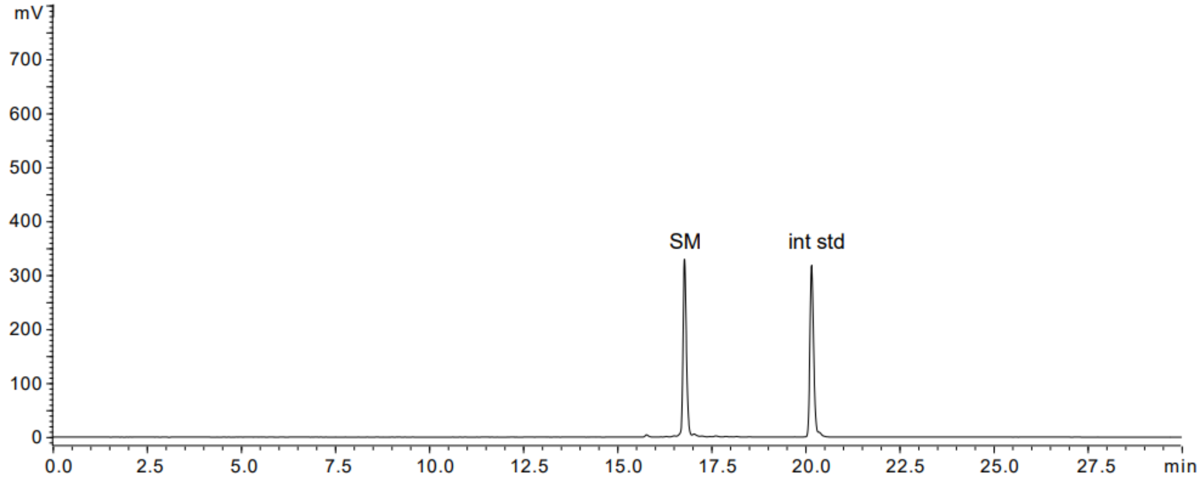

Figure S35. RP-HPLC trace of the reaction of dabsyl-labeled methionine sulfoximine peptide **4** with boronic acid **o** at 500 nm (5-70% MeCN over 21 min). No product was detected.

Synthesis of peptide 4p: Peptide 4 coupled with boronic acid p

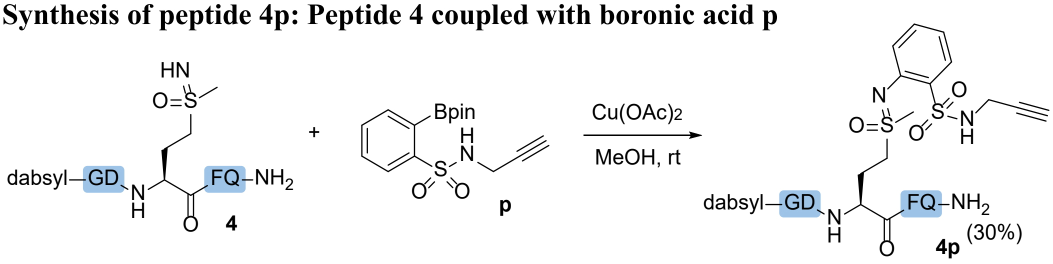

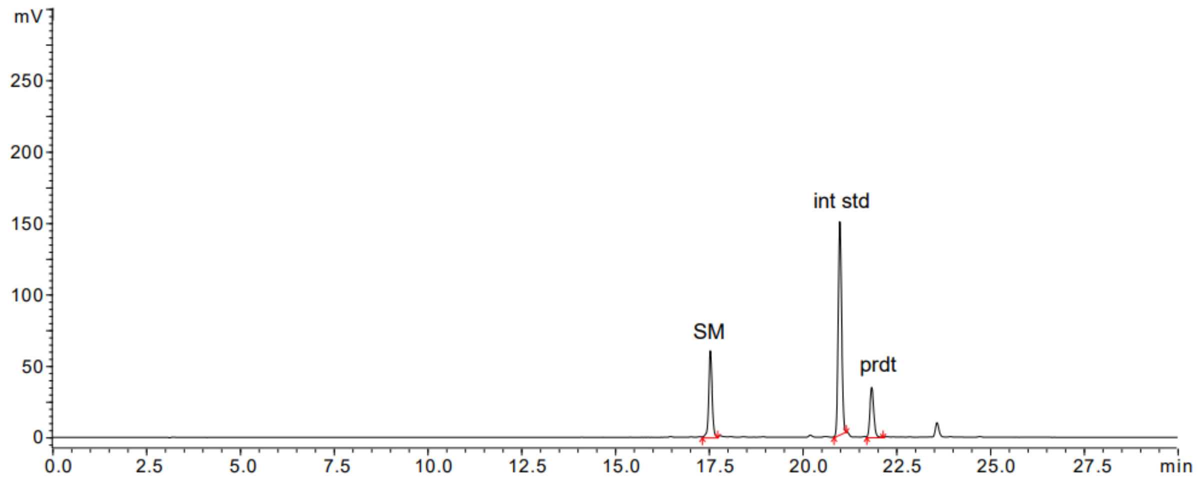

	peak area	yield
product (0.2 mM)	285435	30%
int std (0.2 mM)	950465	-

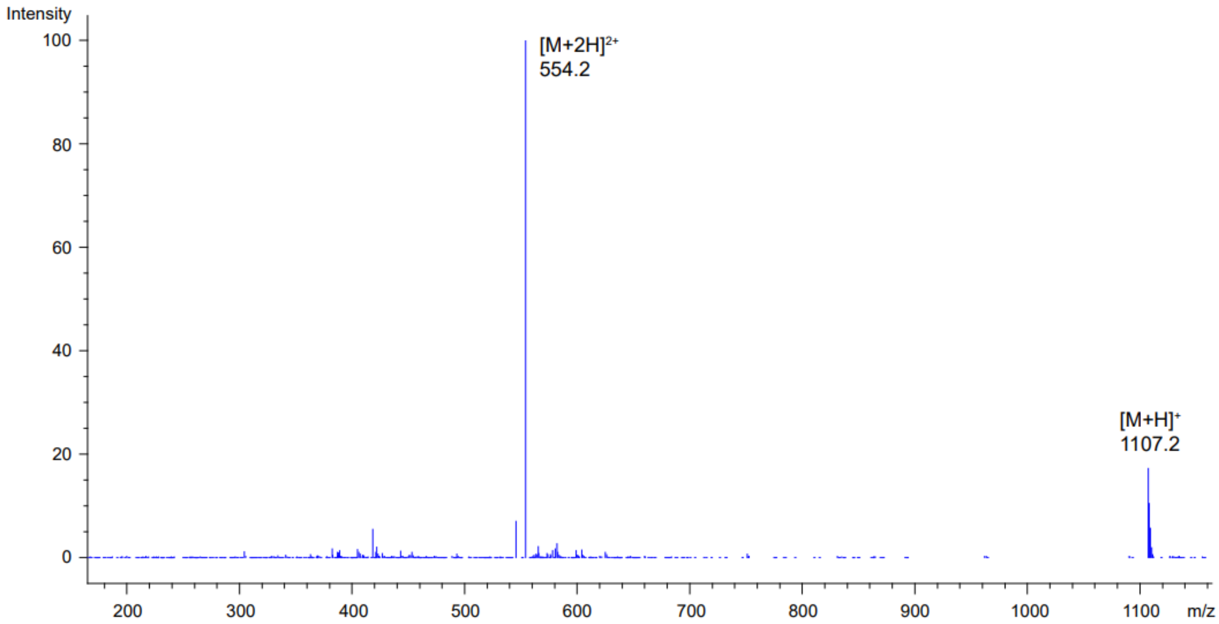

Figure S36. RP-HPLC trace of the reaction of dabsyl-labeled methionine sulfoximine peptide **4** with boronic acid **p** at 500 nm (5-65% MeCN over 21 min) and ESI-MS spectrum of product **4p**. m/z 554.2 and 1107.2 correspond to $[M+2H]^{2+}$ and $[M+H]^{+}$, respectively.

Synthesis of peptide 4q: Peptide 4 coupled with boronic acid q

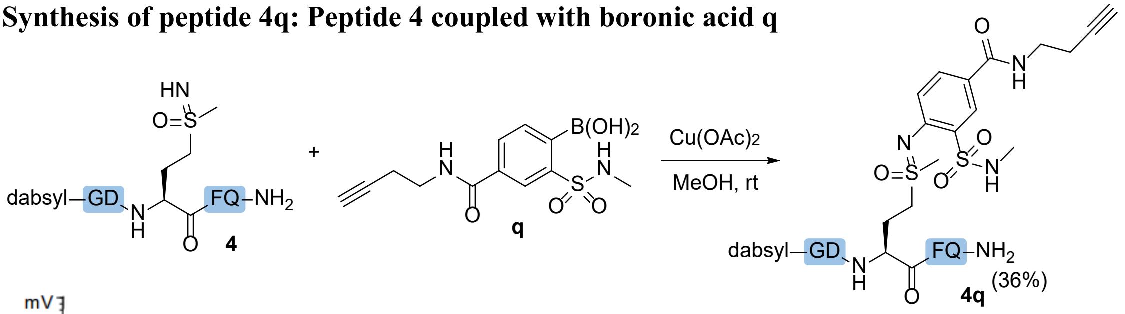

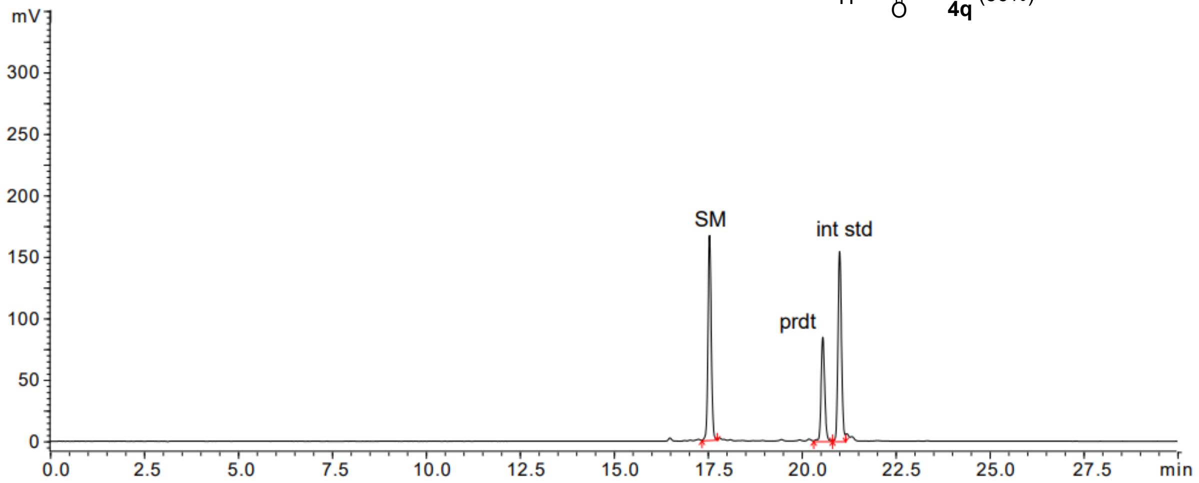

	peak area	yield
product (0.2 mM)	728664	36%
int std (0.1 mM)	1000360	-

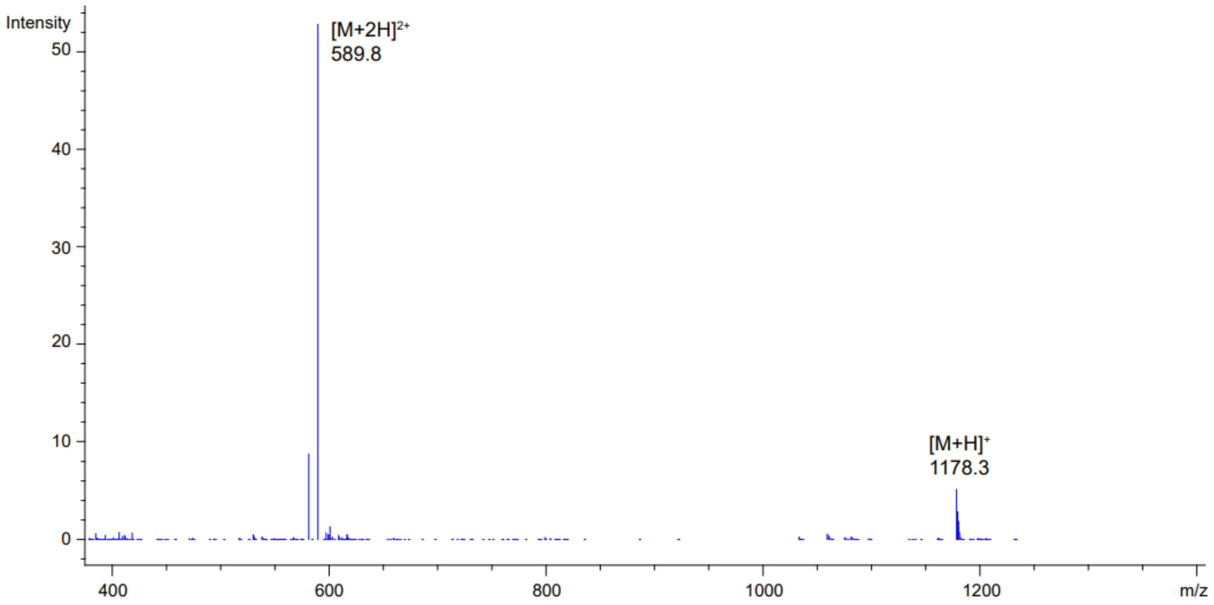

Figure S37. RP-HPLC trace of the reaction of dabsyl-labeled methionine sulfoximine peptide **4** with boronic acid **q** at 500 nm (5-65% MeCN over 21 min) and ESI-MS spectrum of product **4q**. m/z 589.8 and 1178.3 correspond to $[M+2H]^{2+}$ and $[M+H]^{+}$, respectively.

For Table 2

Synthesis of *N*-arylated peptide 1c: N-H coupling of methionine sulfoximine peptide 1b with 4-nitrophenylboronic acid

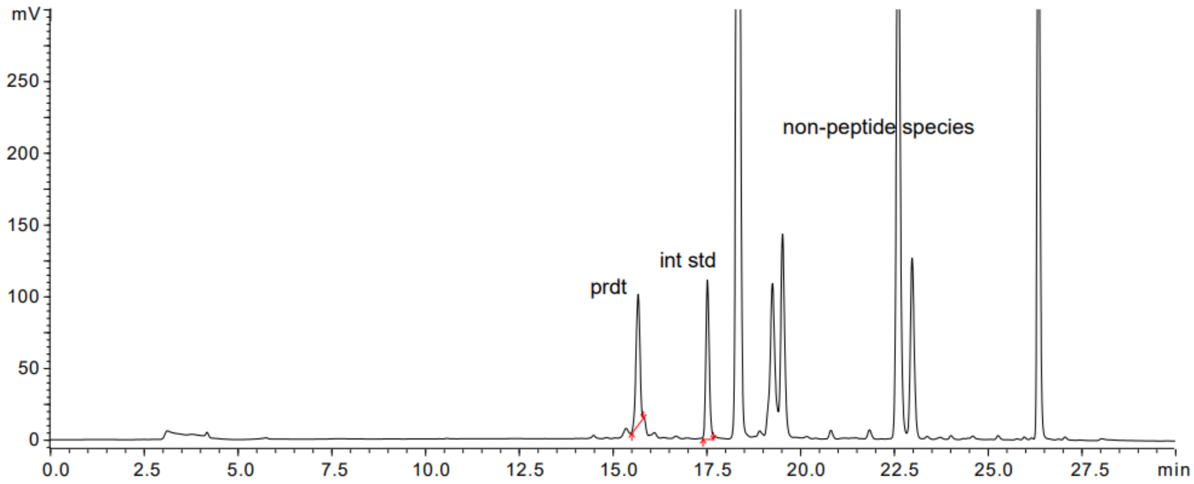

	peak area	yield
product (0.2 mM)	650991	94%
int std (0.2 mM)	692074	-

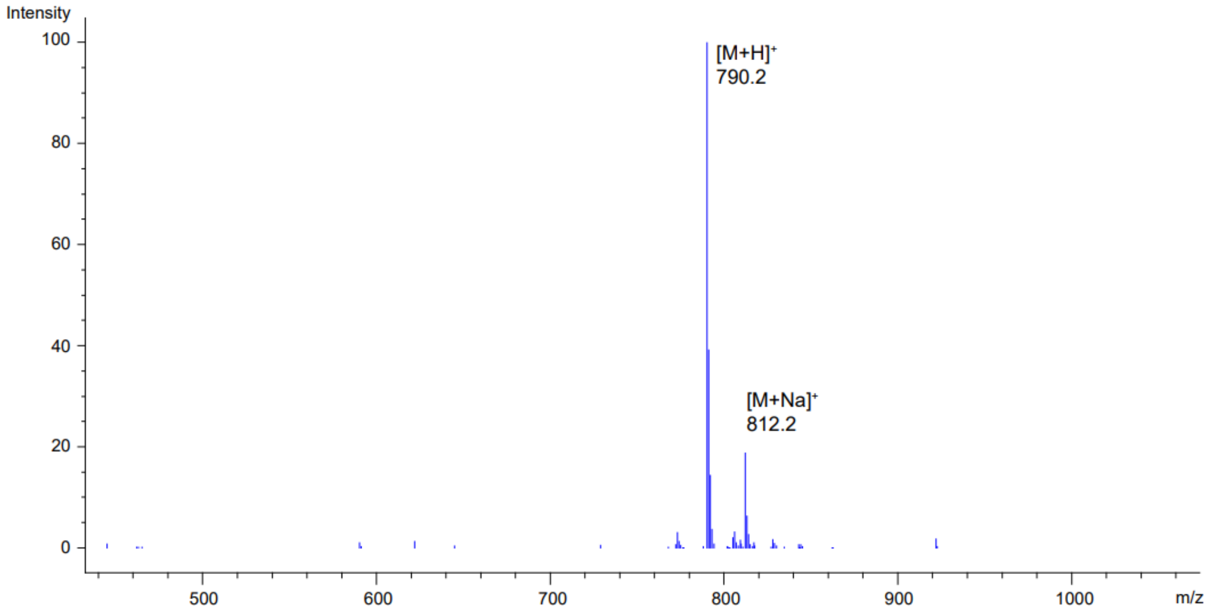

Figure S38. RP-HPLC trace of the reaction of N-H cross coupling of methionine sulfoximine peptide **1b** with 4-nitrophenylboronic acid at 350 nm (5-70% MeCN over 21 min) and ESI-MS spectrum of arylated product **1c**. m/z 790.2 and 812.2 correspond to $[M+H]^+$ and $[M+Na]^+$, respectively.

Synthesis of *N*-arylated peptide 2c: N-H coupling of methionine sulfoximine peptide 2b with 4-nitrophenylboronic acid

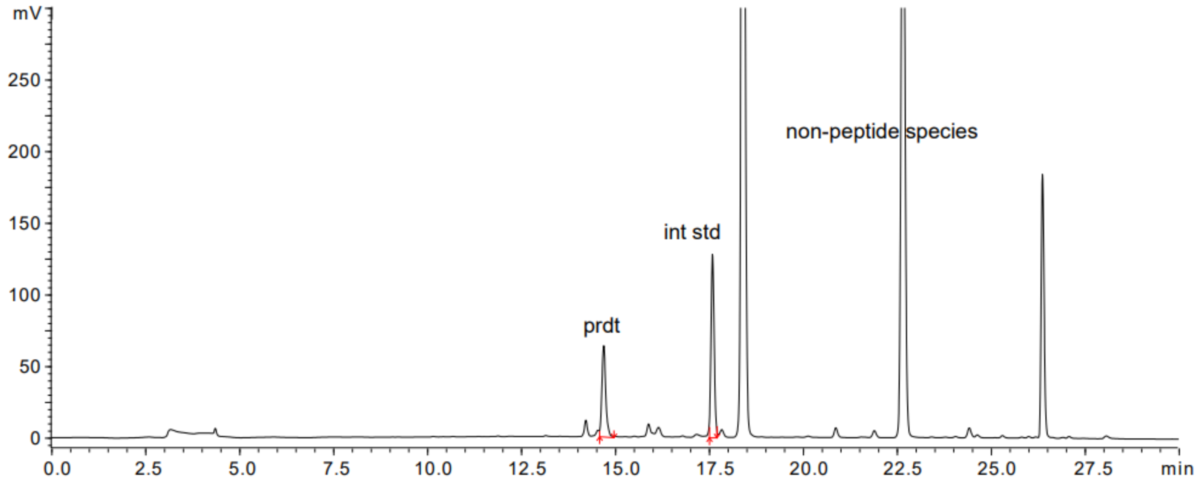

	peak area	yield
product (0.2 mM)	457157	61%
int std (0.2 mM)	747604	-

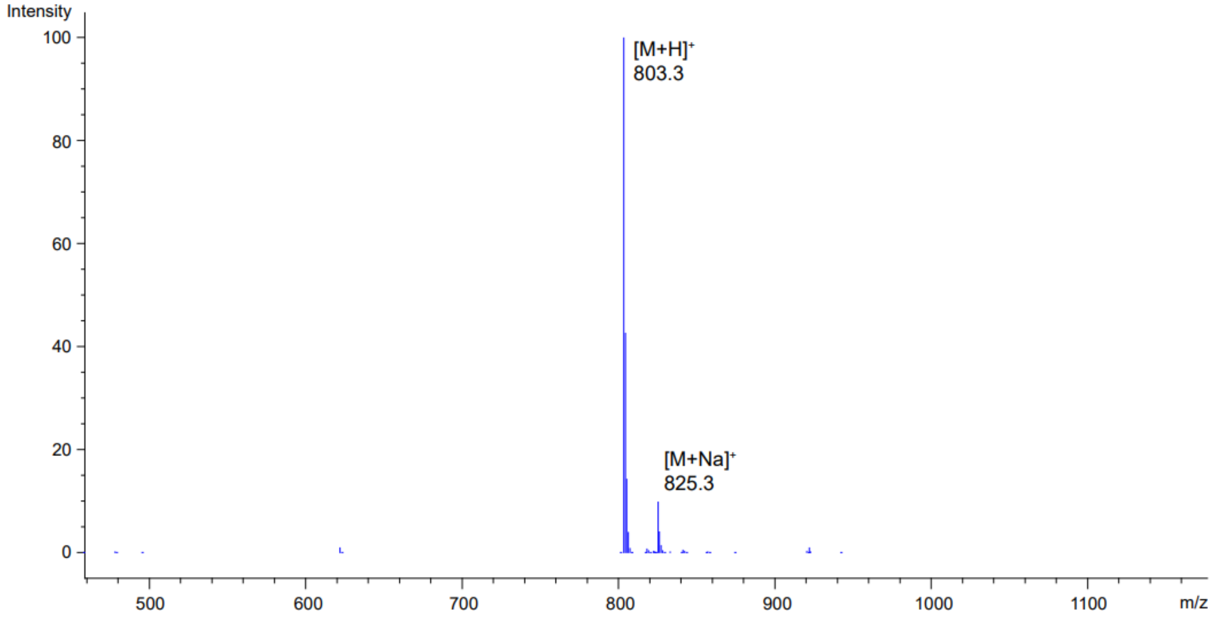

Figure S39. RP-HPLC trace of the reaction of N-H cross coupling of methionine sulfoximine peptide **2b** with 4-nitrophenylboronic acid at 350 nm (5-70% MeCN over 21 min) and ESI-MS spectrum of arylated product **2c**. m/z 803.3 and 825.3 correspond to $[M+H]^+$ and $[M+Na]^+$, respectively.

Synthesis of *N*-arylated peptide 3c: N-H coupling of methionine sulfoximine peptide 3b with 4-nitrophenylboronic acid

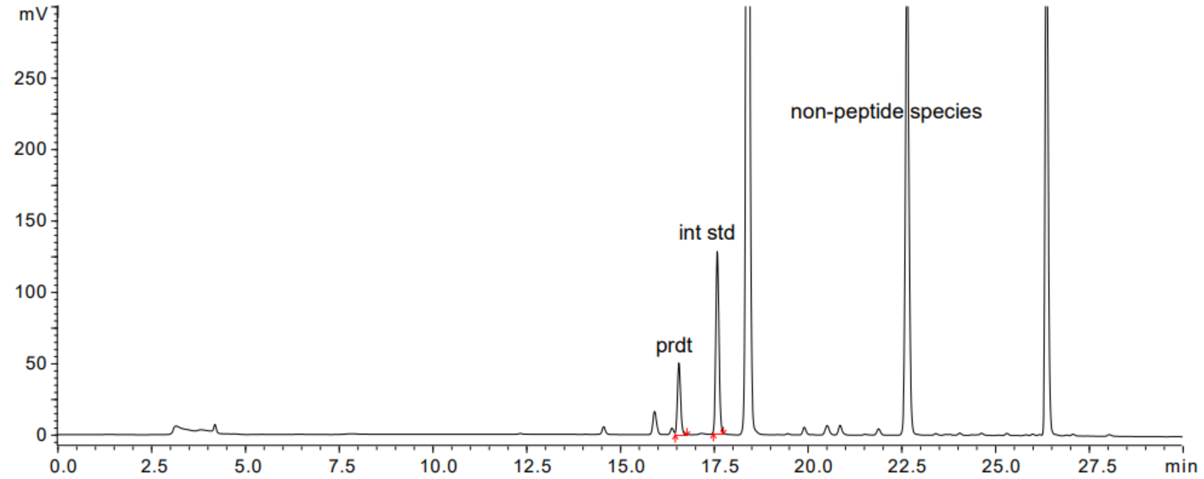

	peak area	yield
product (0.2 mM)	297002	40%
int std (0.2 mM)	751288	-

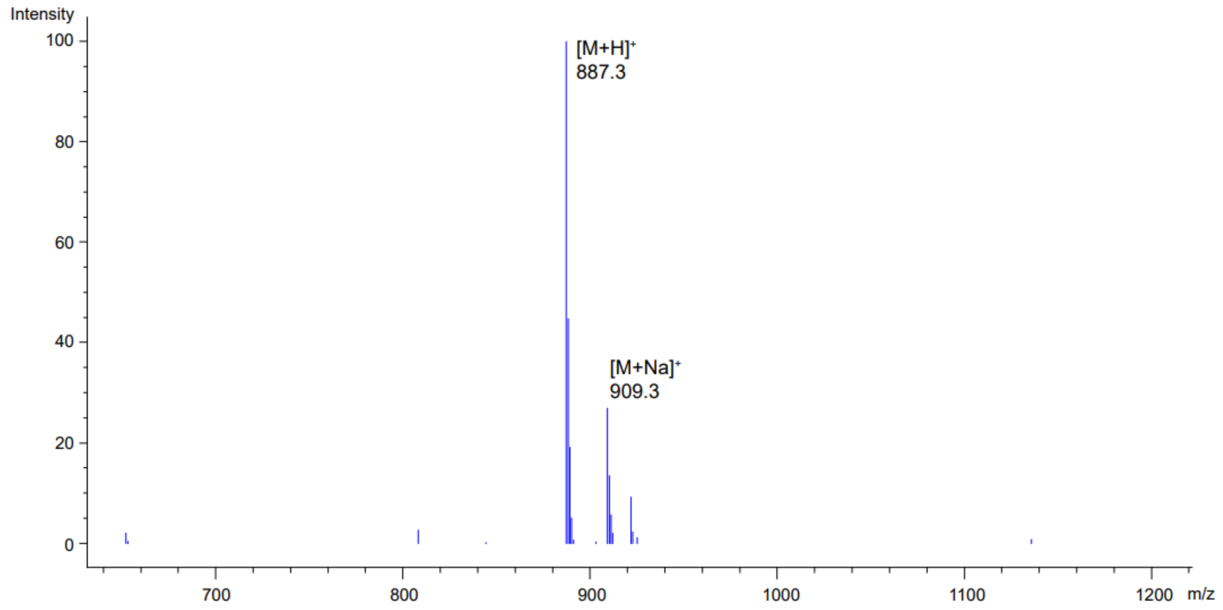

Figure S40. RP-HPLC trace of the reaction of N-H cross coupling of methionine sulfoximine peptide **3b** with 4-nitrophenylboronic acid at 350 nm (5-70% MeCN over 21 min) and ESI-MS spectrum of arylated product **3c**. m/z 887.3 and 909.3 correspond to $[M+H]^+$ and $[M+Na]^+$, respectively.

Synthesis of *N*-arylated peptide 4c: N-H coupling of methionine sulfoximine peptide 4b with 4-nitrophenylboronic acid

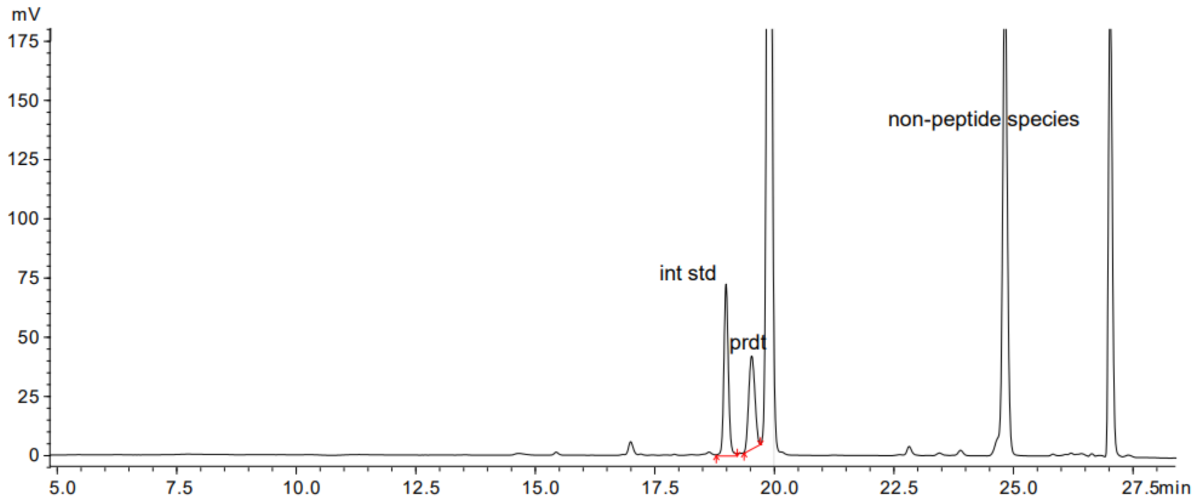

	peak area	yield
product (0.2 mM)	350491	75%
int std (0.2 mM)	464667	-

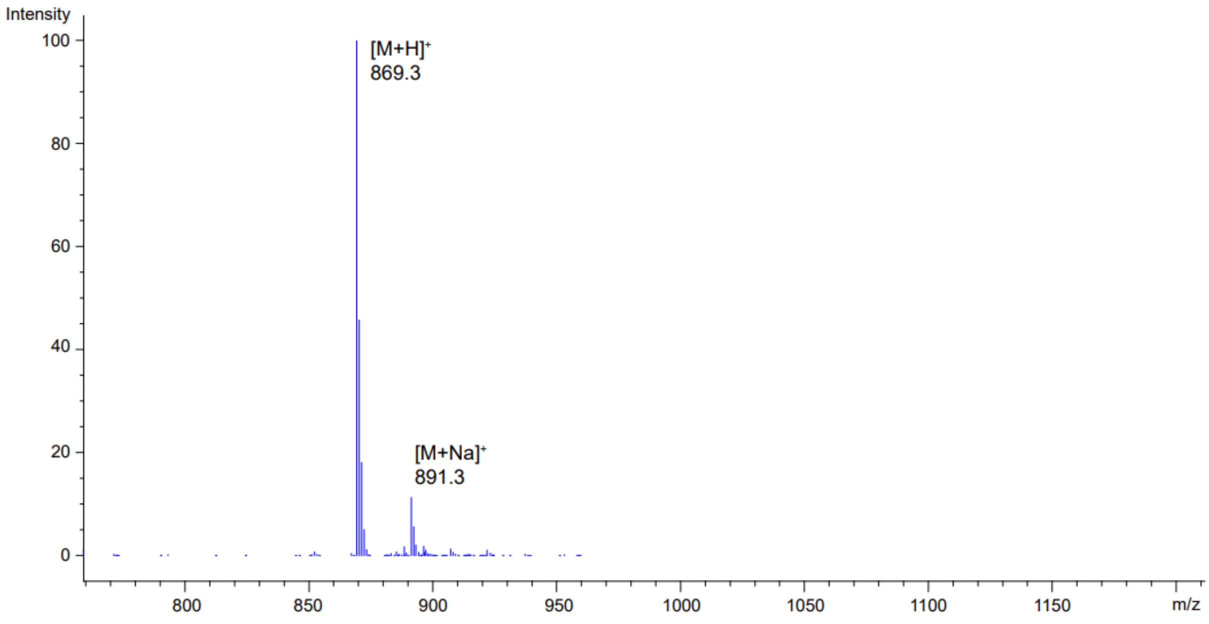

Figure S41. RP-HPLC trace of the reaction of N-H cross coupling of methionine sulfoximine peptide **4b** with 4-nitrophenylboronic acid at 350 nm (5-60% MeCN over 21 min) and ESI-MS spectrum of arylated product **4c**. m/z 869.3 and 891.3 correspond to $[M+H]^+$ and $[M+Na]^+$, respectively.

Synthesis of *N*-arylated peptide 5c: N-H coupling of methionine sulfoximine peptide 5b with 4-nitrophenylboronic acid

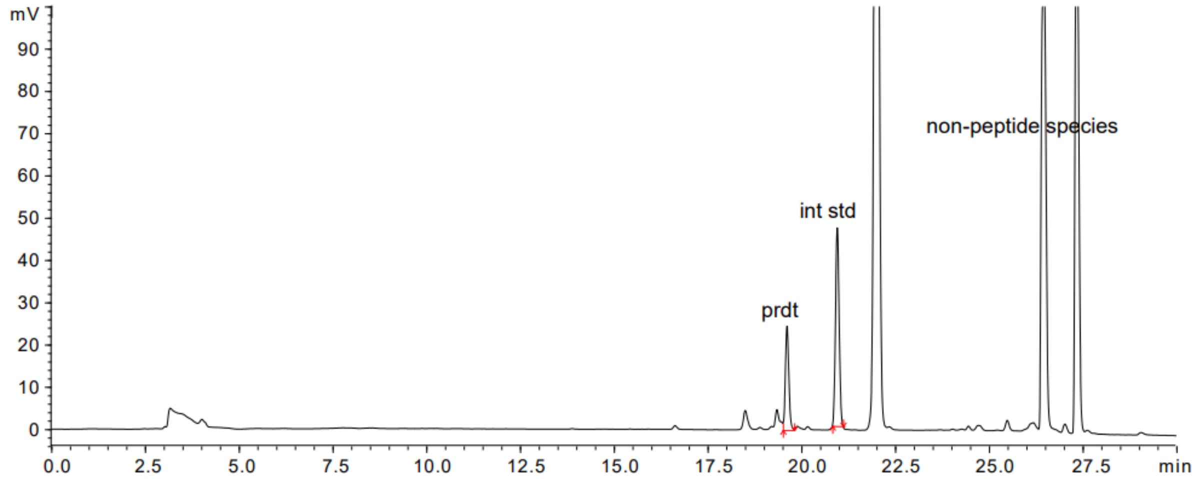

	peak area	yield
product (0.2 mM)	157199	50%
int std (0.2 mM)	315272	-

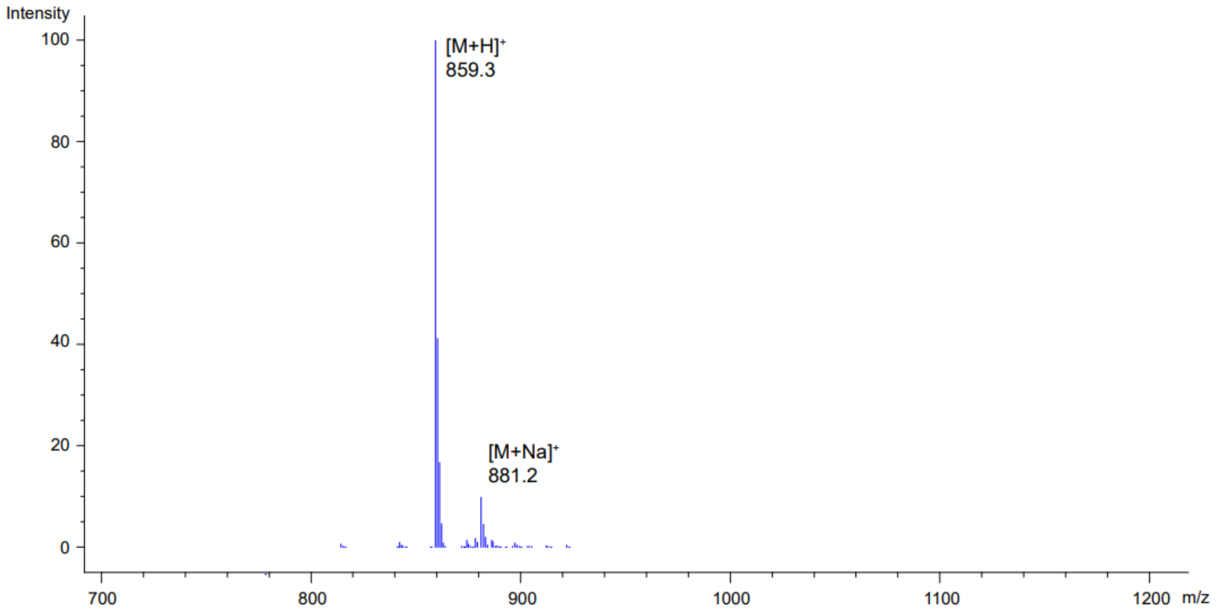

Figure S42. RP-HPLC trace of the reaction of N-H cross coupling of methionine sulfoximine peptide **5b** with 4-nitrophenylboronic acid at 350 nm (5-50% MeCN over 21 min) and ESI-MS spectrum of arylated product **5c**. m/z 859.3 and 881.3 correspond to $[M+H]^+$ and $[M+Na]^+$, respectively.

Synthesis of *N*-arylated peptide 6c: N-H coupling of methionine sulfoximine peptide 6b with 4-nitrophenylboronic acid

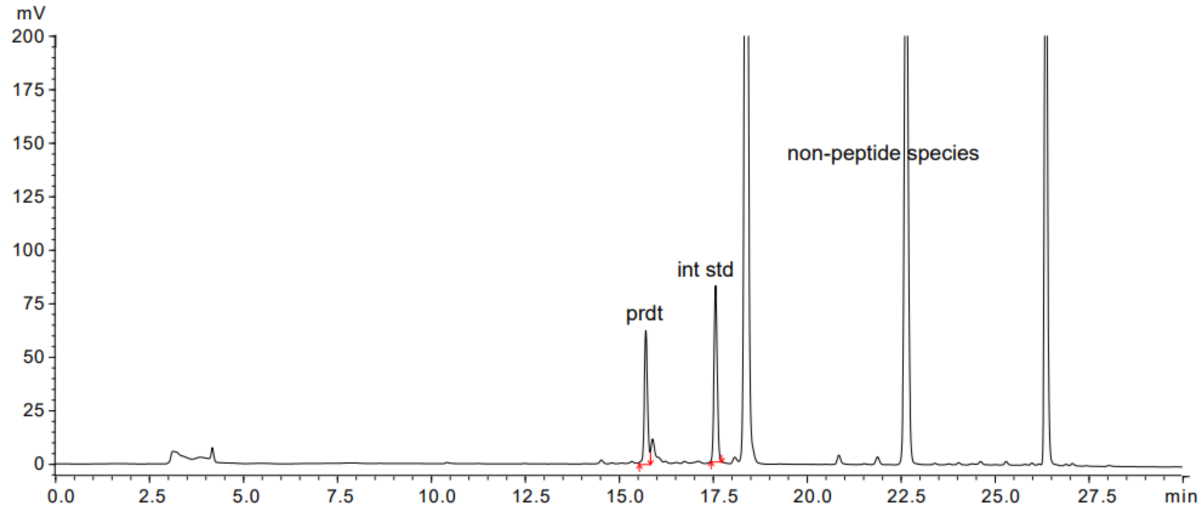

	peak area	yield
product (0.2 mM)	365449	76%
int std (0.2 mM)	481475	-

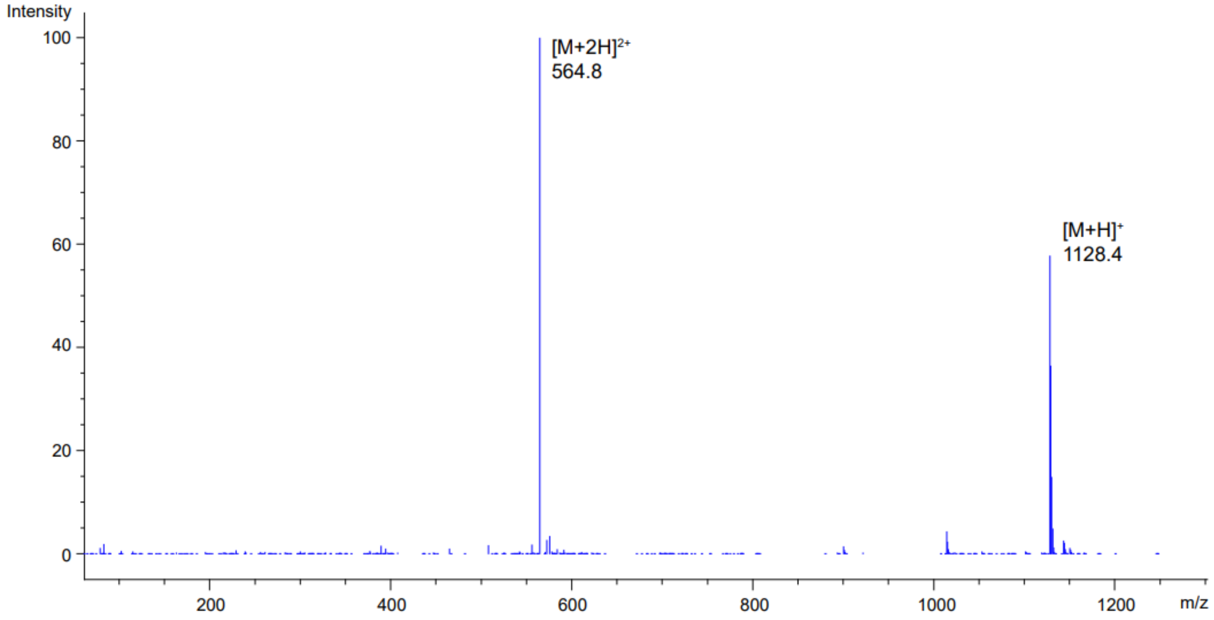

Figure S43. RP-HPLC trace of the reaction of N-H cross coupling of methionine sulfoximine peptide **6b** with 4-nitrophenylboronic acid at 350 nm (5-70% MeCN over 21 min) and ESI-MS spectrum of arylated product **6c**. m/z 564.8 and 1128.4 correspond to $[M+2H]^{2+}$ and $[M+H]^{+}$, respectively.

Synthesis of *N*-arylated peptide 7c: N-H coupling of methionine sulfoximine peptide 7b with 4-nitrophenylboronic acid

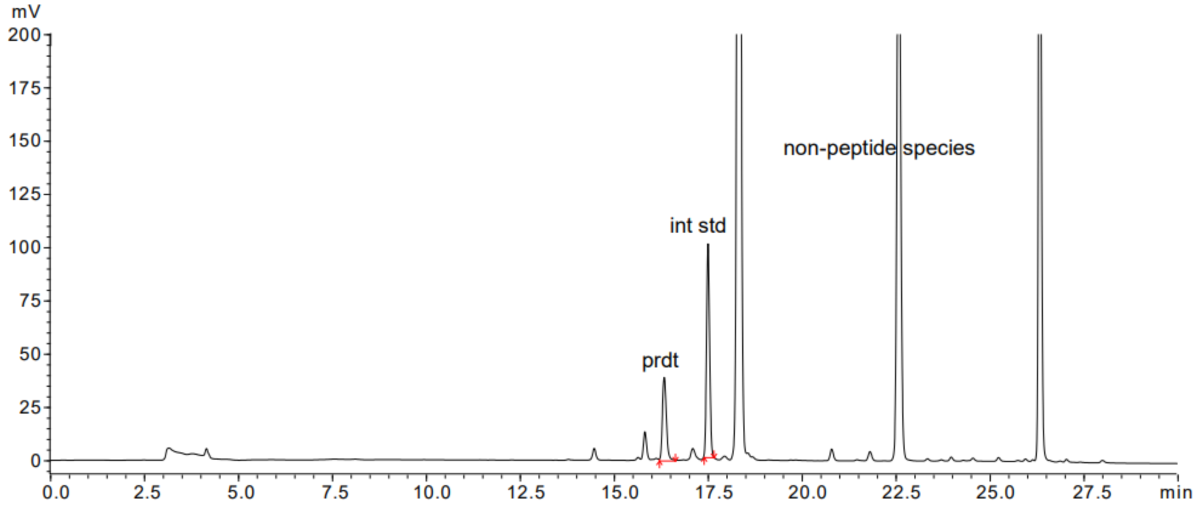

	peak area	yield
product (0.2 mM)	285617	50%
int std (0.2 mM)	575118	-

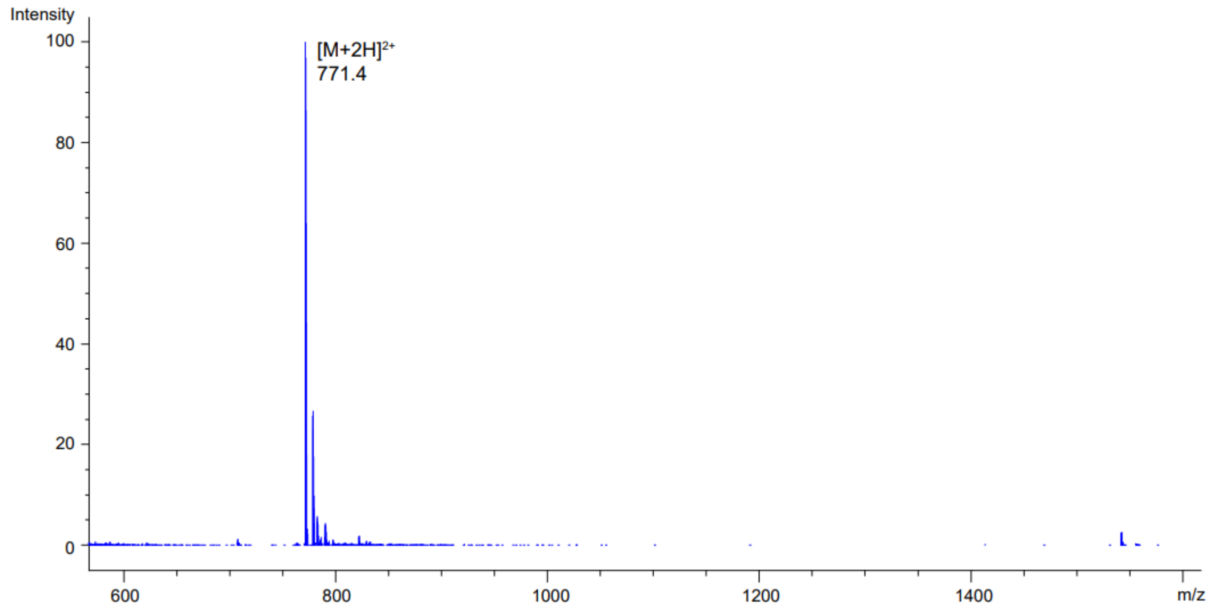

Figure S44. RP-HPLC trace of the reaction of N-H cross coupling of methionine sulfoximine peptide **7b** with 4-nitrophenylboronic acid at 350 nm (5-70% MeCN over 21 min) and ESI-MS spectrum of arylated product **7c**. m/z 771.4 correspond to $[M+2H]^{2+}$.

Synthesis of *N*-arylated peptide 8c: N-H coupling of methionine sulfoximine peptide 8b with 4-nitrophenylboronic acid

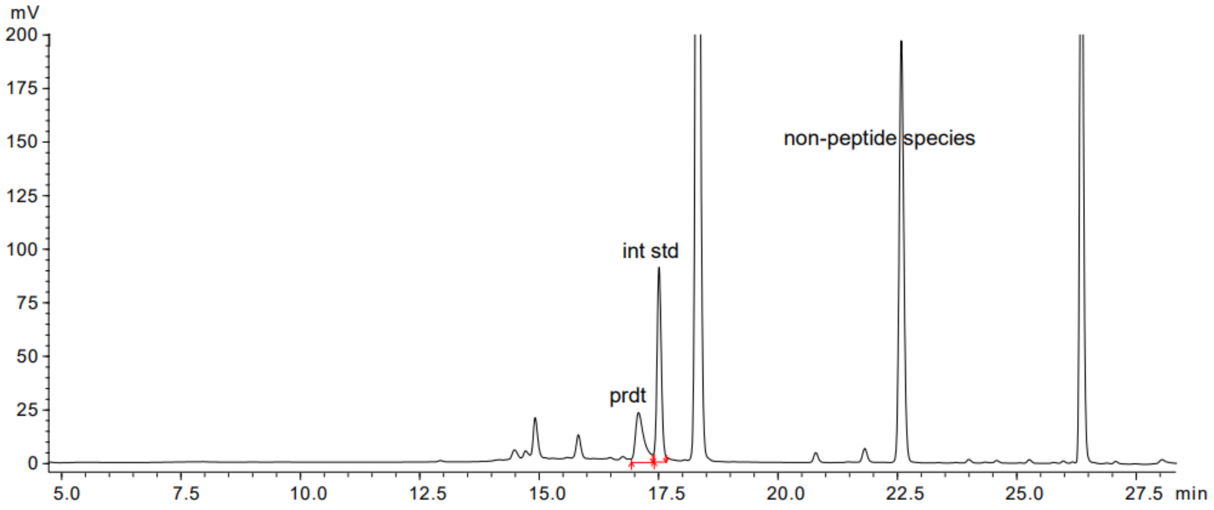

	peak area	yield
product (0.2 mM)	286801	51%
int std (0.2 mM)	566294	-

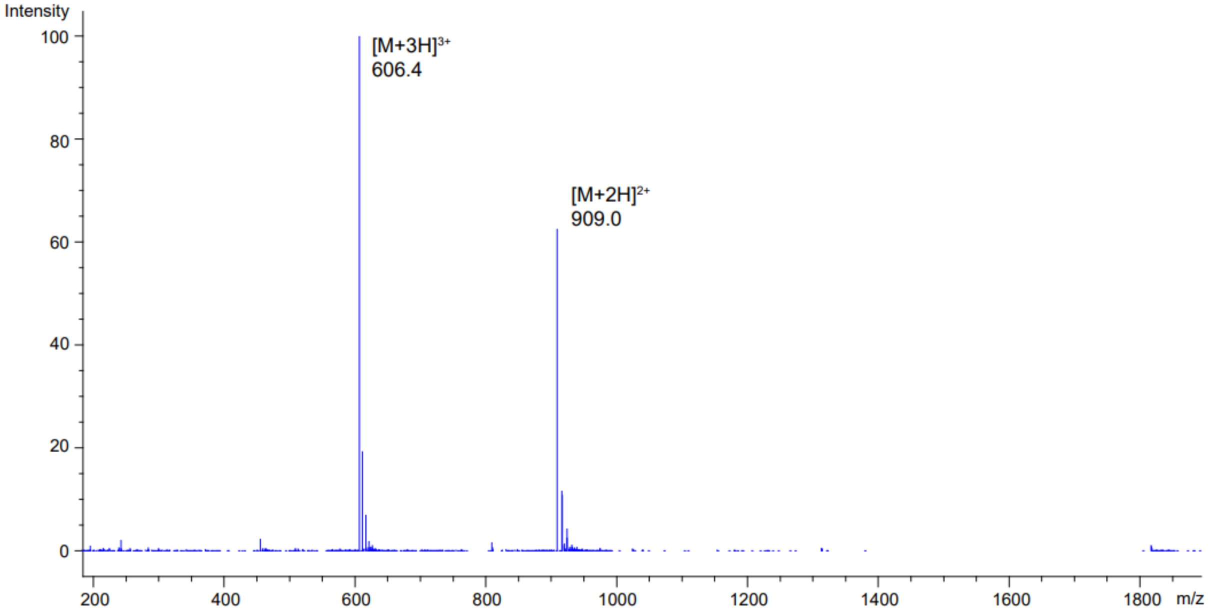

Figure S45. RP-HPLC trace of the reaction of N-H cross coupling of methionine sulfoximine peptide **8b** with 4-nitrophenylboronic acid at 350 nm (5-70% MeCN over 21 min) and ESI-MS spectrum of arylated product **8c**. m/z 606.4 and 909.0 correspond to $[M+3H]^{3+}$ and $[M+2H]^{2+}$, respectively.

Synthesis of peptide 1e: N–H cross-coupling of methionine sulfoximine peptide 1b with potassium 3-(azidomethyl)phenyltrifluoroborate (Figure 3, a)

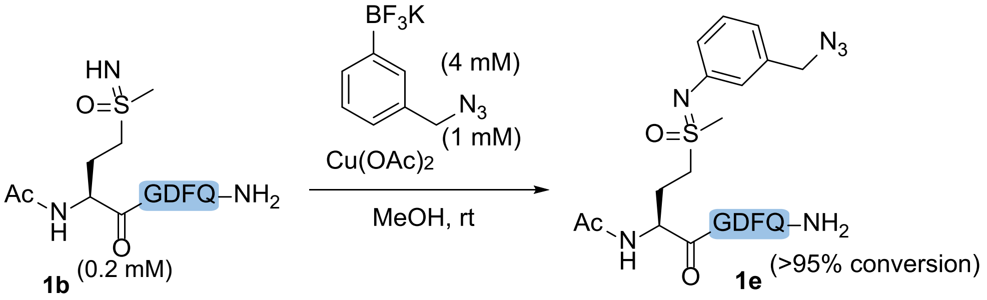

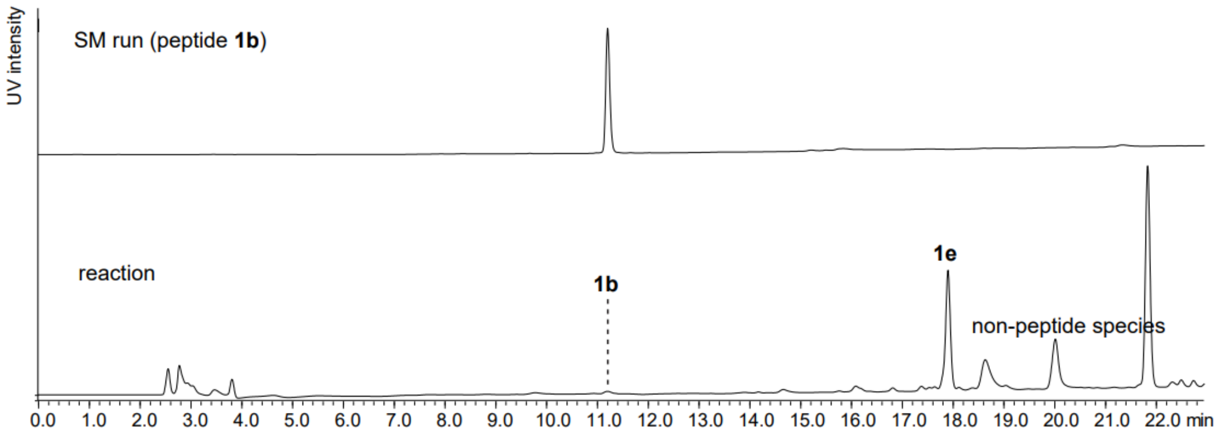

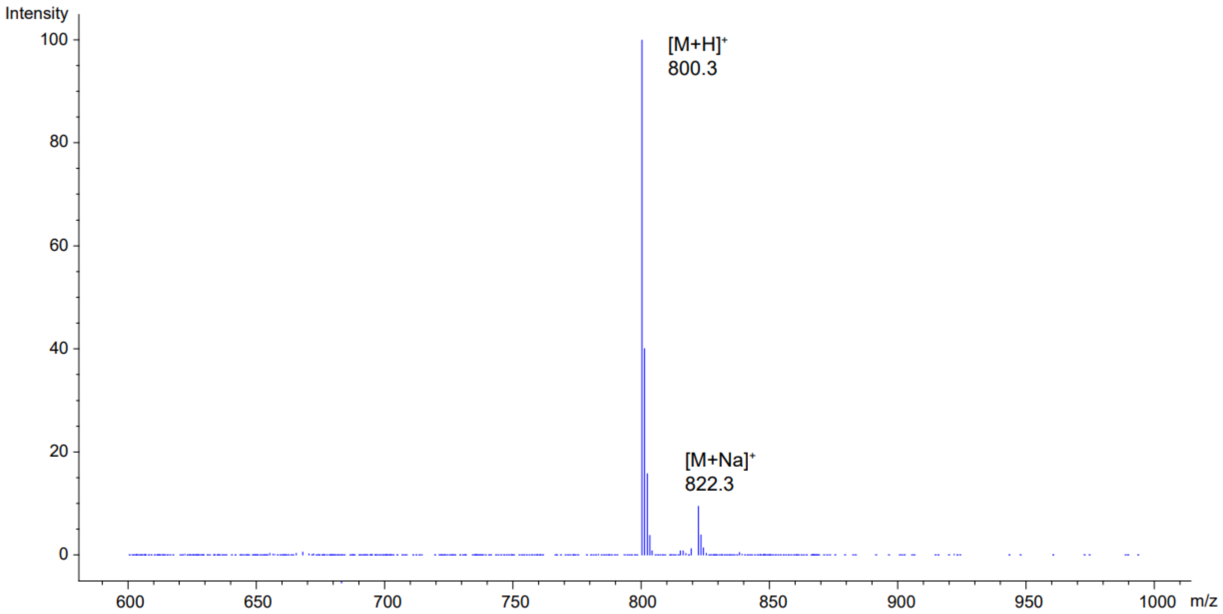

Figure S46. RP-HPLC traces of peptide **1b** (above) and the reaction of N–H cross-coupling of **1b** with potassium 3-(azidomethyl)phenyltrifluoroborate (below) at 254 nm (5-50% MeCN over 18 min) and ESI-MS spectrum of the coupling product. m/z 800.3 and 822.3 correspond to $[M+H]^+$ and $[M+Na]^+$, respectively.

Synthesis of peptide 5: NH-alkylation of methionine sulfoximine peptide 4 (Figure 3, b)

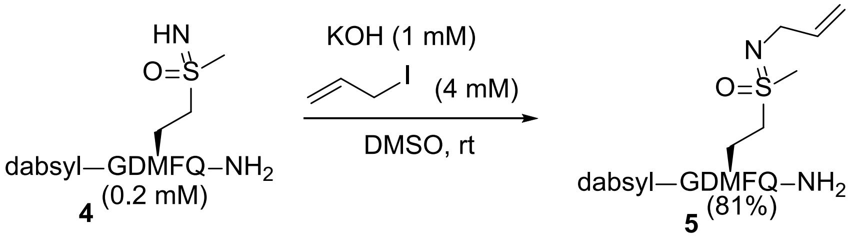

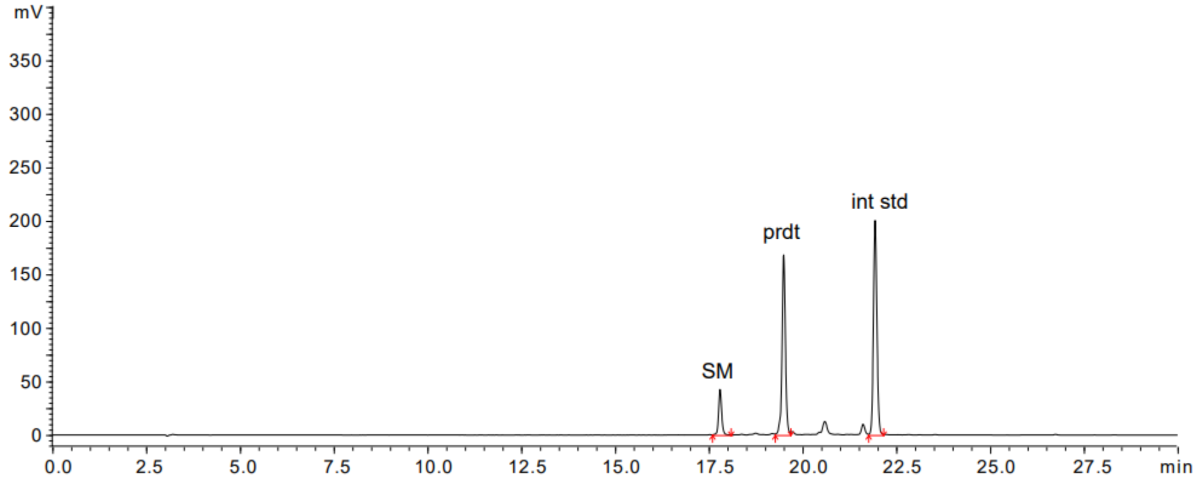

	peak area	yield
product (0.2 mM)	1065846	81%
int std (0.2 mM)	1313542	-

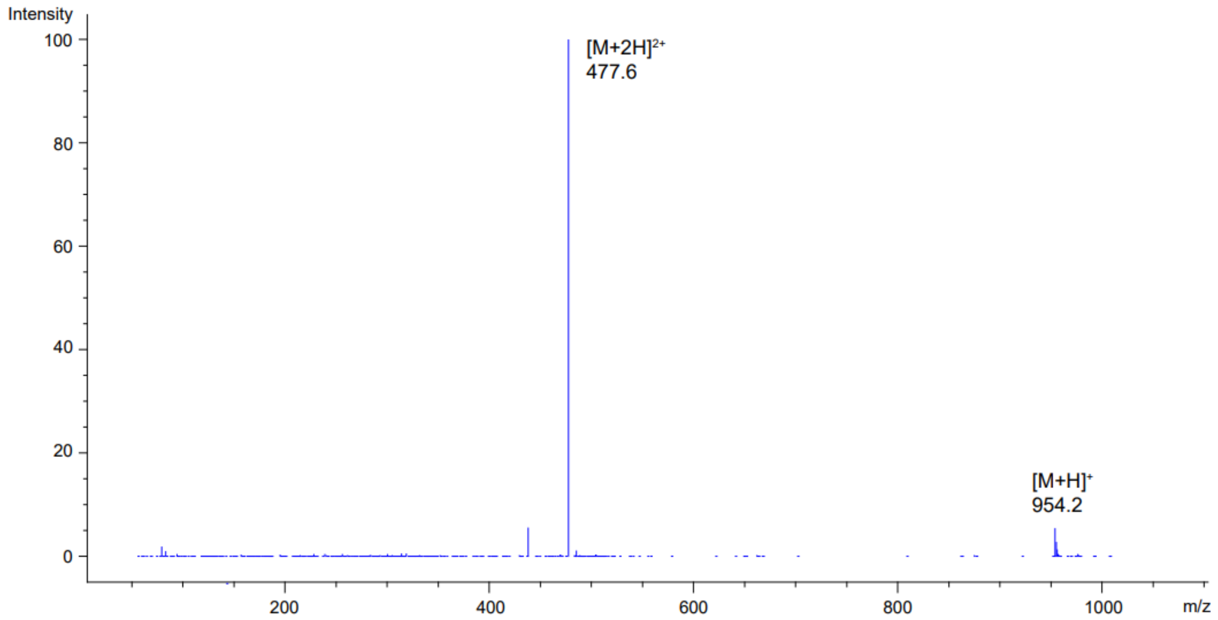

Figure S47. RP-HPLC trace of the reaction of N-H alkylation of dabsyl-labeled methionine sulfoximine peptide **4** with allyl iodide at 500 nm (5-65% MeCN over 21 min) and ESI-MS spectrum of the alkylated product. m/z 477.6 and 954.2 correspond to $[M+2H]^{2+}$ and $[M+H]^{+}$, respectively.

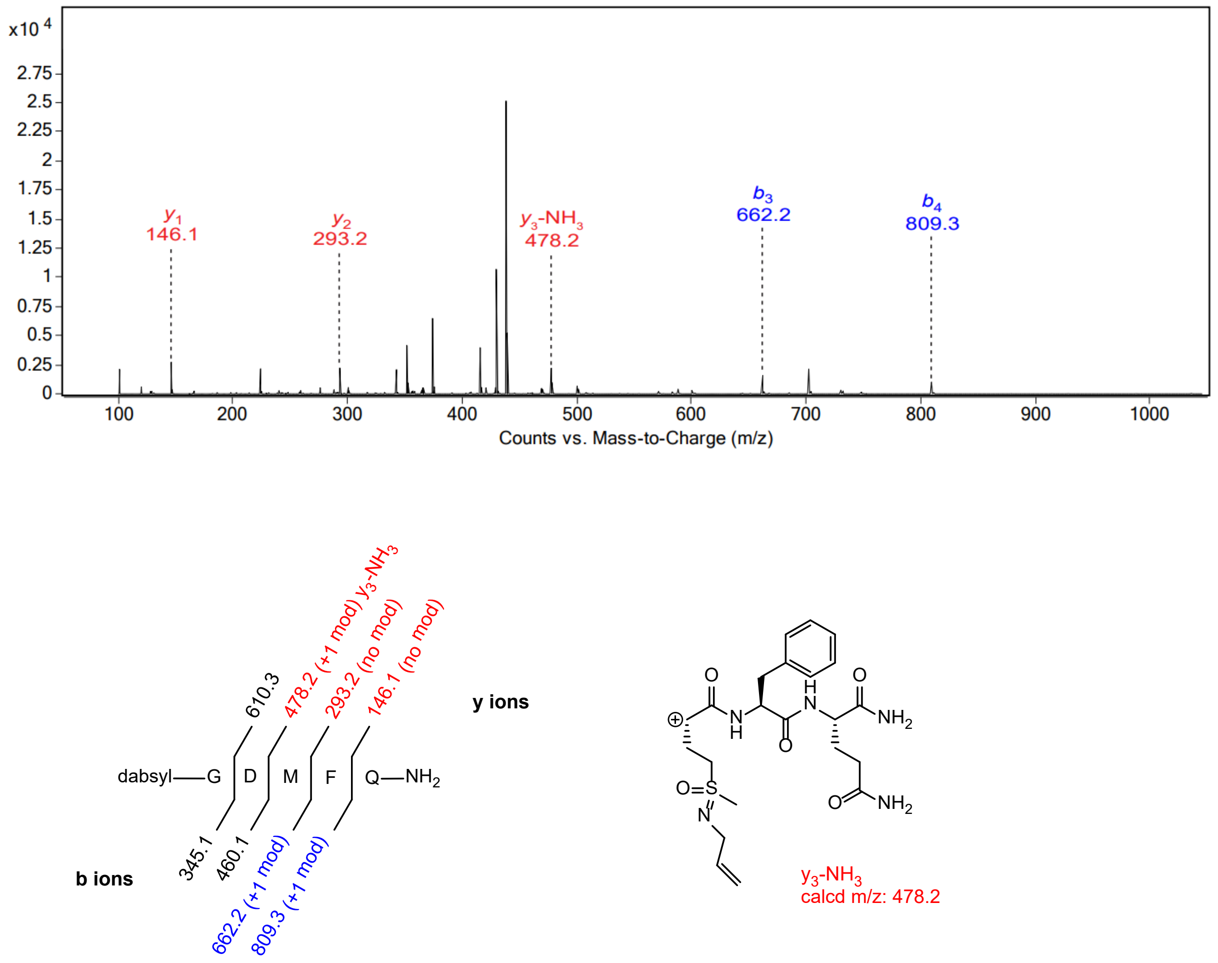

Figure S48. LC-MS/MS spectrum and fragmentation ladder of NH-alkylation product **5**.

Synthesis of peptide 1f: Carbonylation and ^{13}C labeling of methionine sulfoximine peptide 1b (Figure 3, c)

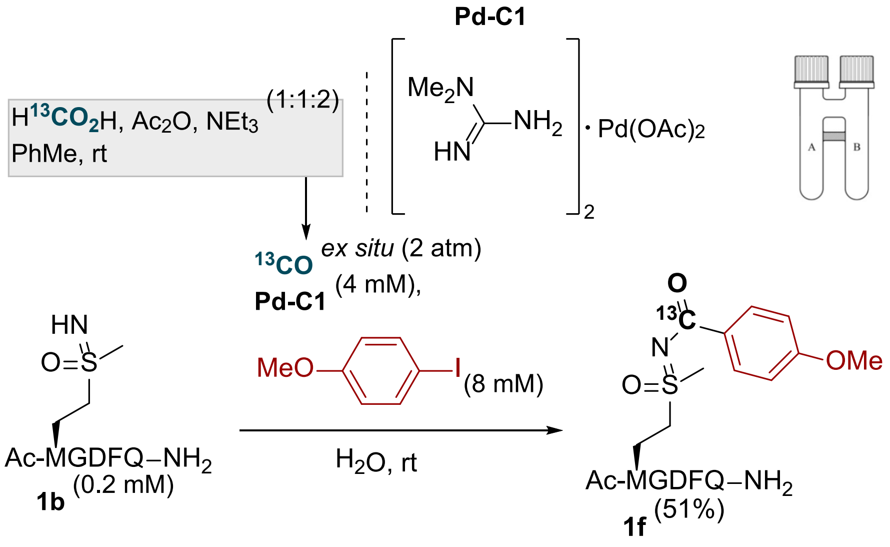

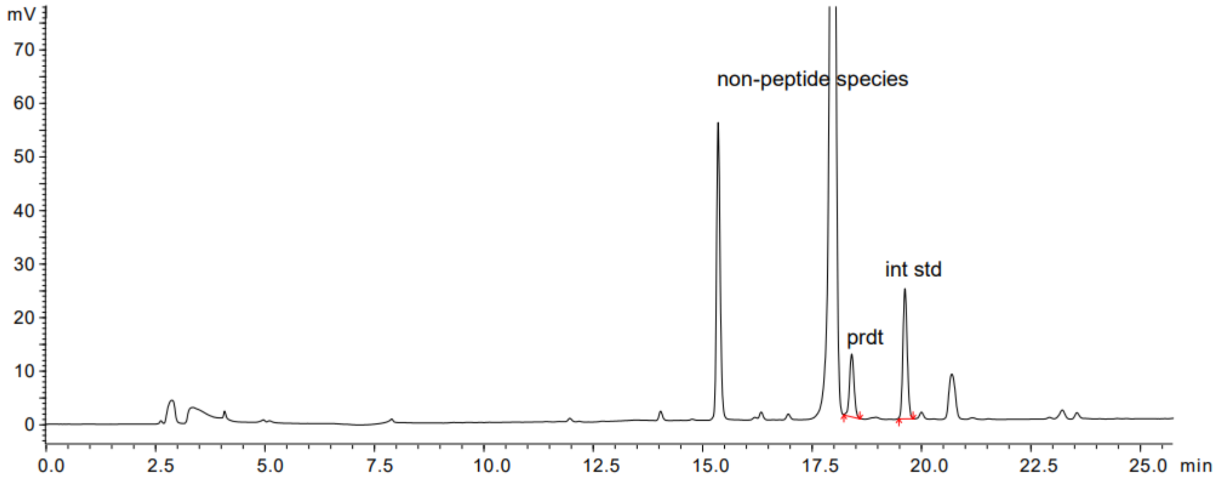

	peak area	yield
product (0.2 mM)	83030	51%
int std (0.2 mM)	164203	-

Figure S49. RP-HPLC trace of the reaction of carbonylation and ^{13}C labeling of methionine sulfoximine peptide **1b** at 300 nm (5-50% MeCN over 21 min)

a)

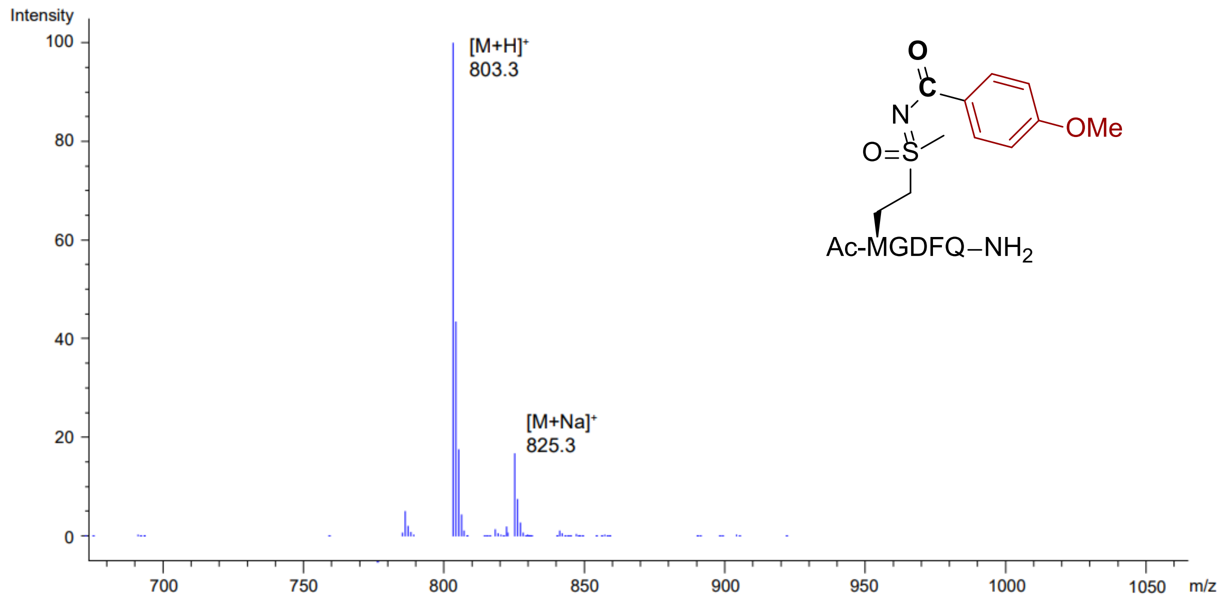

b)

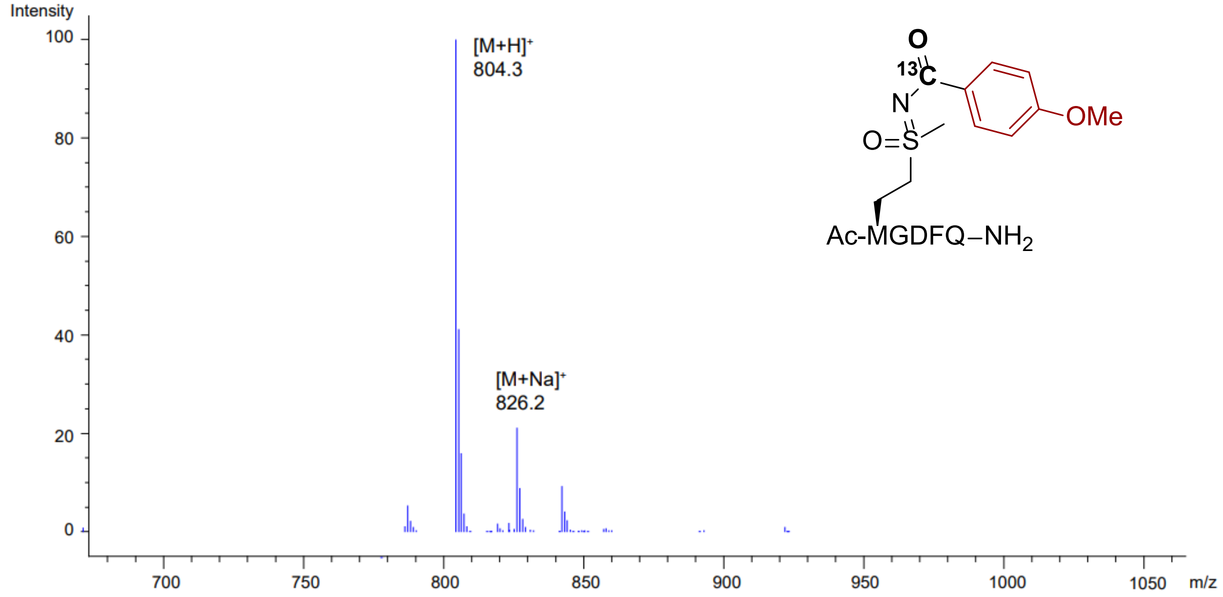

Figure S50. a) ESI-MS spectrum of the ^{12}C-carbonylated product. m/z 803.3 and 825.3 correspond to $[M+H]^+$ and $[M+Na]^+$, respectively. b) ESI-MS spectrum of the ^{13}C-carbonylated product. m/z 804.3 and 826.2 correspond to $[M+H]^+$ and $[M+Na]^+$, respectively.

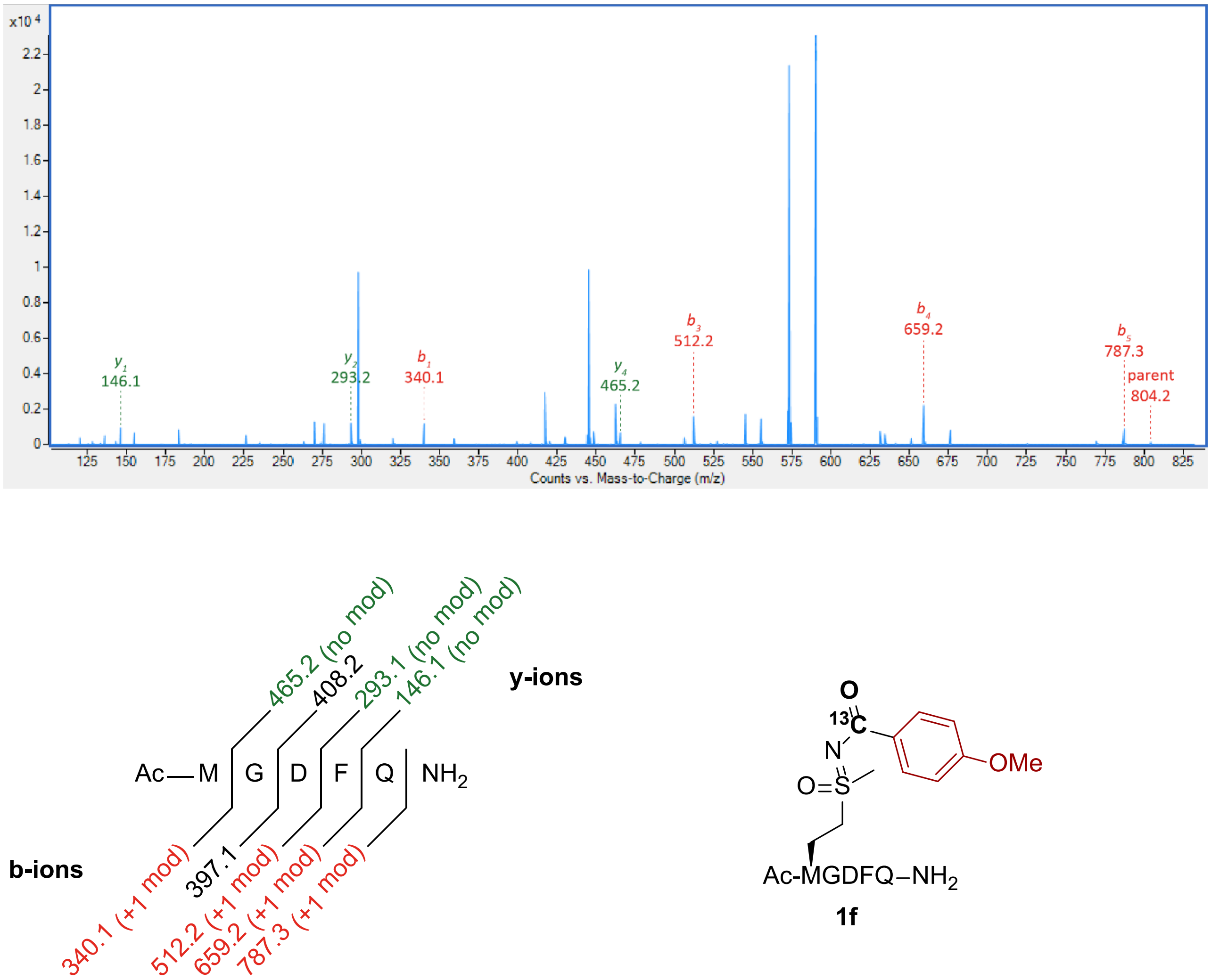

1f

Figure S51. LC-MS/MS spectrum and fragmentation ladder of ^{13}C-labeled carbonylated product **1f**.

For Figure 4

Glutamine synthetase inhibition assay

Table S4. Assay 1

Sample	A_{570} 1	A_{570} 2	A_{570} 3	A_{570} average	Background subtraction	Inhibition (%)
Background control	0.074	0.080	0.083	0.079	-	-
Enzyme control	0.649	0.655	0.656	0.653	0.574	-
MSO	0.547	0.549	0.552	0.549	0.470	18.1
**1b**	0.552	0.561	0.557	0.557	0.478	16.8
**2b**	0.482	0.487	0.488	0.486	0.407	29.2
**3b**	0.642	0.651	0.652	0.648	0.569	0.9
**4b**	0.572	0.579	0.580	0.577	0.498	13.3
**5b**	0.594	0.602	0.600	0.599	0.520	9.5
**6b**	0.485	0.487	0.496	0.489	0.410	28.6
**7b**	0.638	0.648	0.645	0.644	0.565	1.7
**8b**	0.445	0.450	0.457	0.451	0.372	35.3

Table S5. Assay 2

Sample	A_{570} 1	A_{570} 2	A_{570} 3	A_{570} average	Background subtraction	Inhibition (%)
Background control	0.072	0.073	0.076	0.074	-	-
Enzyme control	0.650	0.651	0.656	0.652	0.579	-
MSO	0.540	0.546	0.545	0.544	0.470	18.8
**1b**	0.550	0.556	0.553	0.553	0.479	17.2
**2b**	0.481	0.489	0.491	0.487	0.413	28.6
**3b**	0.640	0.649	0.650	0.646	0.573	1.0
**4b**	0.559	0.562	0.567	0.563	0.489	15.5
**5b**	0.572	0.577	0.583	0.577	0.504	13.0
**6b**	0.493	0.504	0.506	0.501	0.427	26.2
**7b**	0.630	0.634	0.639	0.634	0.561	3.1
**8b**	0.451	0.457	0.465	0.458	0.384	33.6

Table S6. Assay 3

Sample	A_{570} 1	A_{570} 2	A_{570} 3	A_{570} average	Background subtraction	Inhibition (%)
Background control	0.076	0.082	0.086	0.081	-	-
Enzyme control	0.645	0.644	0.649	0.646	0.565	-
MSO	0.530	0.535	0.547	0.537	0.456	19.2
**1b**	0.534	0.539	0.543	0.539	0.457	19.0
**2b**	0.458	0.458	0.463	0.460	0.378	33.0
**3b**	0.639	0.641	0.645	0.642	0.560	0.8
**4b**	0.561	0.565	0.570	0.565	0.484	14.3
**5b**	0.571	0.579	0.583	0.578	0.496	12.1
**6b**	0.507	0.510	0.513	0.510	0.429	24.1
**7b**	0.622	0.625	0.629	0.625	0.544	3.7
**8b**	0.438	0.440	0.449	0.442	0.361	36.1

Table S7. Average of assay 1, 2 and 3

Sample	Average inhibition (%)	Standard deviation
MSO	18.7	0.984
**1b**	17.7	1.19
**2b**	30.3	2.16
**3b**	0.9	0.737
**4b**	14.4	1.16
**5b**	11.5	1.75
**6b**	26.3	2.11
**7b**	2.8	1.11
**8b**	35.0	1.44

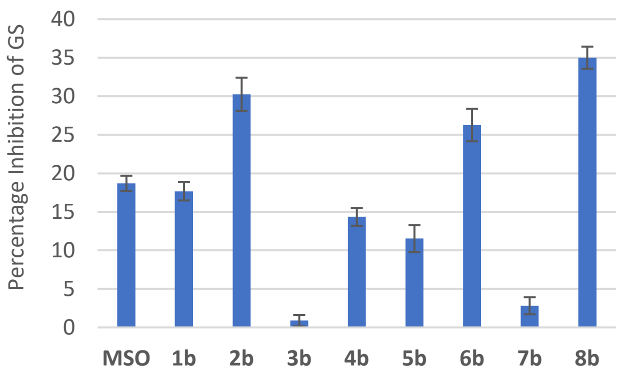

Chart 1. Inhibition of glutamine synthetase (GS) by methionine sulfoximine (MSO) and methionine sulfoximine-containing peptides (**1b-8b**). Assays were performed at 1 mM inhibitor concentration using a glutamine synthetase activity assay kit (Abcam).

Characterization Data

Synthesized peptide starting materials

Ac-MGDFQ-NH_2 (1a)

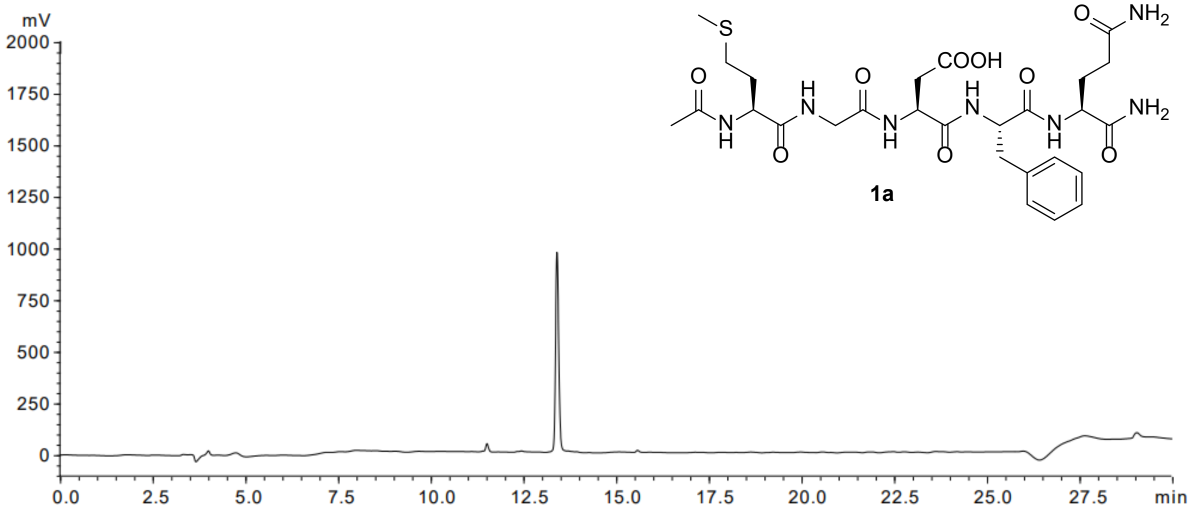

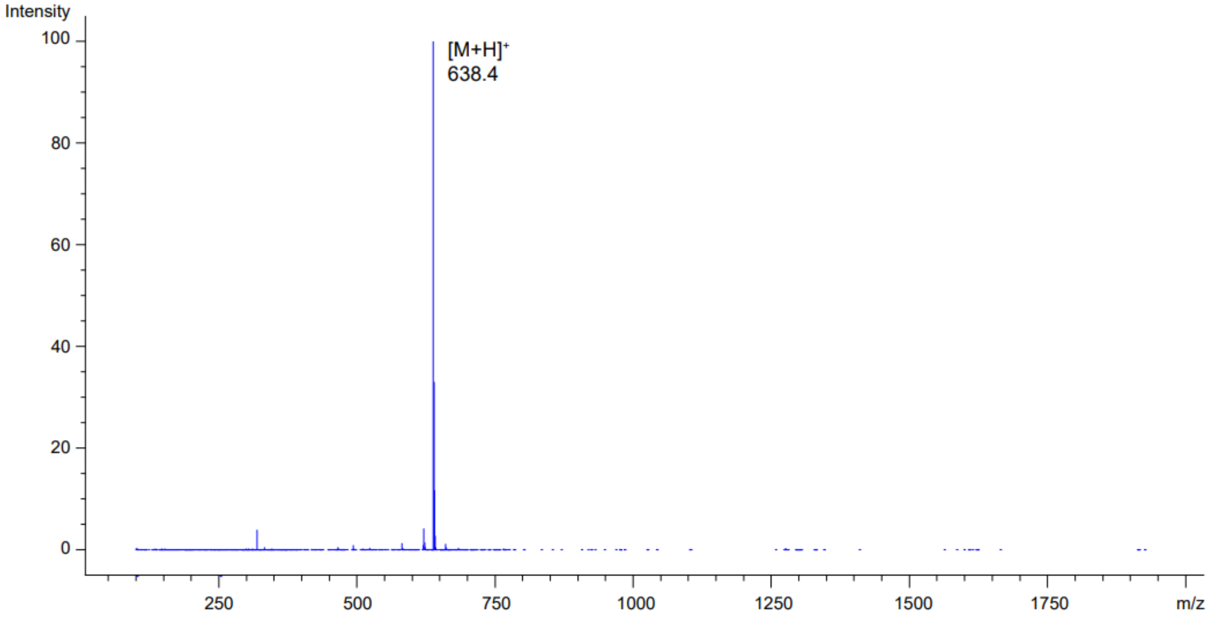

Figure S52. RP-HPLC trace at 220 nm (5-70% MeCN over 21 min) and ESI-MS spectrum of purified peptide starting material **1a**. m/z 638.4 correspond to $[M+H]^+$.

Ac-MGKFQ-NH₂ (2a)

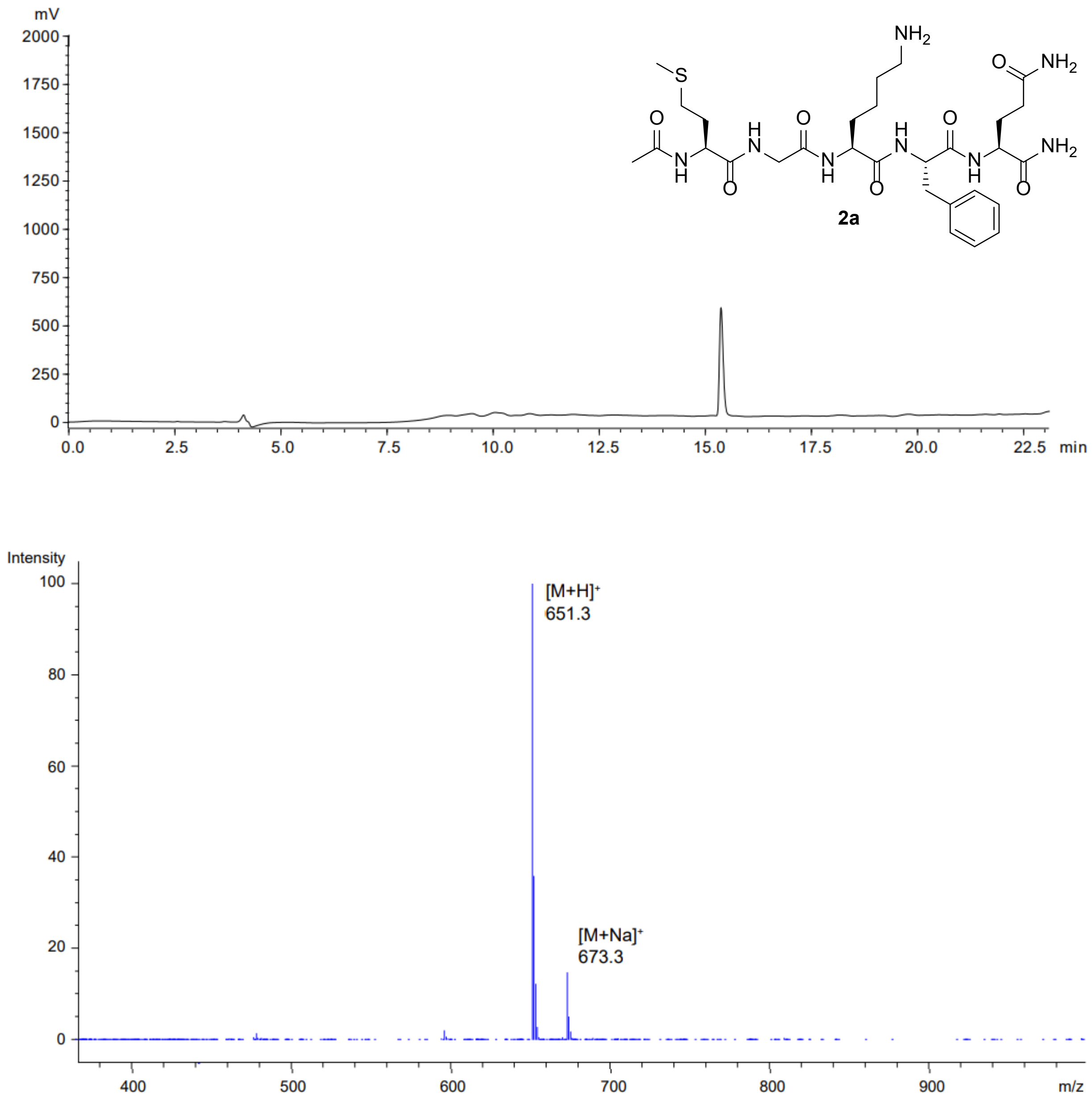

Figure S53. RP-HPLC trace at 220 nm (5-50% MeCN over 18 min) and ESI-MS spectrum of purified peptide starting material **2a**. m/z 651.3 and 673.3 correspond to $[M+H]^+$ and $[M+Na]^+$, respectively.

Ac-YGMLNP-NH₂ (3a)

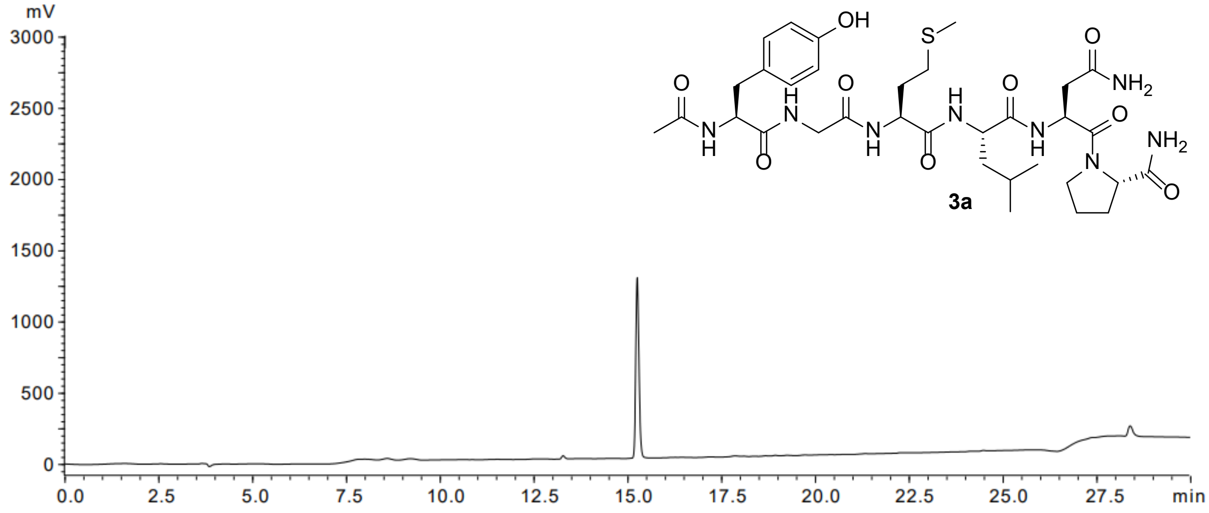

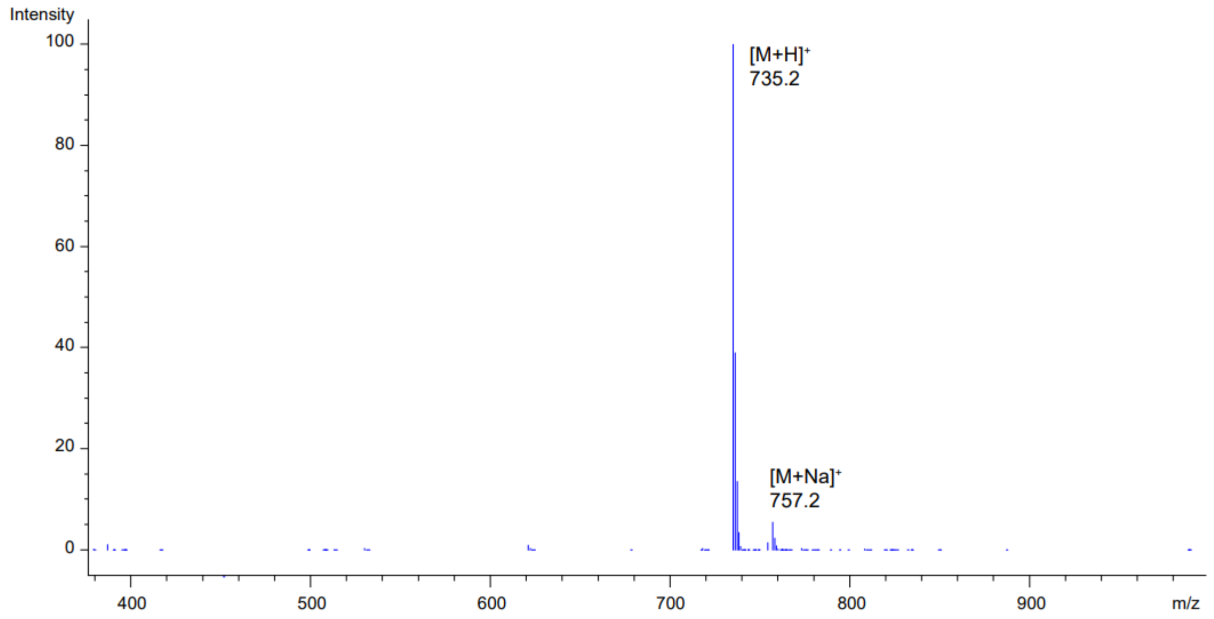

Figure S54. RP-HPLC trace at 220 nm (5-70% MeCN over 21 min) and ESI-MS spectrum of purified peptide starting material **3a**. m/z 735.2 and 757.2 correspond to $[M+H]^+$ and $[M+Na]^+$, respectively.

Ac-VGMSWP-NH$_2$ (4a)

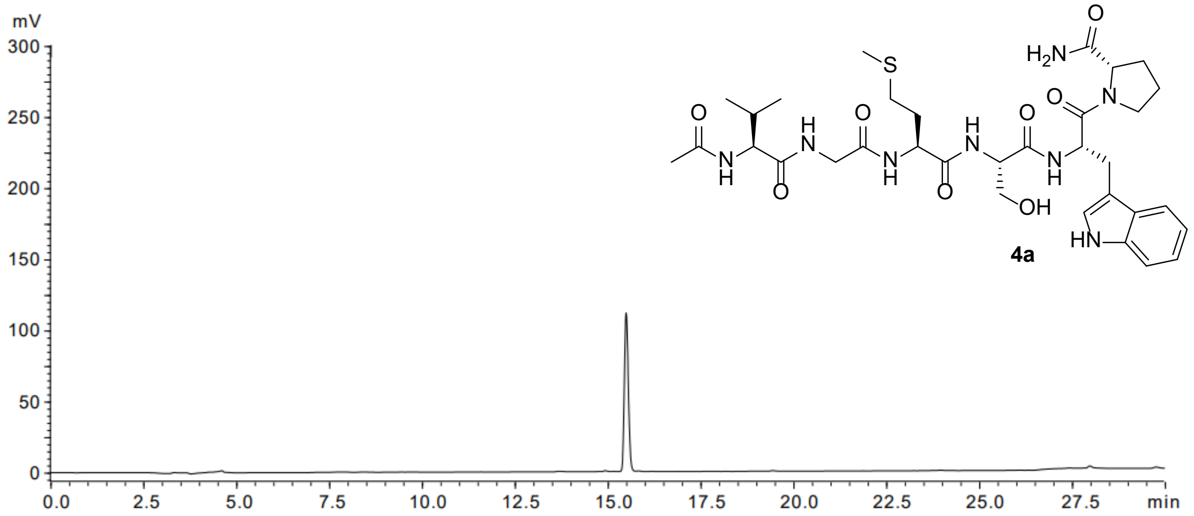

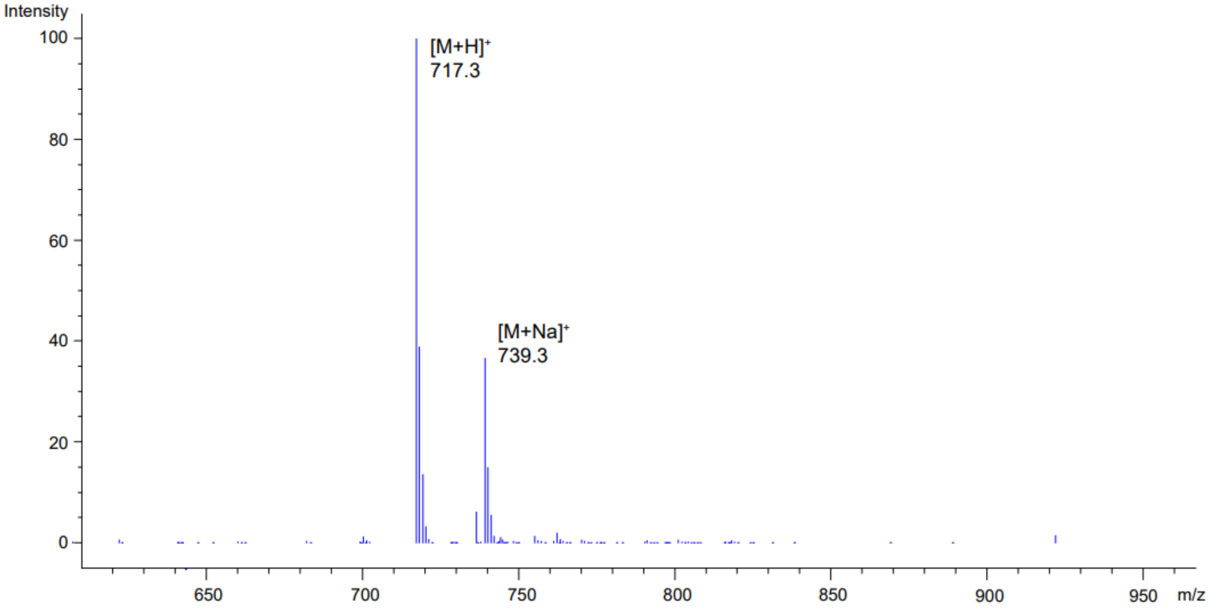

Figure S55. RP-HPLC trace at 220 nm (5-70% MeCN over 21 min) and ESI-MS spectrum of purified peptide starting material **4a**. m/z 717.3 and 739.3 correspond to $[M+H]^+$ and $[M+Na]^+$, respectively.

Ac-FPQSGM-NH_2 (5a)

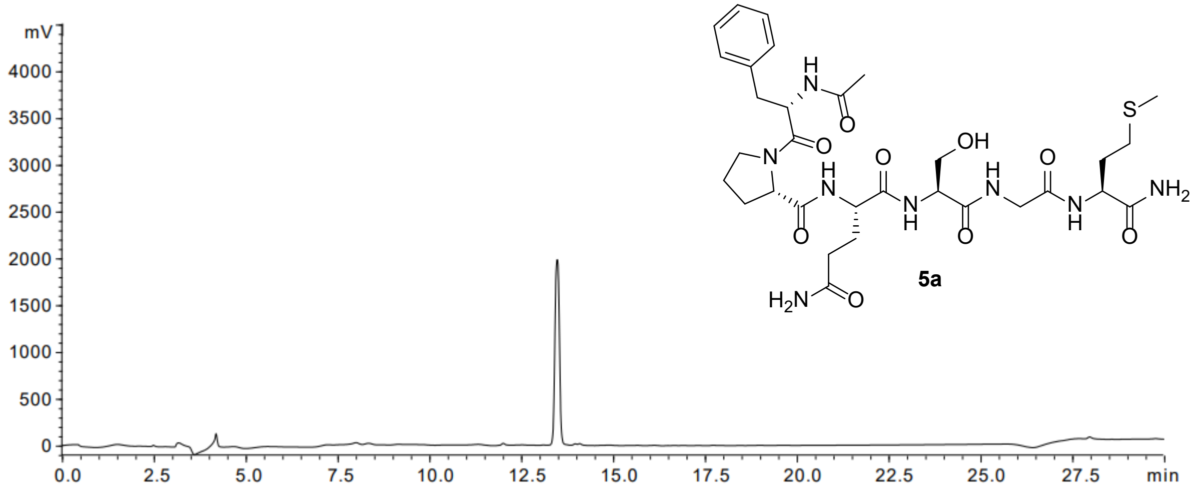

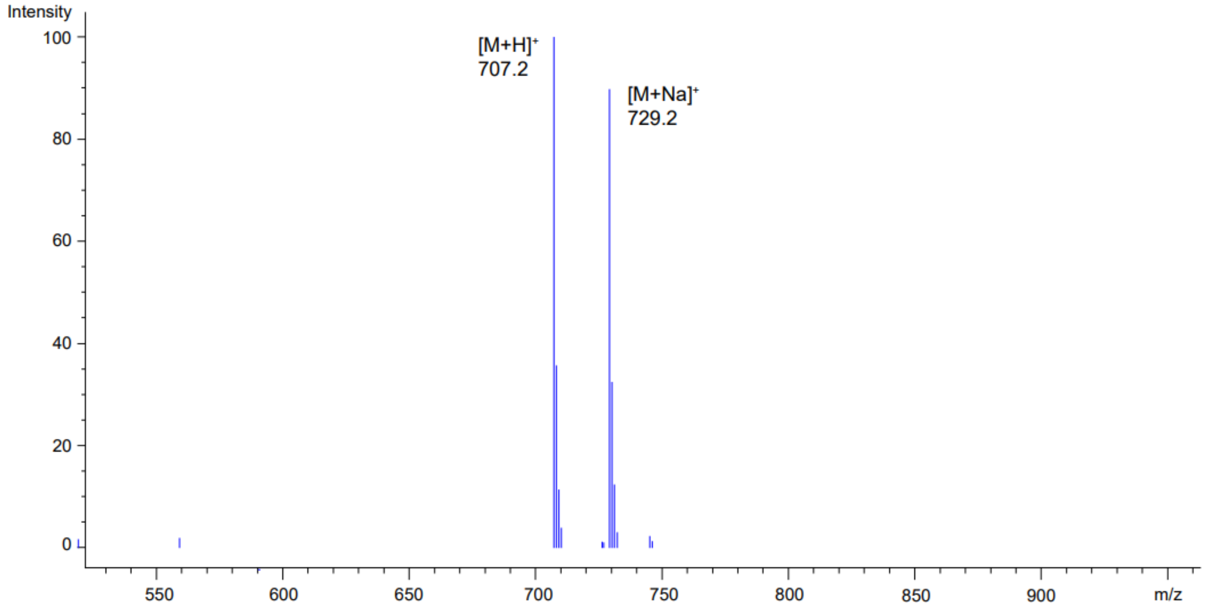

Figure S56. RP-HPLC trace at 220 nm (5-70% MeCN over 21 min) and ESI-MS spectrum of purified peptide starting material **5a**. m/z 707.2 and 729.2 correspond to $[M+H]^+$ and $[M+Na]^+$, respectively.

Ac-MGRFTINP-NH₂ (6a)

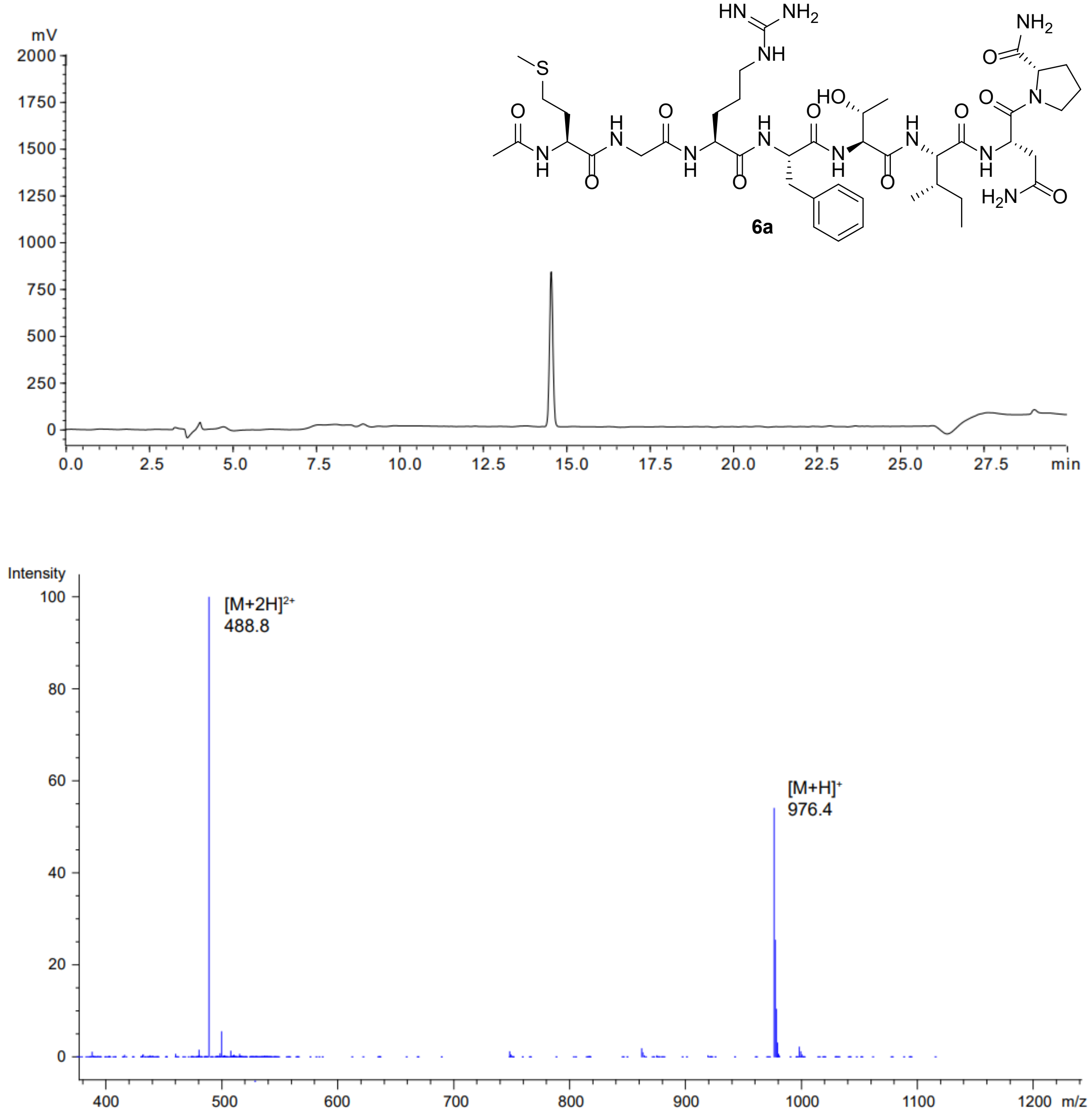

Figure S57. RP-HPLC trace at 220 nm (5-70% MeCN over 21 min) and ESI-MS spectrum of purified peptide starting material **6a**. m/z 488.8 and 976.4 correspond to $[M+2H]^{2+}$ and $[M+H]^{+}$, respectively.

Ac-RPKPQQFFGLM-NH_2 (7a)

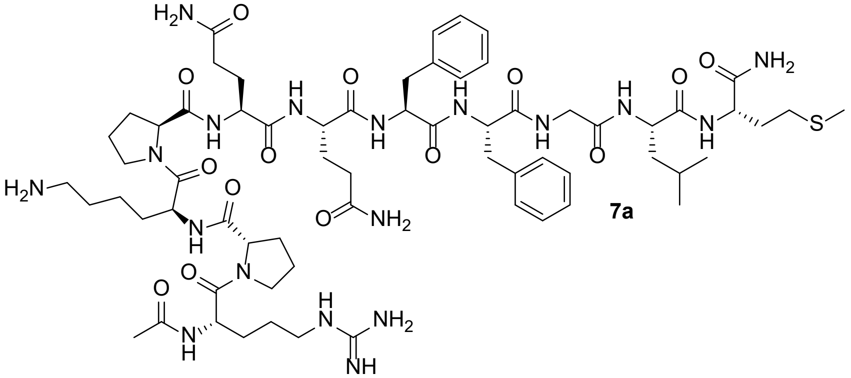

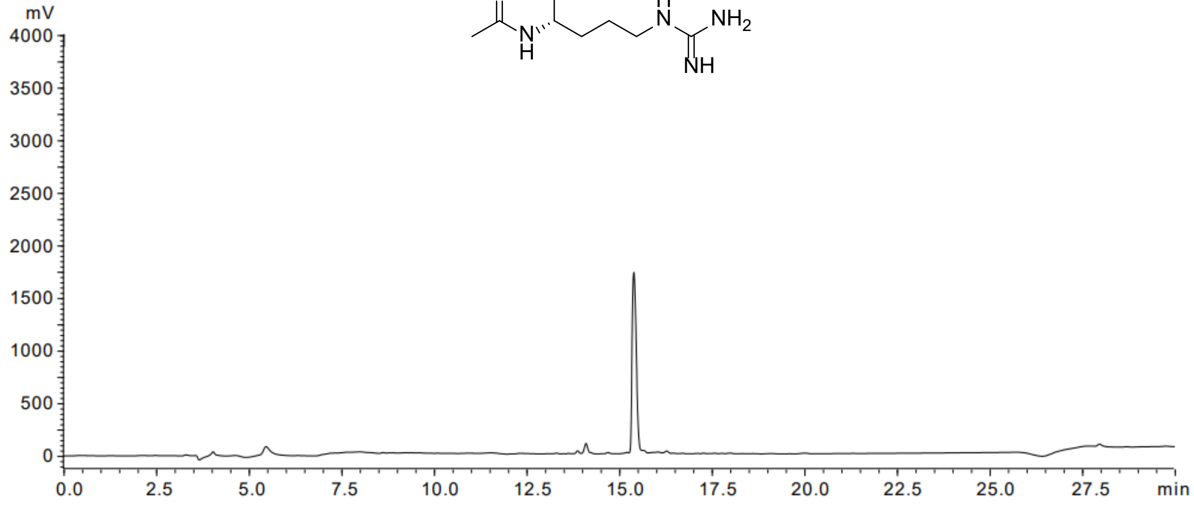

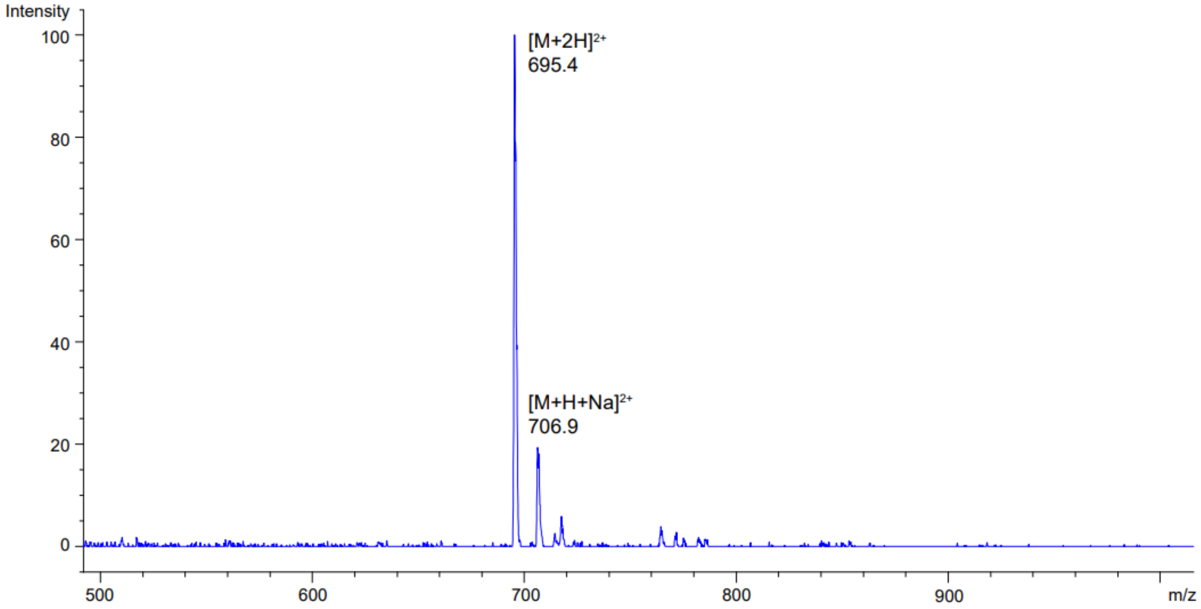

Figure S58. RP-HPLC trace at 220 nm (5-70% MeCN over 21 min) and ESI-MS spectrum of purified peptide starting material **7a**. m/z 695.4 and 706.9 correspond to $[M+2H]^{2+}$ and $[M+H+Na]^{2+}$, respectively.

Methionine sulfoximine peptides

Methionine sulfoximine peptide 1b

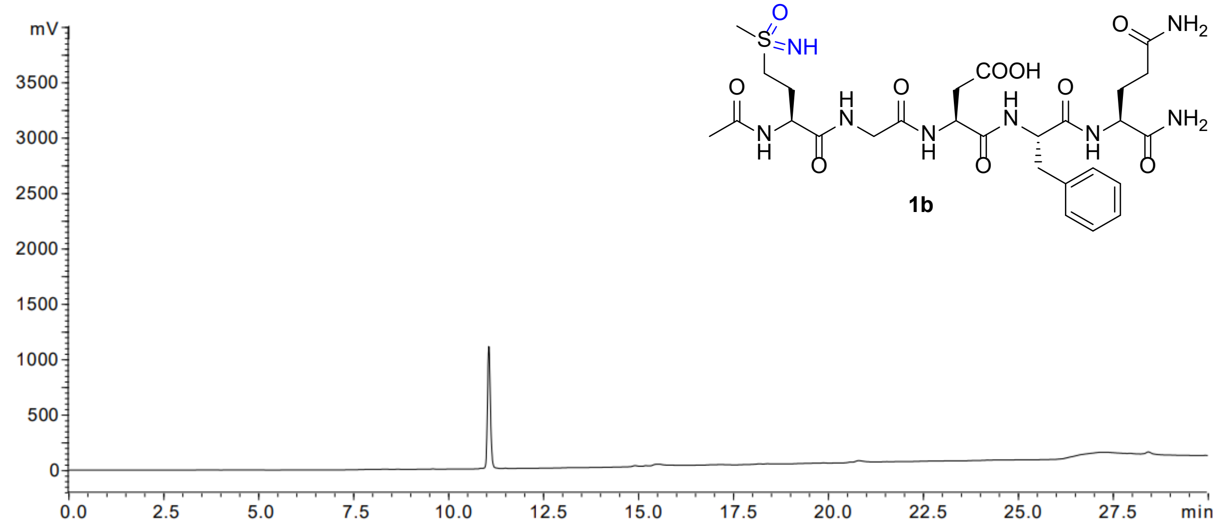

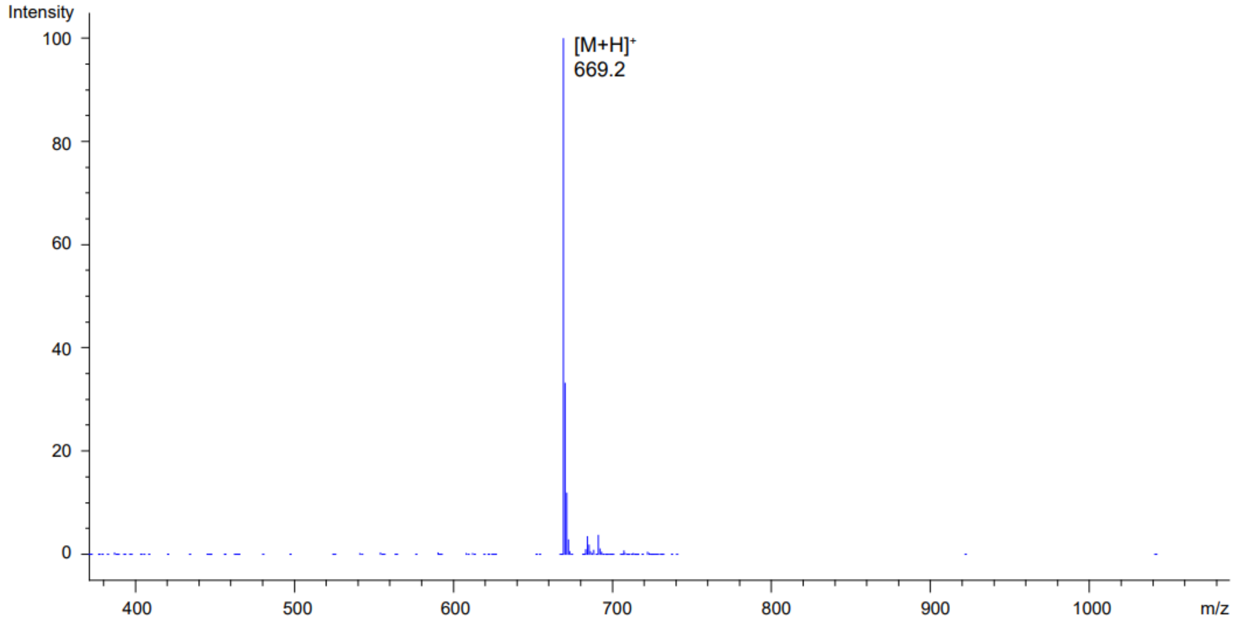

Figure S59. RP-HPLC trace at 220 nm (5-70% MeCN over 21 min) and ESI-MS spectrum of purified methionine sulfoximine peptide **1b**. m/z 669.2 correspond to $[M+H]^+$.

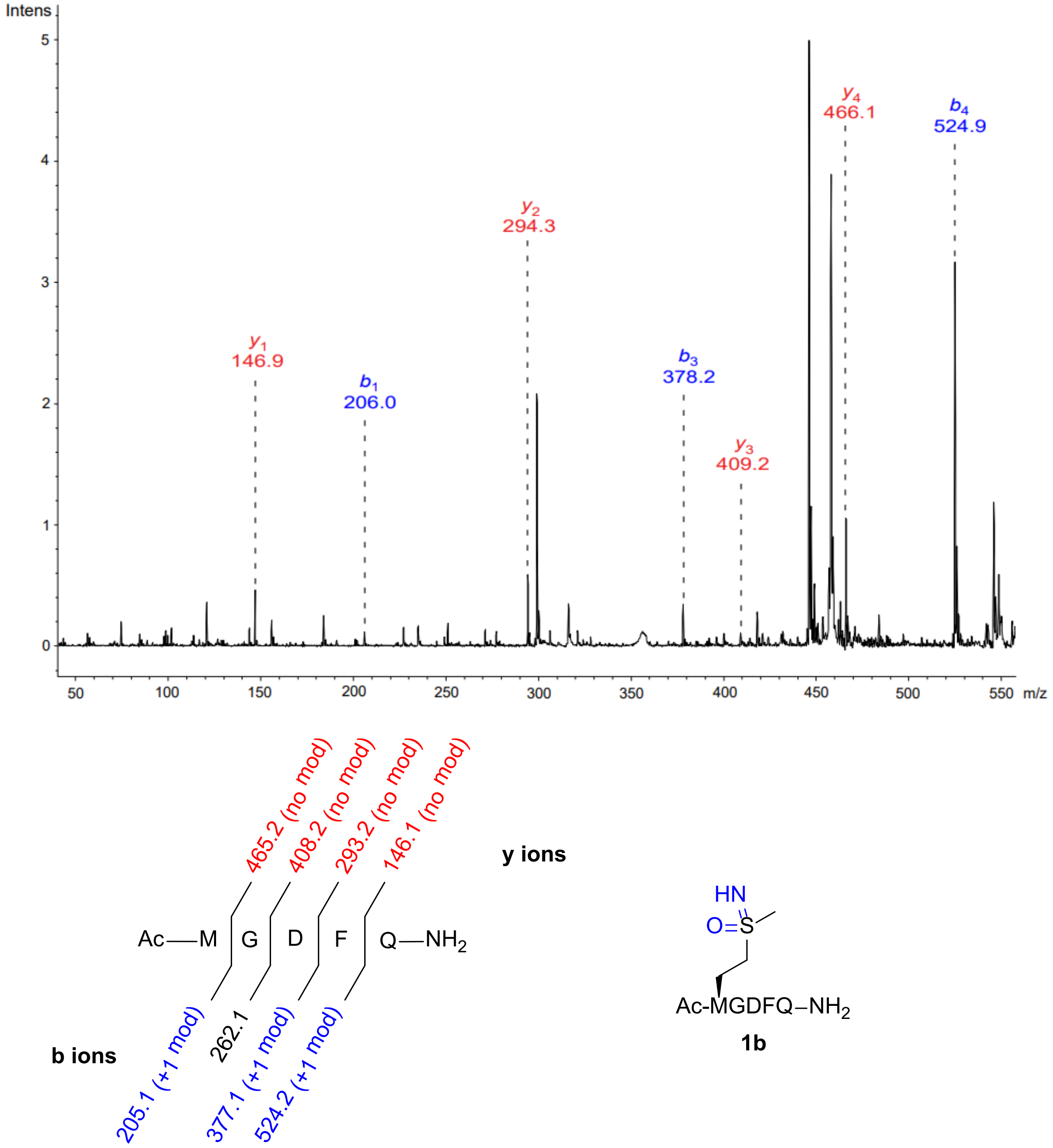

Figure S60. MALDI-MS/MS spectrum and the sequence and fragmentation ladder of methionine sulfoximine peptide **1b**. Observed b and y ions are indicated. α-Cyano-4-hydroxy-cinnamic acid (CHCA) was used as a matrix.

Methionine sulfoximine peptide 2b

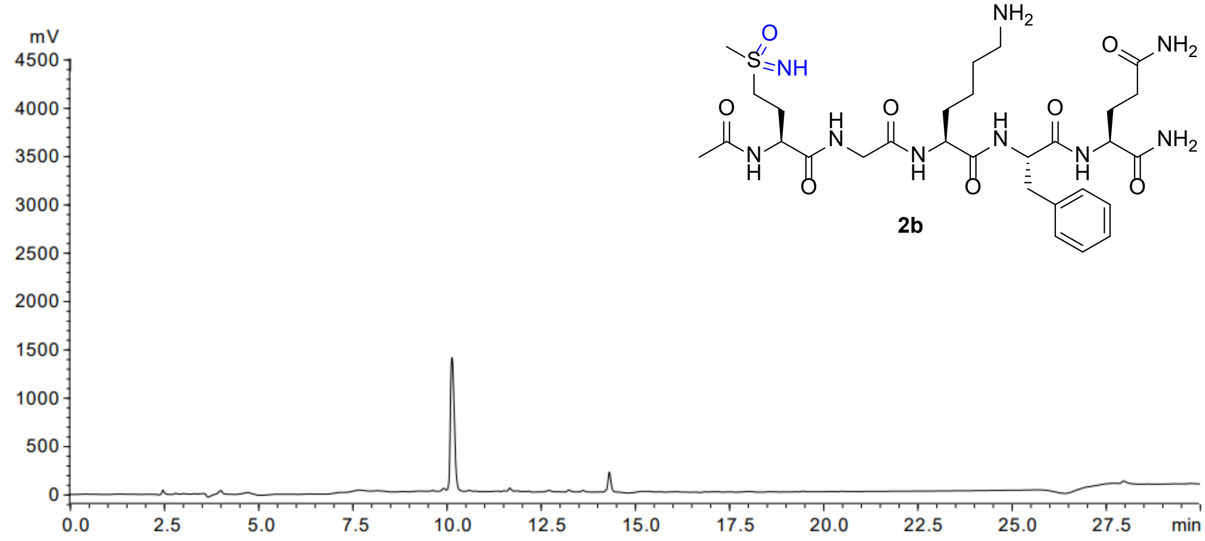

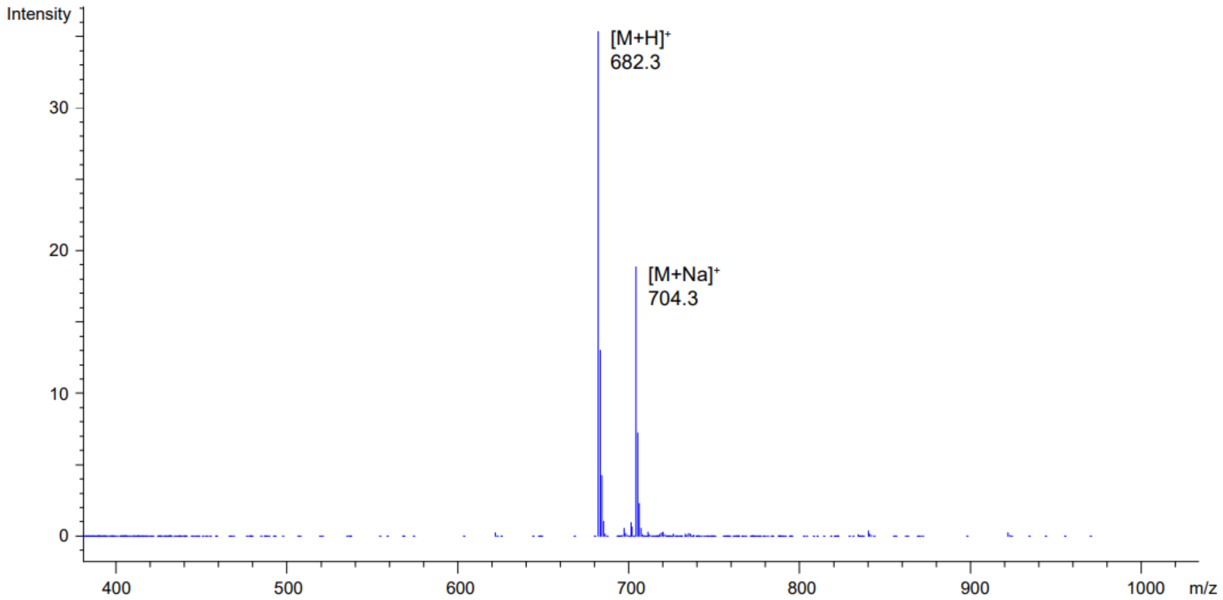

Figure S61. RP-HPLC trace at 220 nm (5-70% MeCN over 21 min) and ESI-MS spectrum of purified methionine sulfoximine peptide **2b**. m/z 682.3 and 704.3 correspond to $[M+H]^+$ and $[M+Na]^+$, respectively.

Methionine sulfoximine peptide 3b

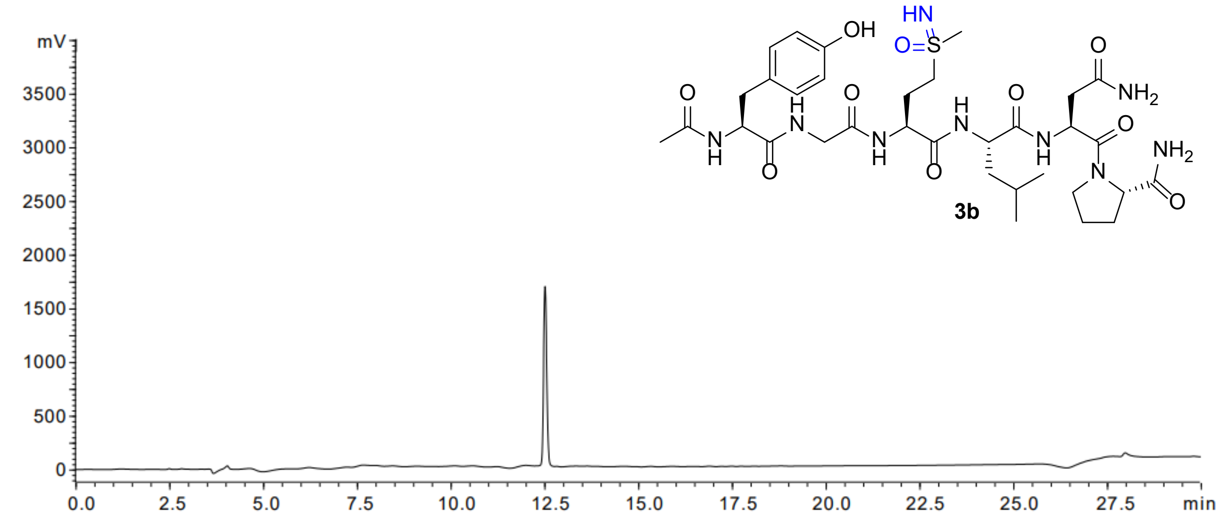

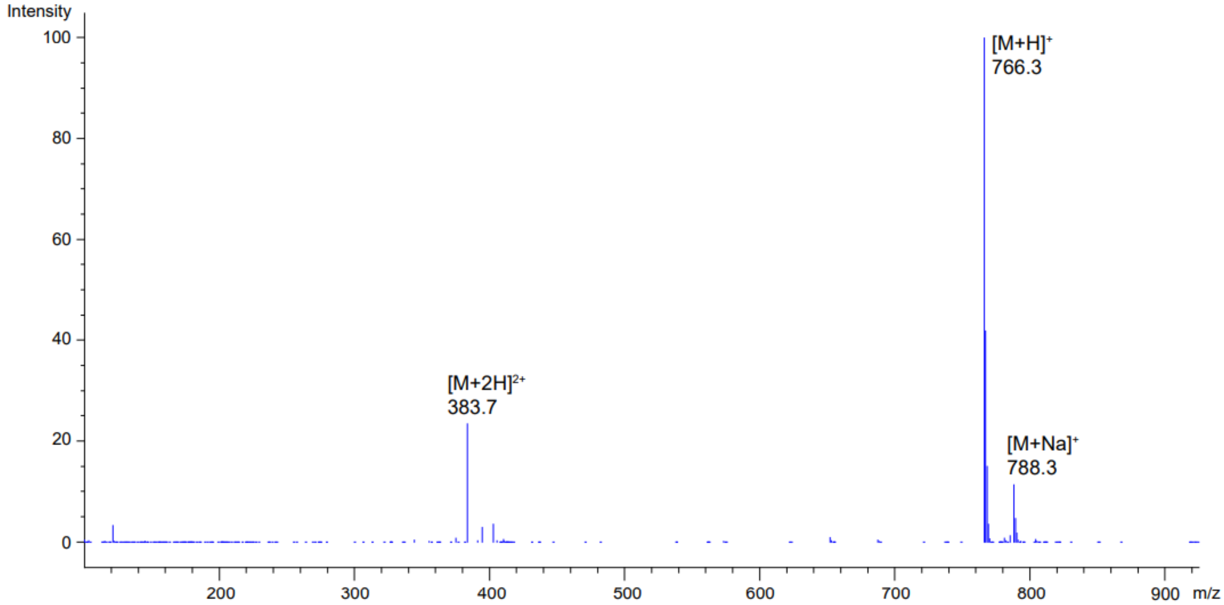

Figure S62. RP-HPLC trace at 220 nm (5-70% MeCN over 21 min) and ESI-MS spectrum of purified methionine sulfoximine peptide **3b**. m/z 383.7, 766.3 and 788.3 correspond to $[M+2H]^{2+}$, $[M+H]^{+}$ and $[M+Na]^{+}$, respectively.

Methionine sulfoximine peptide 4b

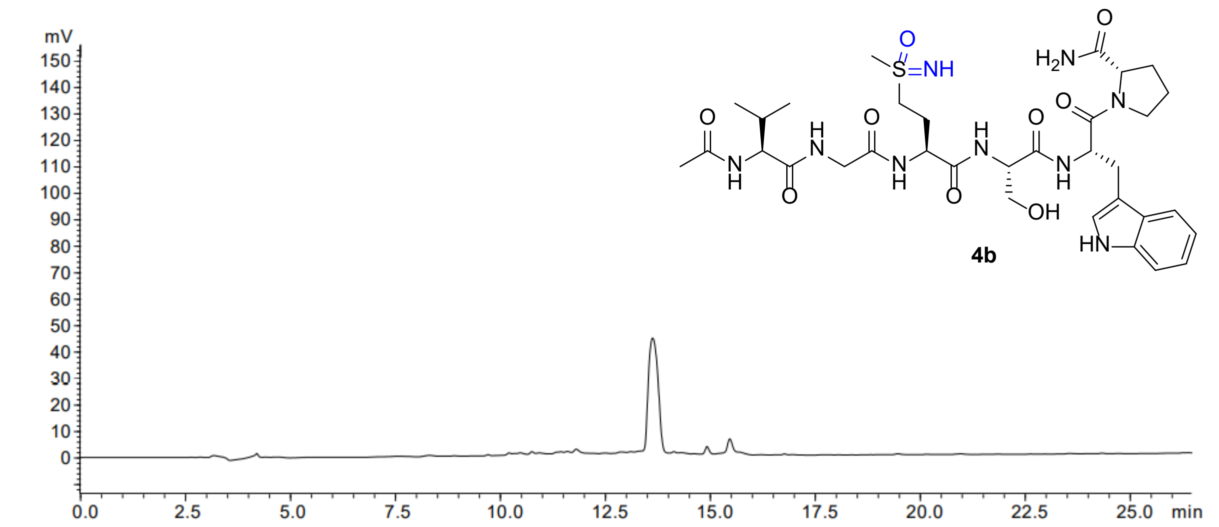

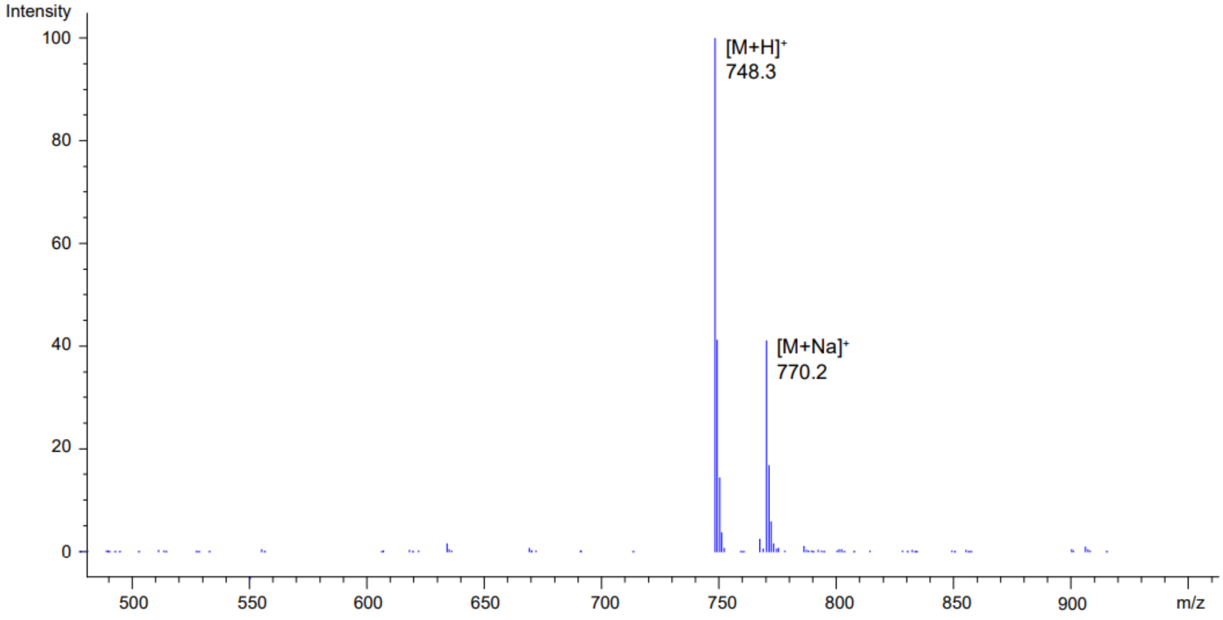

Figure S63. RP-HPLC trace at 280 nm (5-70% MeCN over 21 min) and ESI-MS spectrum of purified methionine sulfoximine peptide **4b**. m/z 748.3 and 770.2 correspond to $[M+H]^+$ and $[M+Na]^+$, respectively.

Methionine sulfoximine peptide 5b

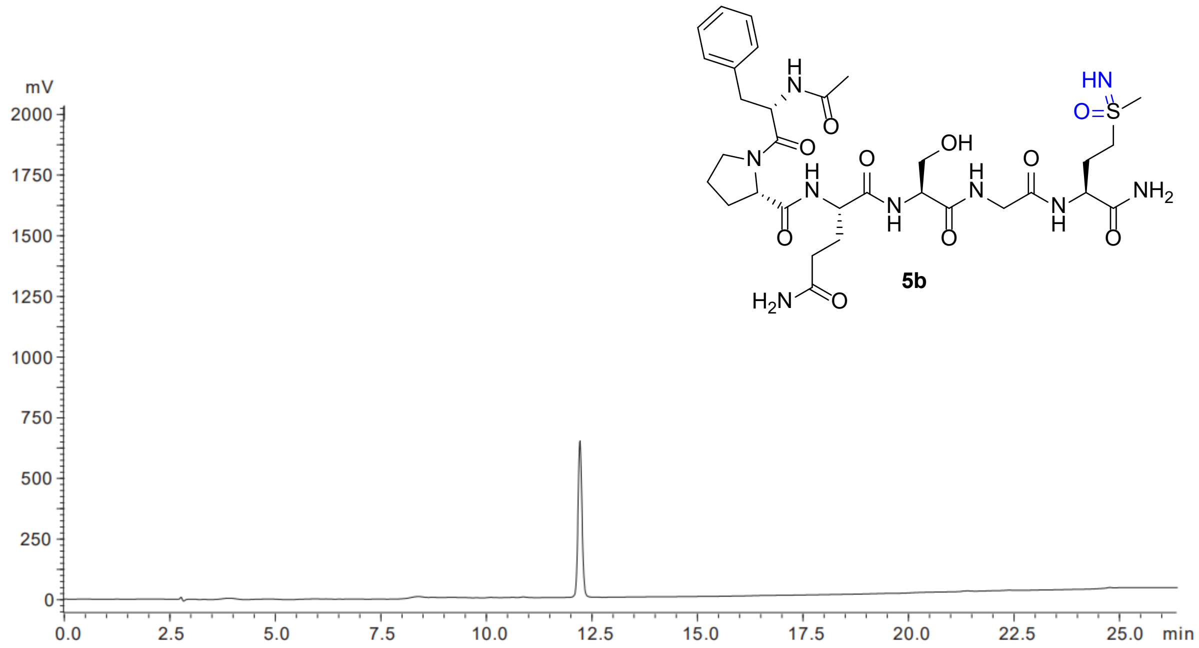

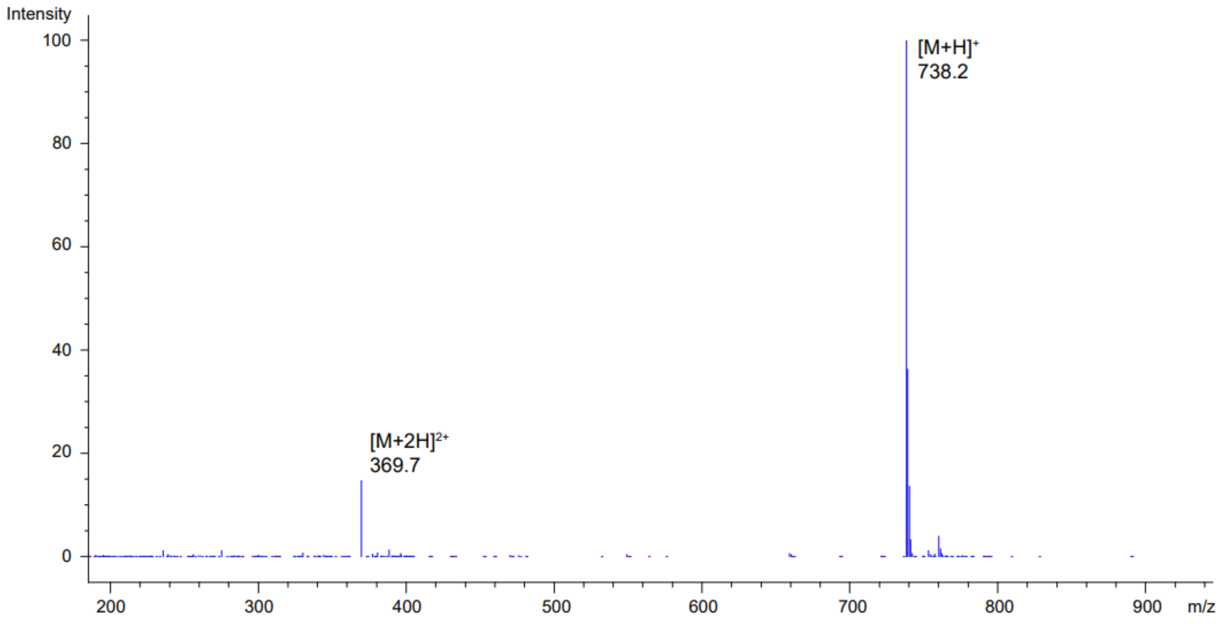

Figure S64. RP-HPLC trace at 220 nm (5-70% MeCN over 21 min) and ESI-MS spectrum of purified methionine sulfoximine peptide **5b**. m/z 369.7 and 738.2 correspond to $[M+2H]^{2+}$ and $[M+H]^{+}$, respectively.

Methionine sulfoximine peptide 6b

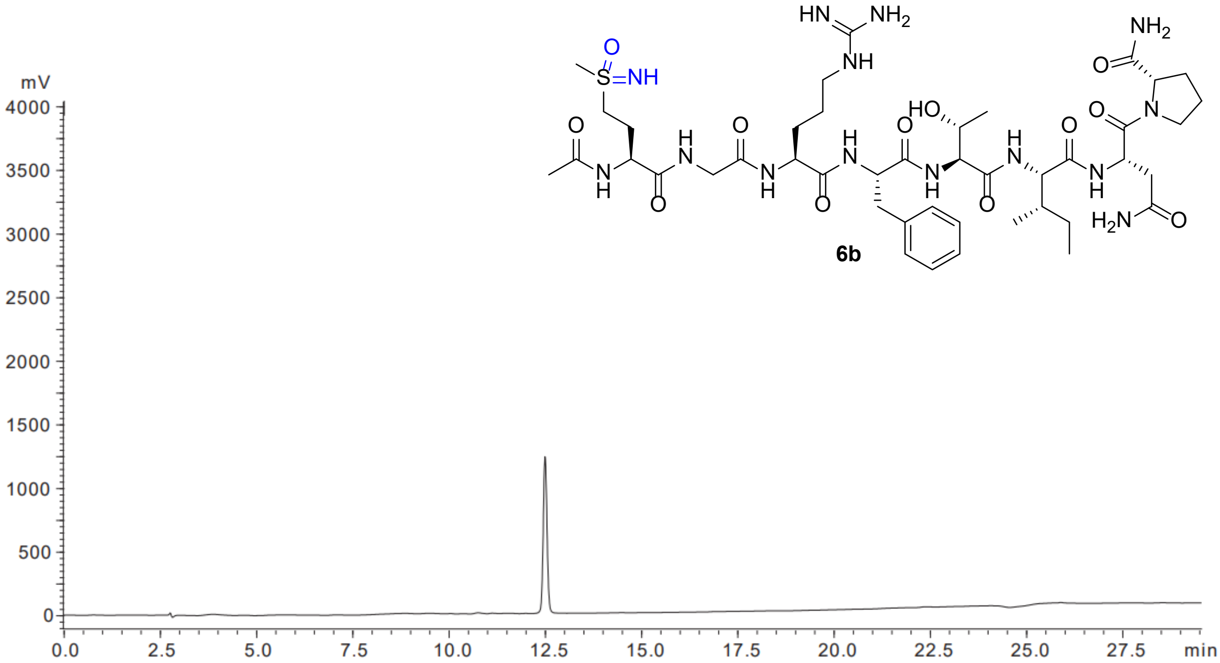

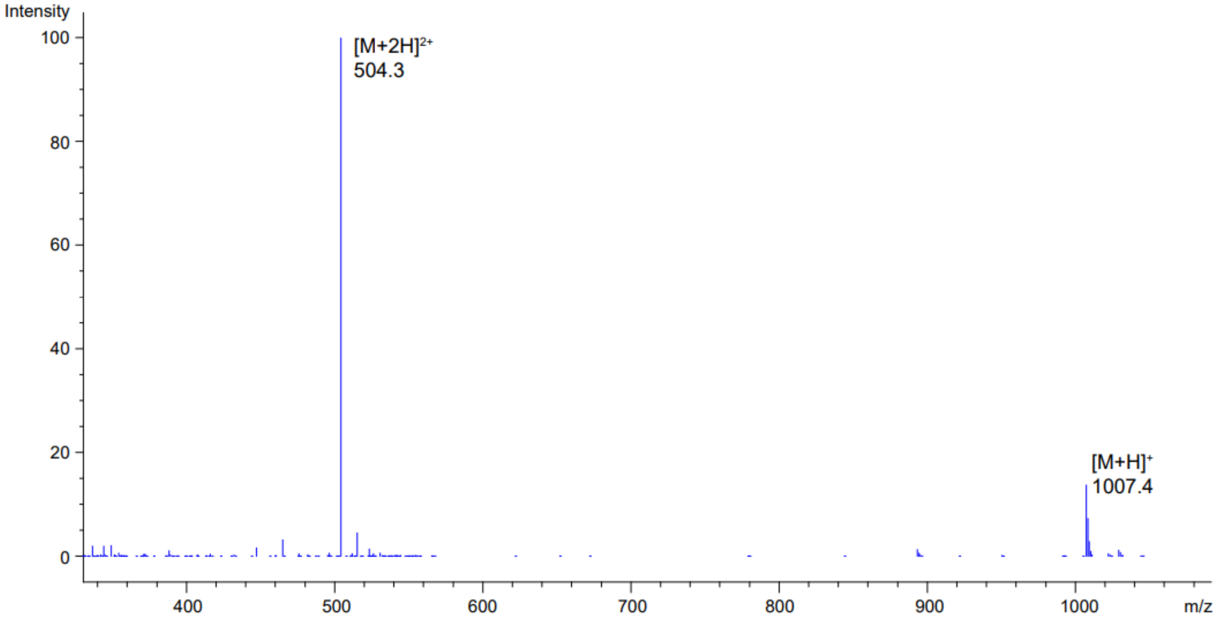

Figure S65. RP-HPLC trace at 220 nm (5-70% MeCN over 21 min) and ESI-MS spectrum of purified methionine sulfoximine peptide **6b**. m/z 504.3 and 1007.4 correspond to $[M+2H]^{2+}$ and $[M+H]^{+}$, respectively.

Methionine sulfoximine peptide 7b

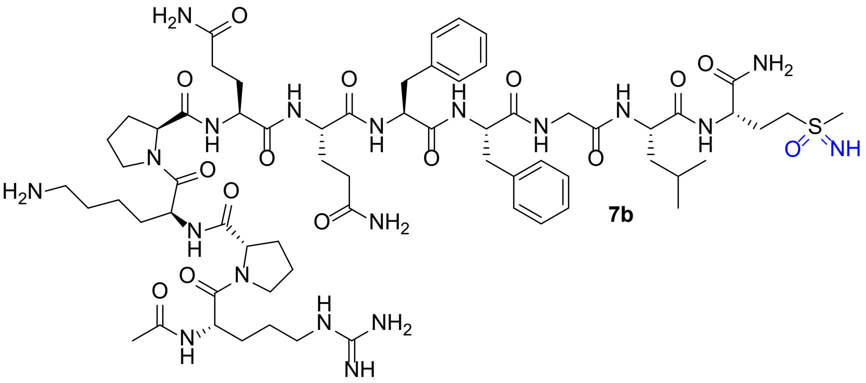

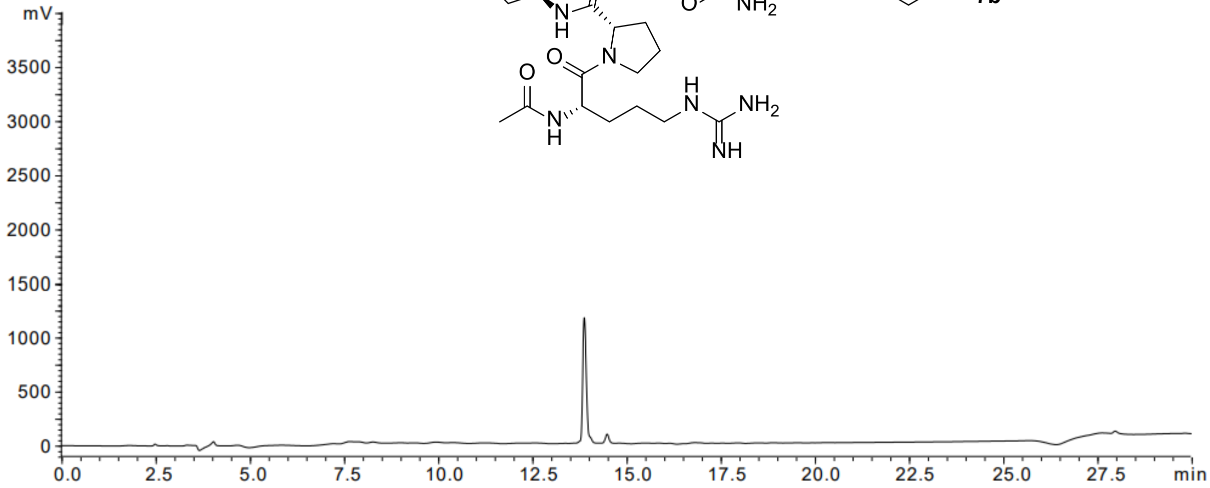

	peak area	purity
Product (13.9 min)	7869169	94%
Impurity (14.5 min)	520151	-

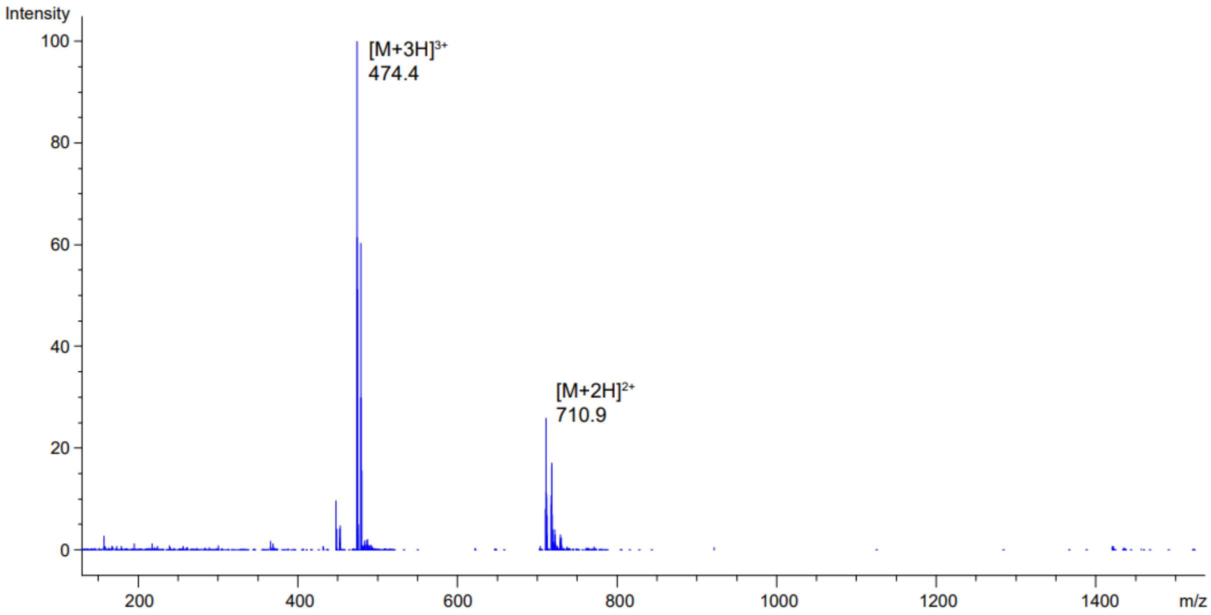

Figure S66. RP-HPLC trace at 220 nm (5-70% MeCN over 21 min) and ESI-MS spectrum of purified methionine sulfoximine peptide **7b**. m/z 474.4 and 710.9 correspond to $[M+3H]^{3+}$ and $[M+2H]^{2+}$, respectively.

Methionine sulfoximine peptide 8b

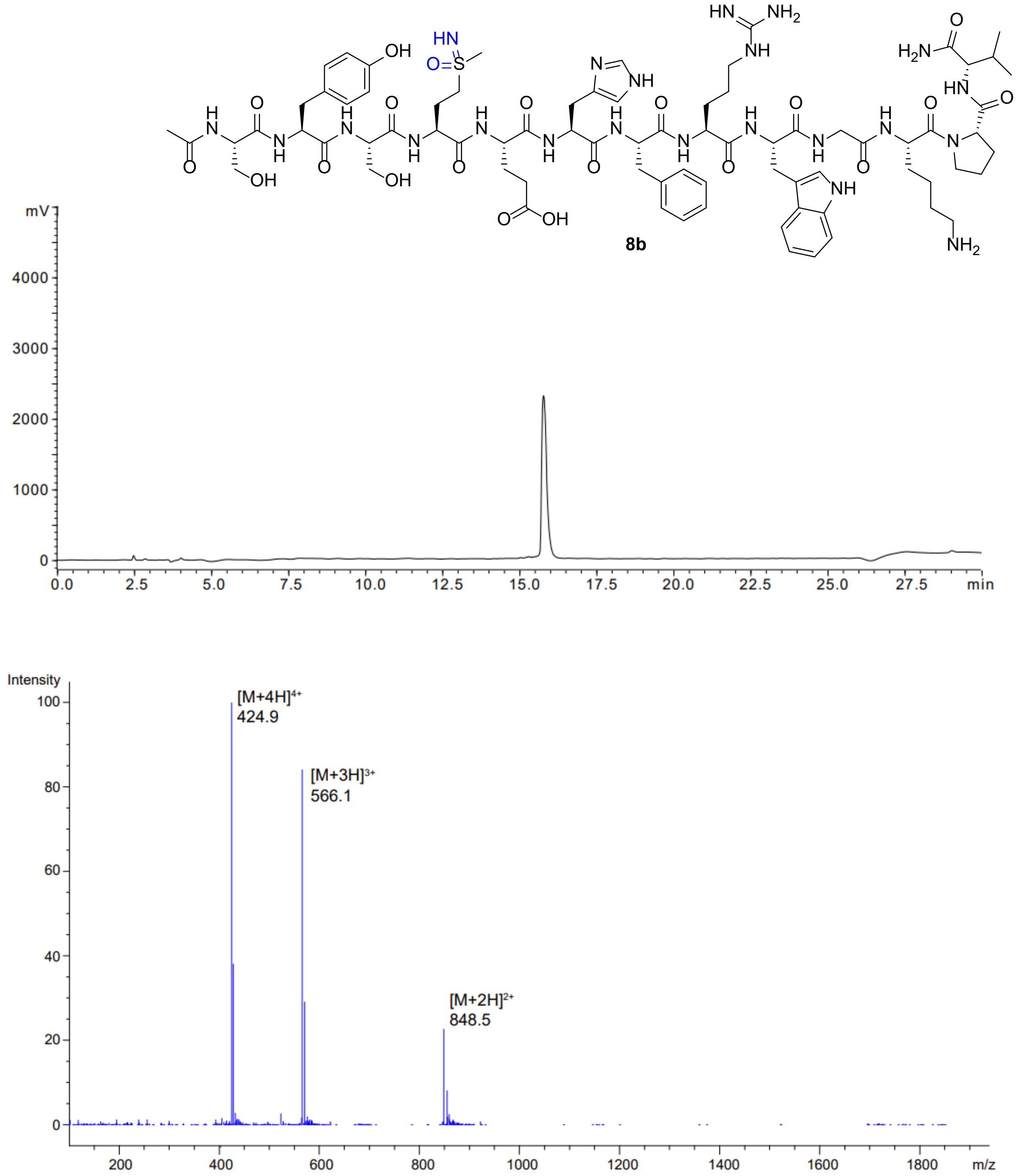

Figure S67. RP-HPLC trace at 220 nm (5-50% MeCN over 21 min) and ESI-MS spectrum of purified methionine sulfoximine peptide **8b**. m/z 424.9, 566.1 and 848.5 correspond to $[M+4H]^{4+}$, $[M+3H]^{3+}$ and $[M+2H]^{2+}$, respectively.

Dabsyl-labeled methionine sulfoximine peptide 4

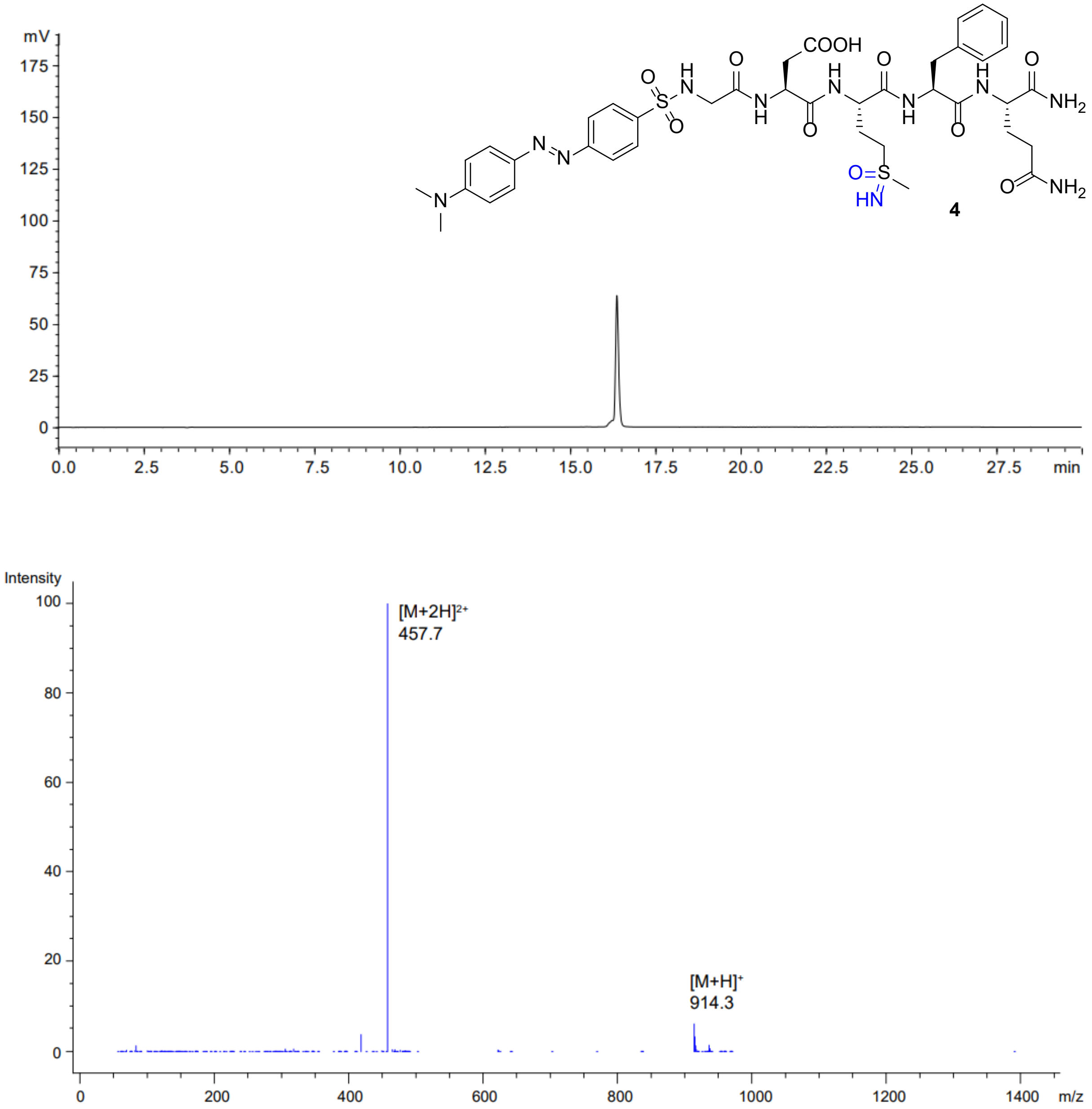

Figure S68. RP-HPLC trace at 500 nm (5-70% MeCN over 21 min) and ESI-MS spectrum of purified dabsyl-labeled methionine sulfoximine peptide **4**. m/z 457.7 and 914.3 correspond to $[M+2H]^{2+}$ and $[M+H]^{+}$, respectively.

N-Arylated peptides

N-Arylated peptide 1d

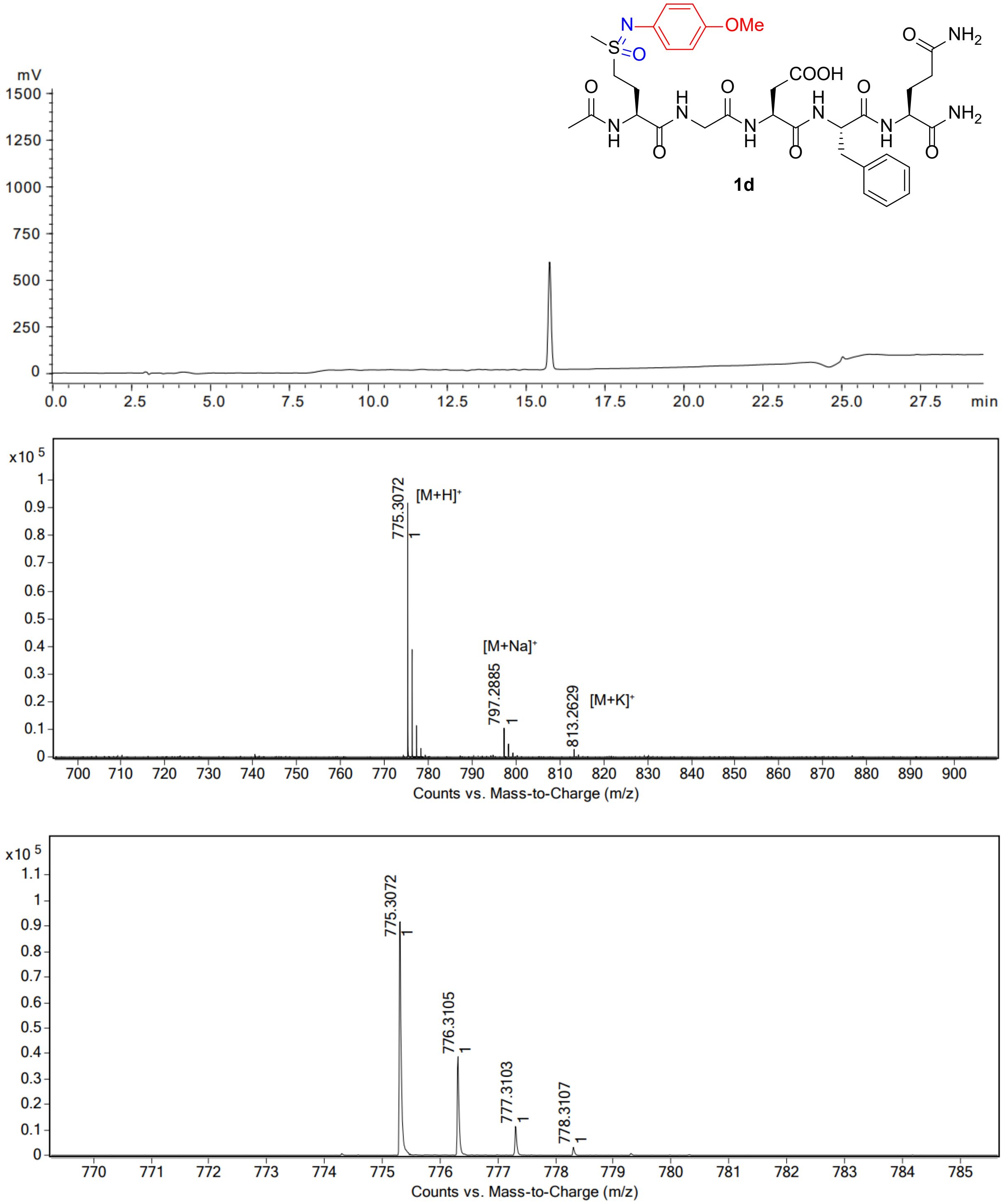

Figure S69. RP-HPLC trace at 220 nm (5-50% MeCN over 18 min) and HRMS spectrum of N-arylated peptide **1d**. HRMS $C_{34}H_{47}N_8O_{11}S^+$ $[M+H]^+$ calcd 775.3080, found 775.3072.

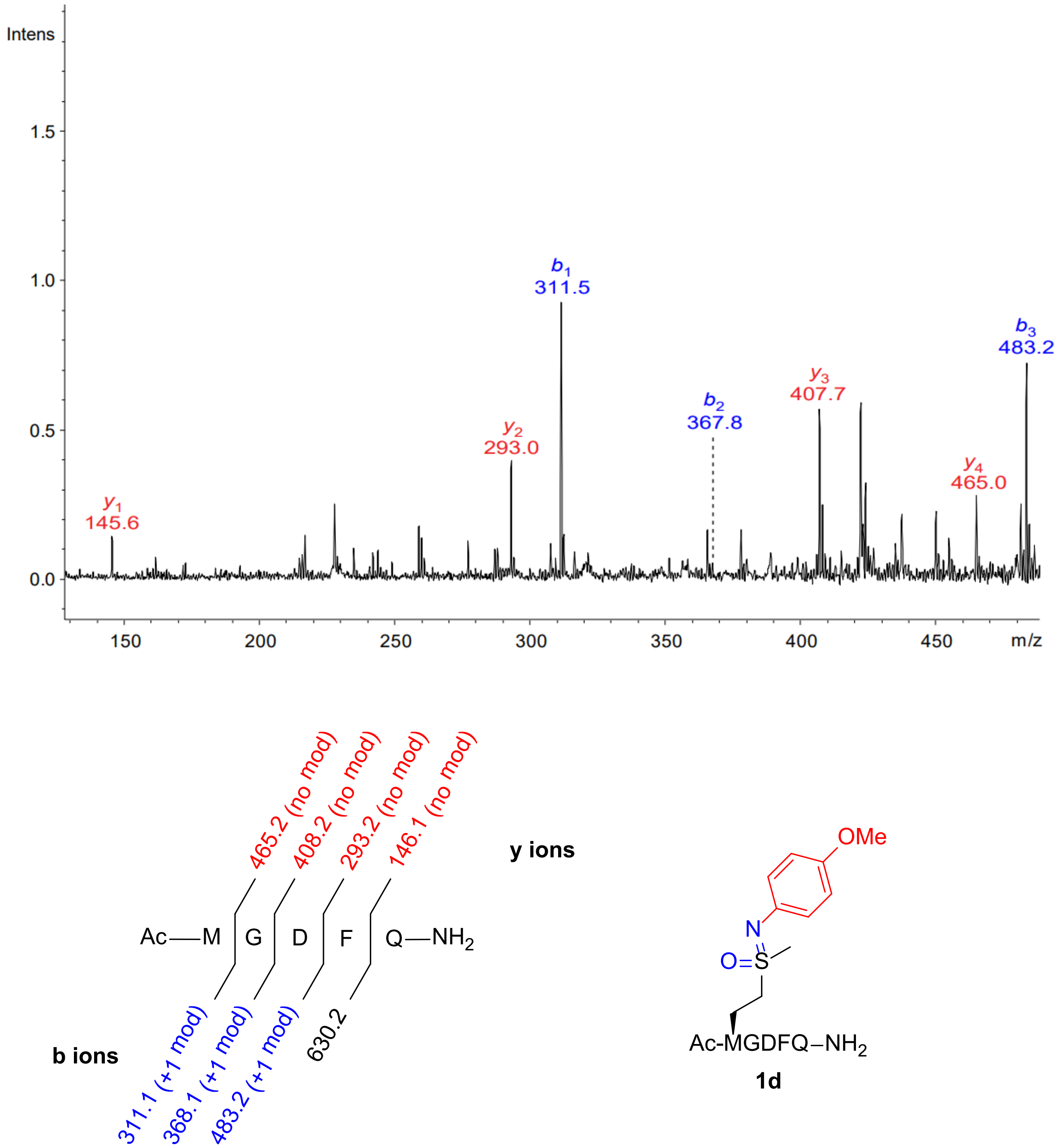

Figure S70. MALDI-MS/MS spectrum and the sequence and fragmentation ladder of N-arylated peptide **1d**. Observed b and y ions are indicated. α-Cyano-4-hydroxy-cinnamic acid (CHCA) was used as a matrix.

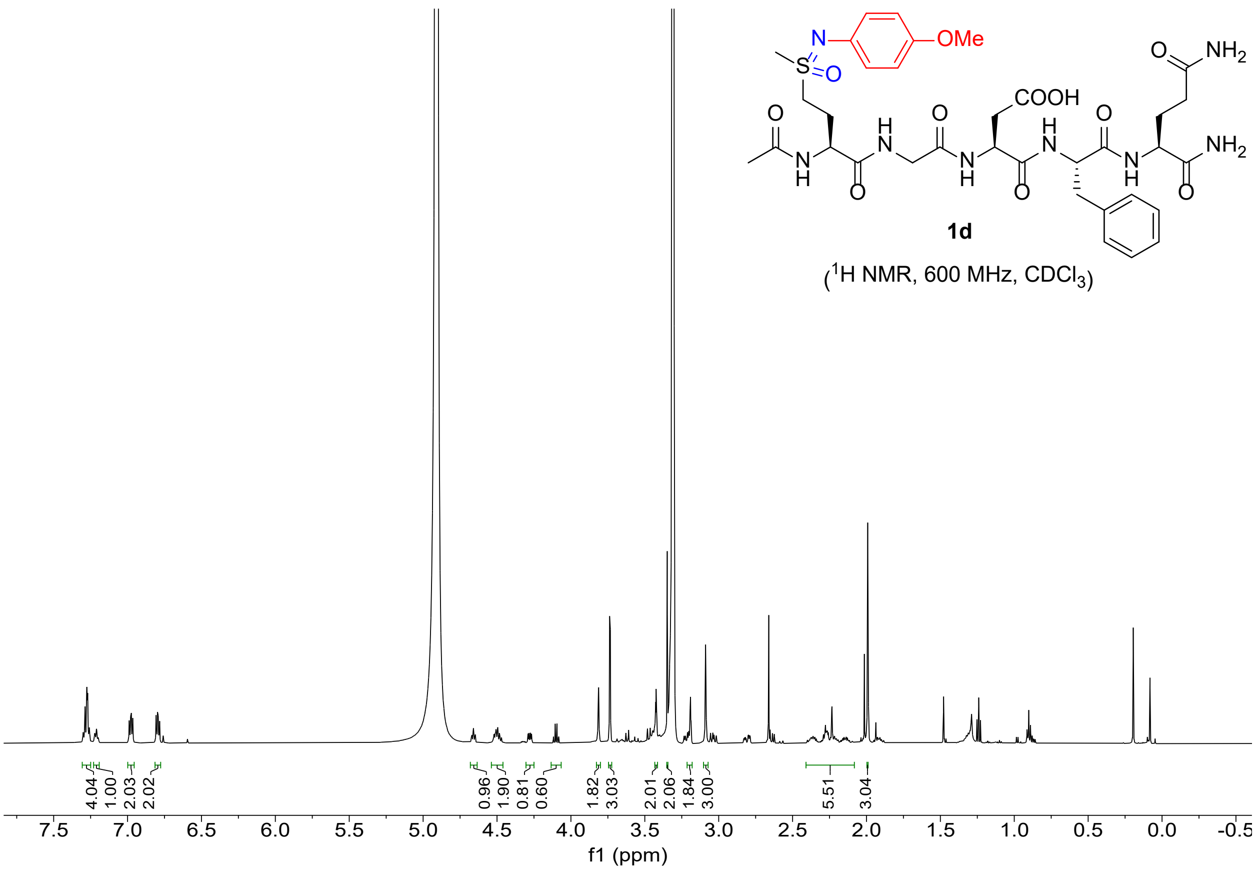

Figure S71. ^{1}H NMR spectrum of N-arylated peptide **1d**.

N-Arylated peptide 7c

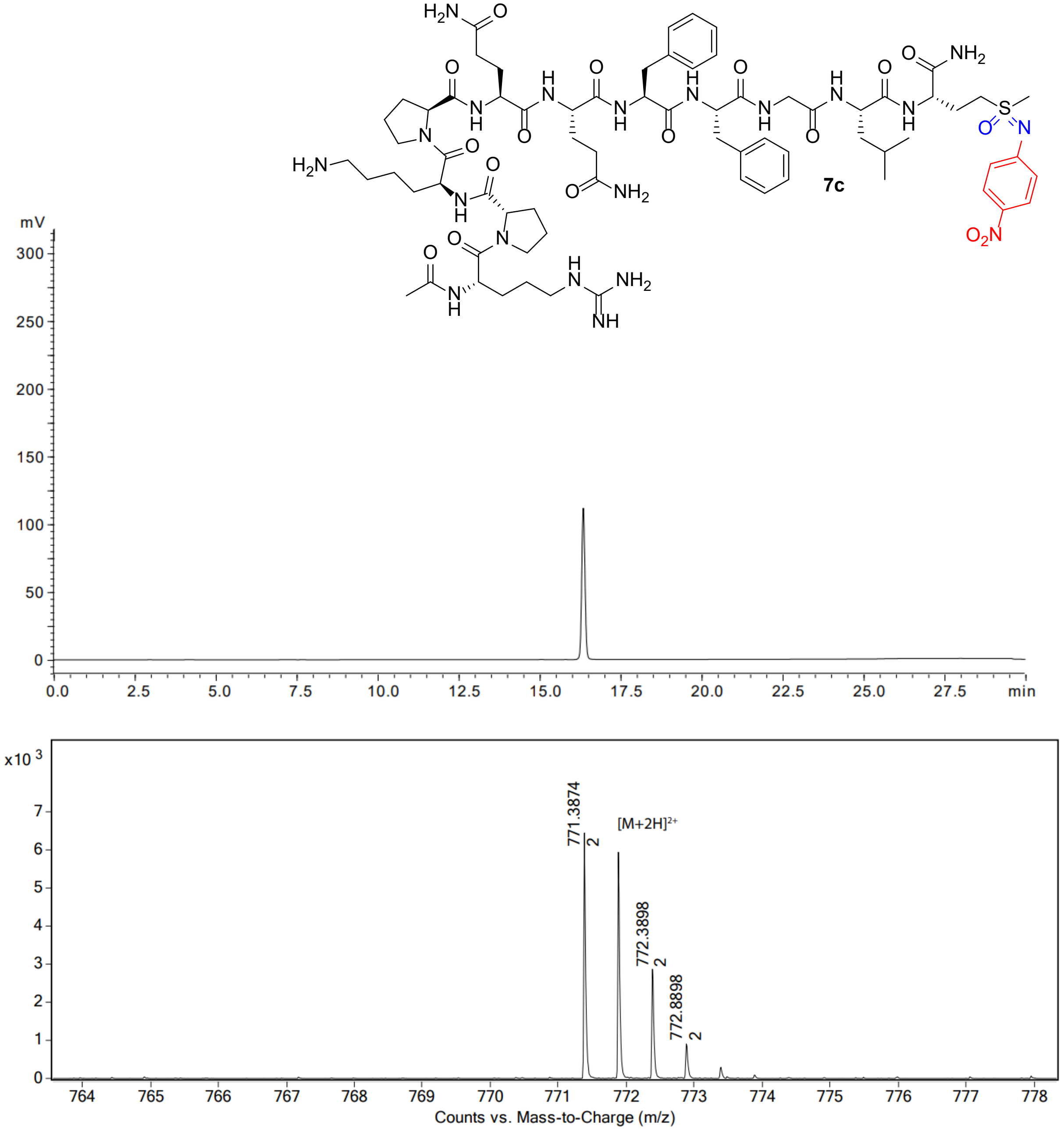

Figure S72. RP-HPLC trace at 350 nm (5-70% MeCN over 21 min) and HRMS spectrum of N-arylated peptide **7c**. HRMS $C_{71}H_{106}N_{20}O_{17}S^{2+}$ $[M+2H]^{+}$ calcd 771.3878, found 771.3874.

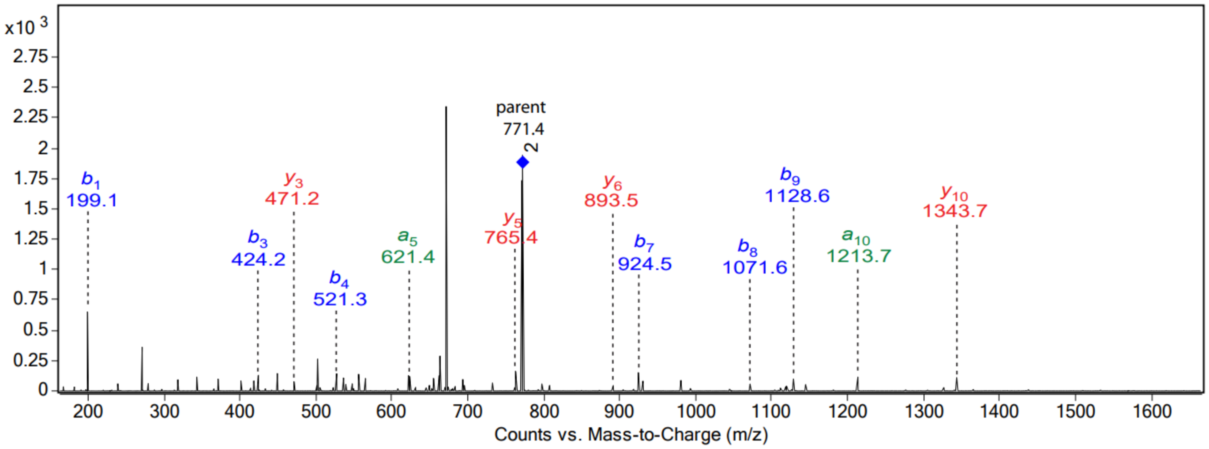

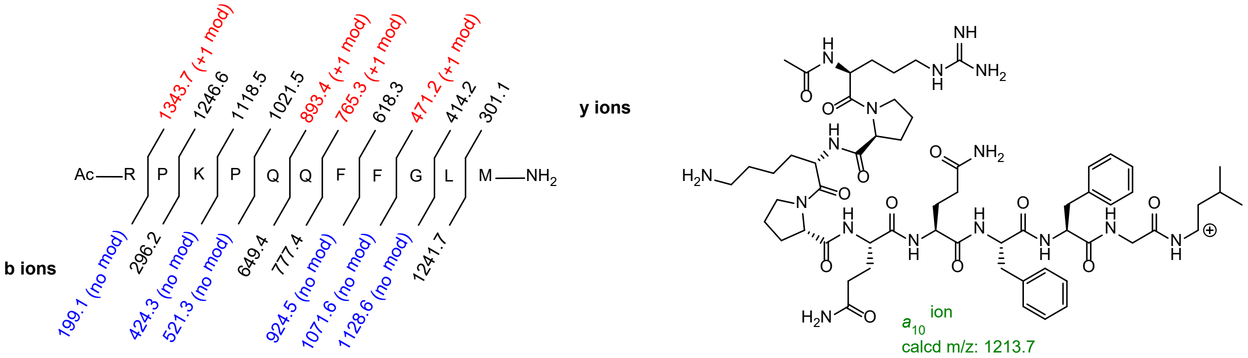

Figure S73. LC-MS/MS spectrum and the sequence and fragmentation ladder of N-arylated peptide **7c**. Observed b, y and a ions are indicated.

_N_-Arylated peptide 8c

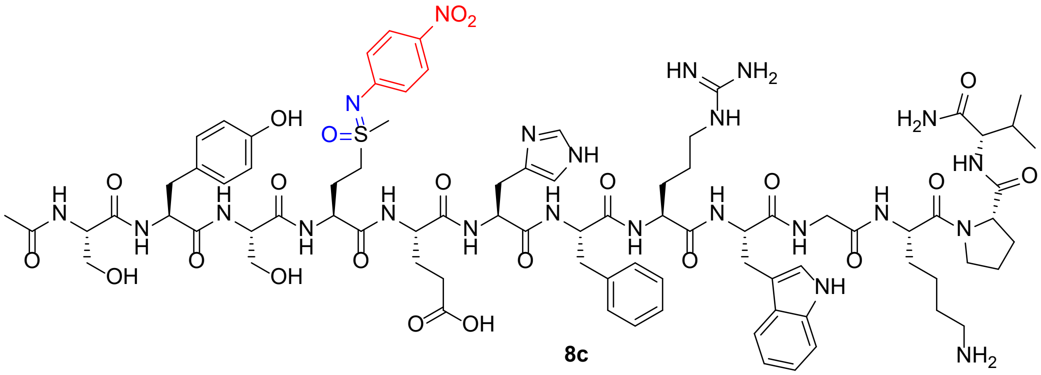

8c

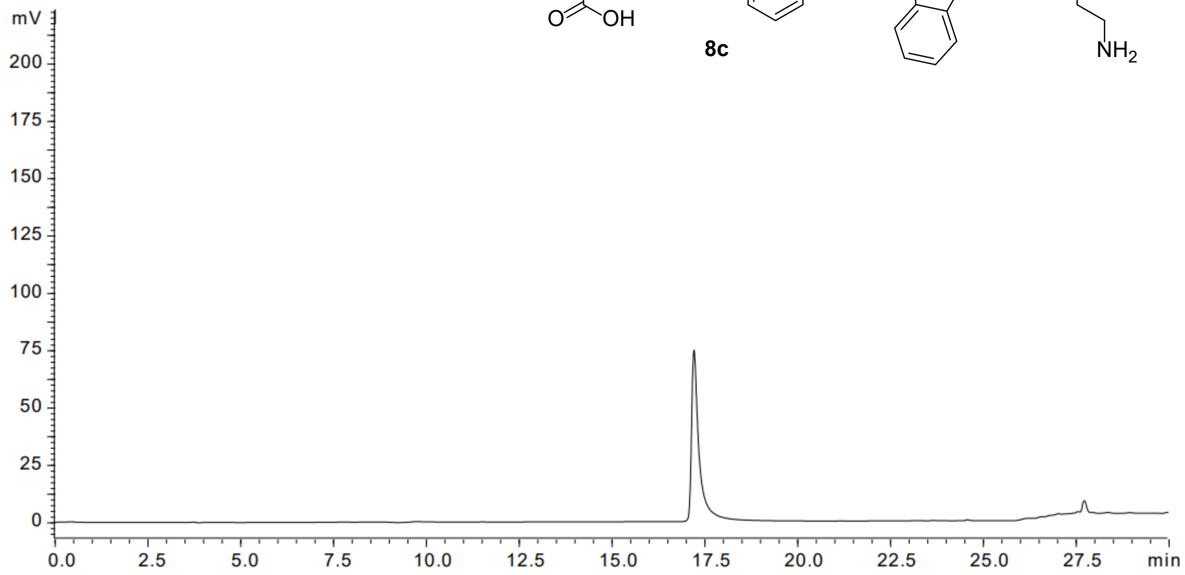

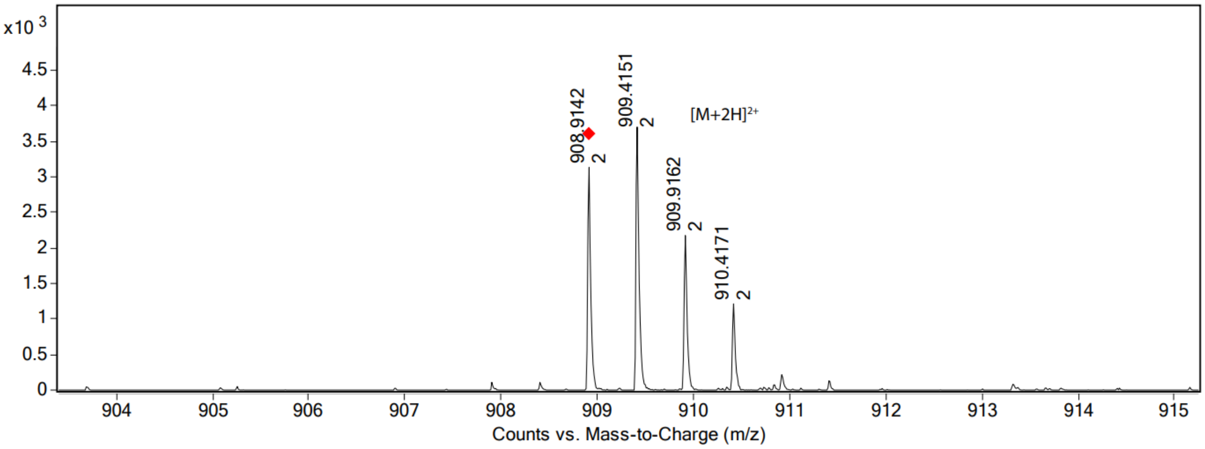

Figure S74. RP-HPLC trace at 350 nm (5-70% MeCN over 21 min) and HRMS spectrum of N-arylated peptide **8c**. HRMS $C_{83}H_{115}N_{23}O_{22}S^{2+}$ $[M+2H]^+$ calcd 908.9149, found 908.9142.

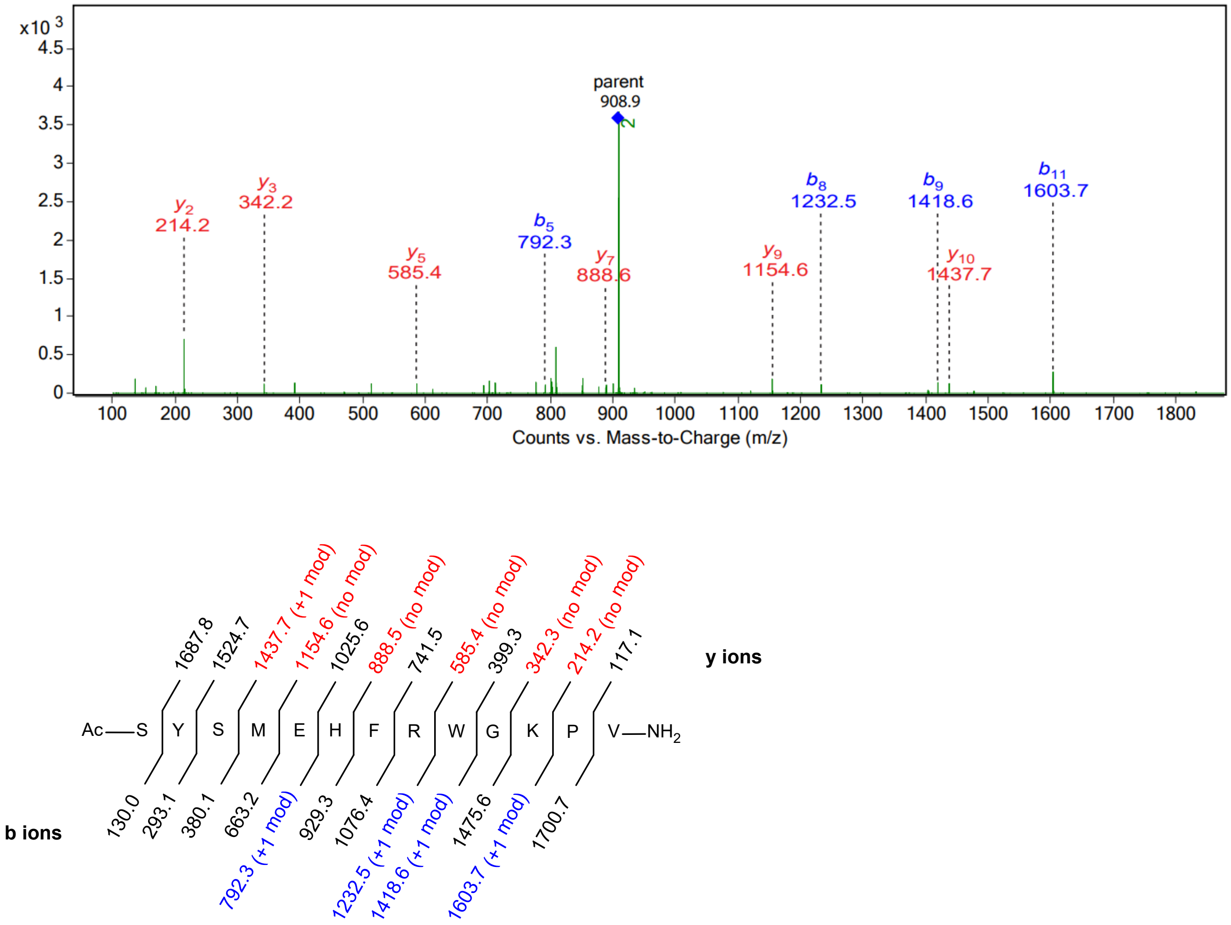

Figure S75. LC-MS/MS spectrum and the sequence and fragmentation ladder of N-arylated peptide **8c**. Observed b and y ions are indicated.

Small molecule reagents

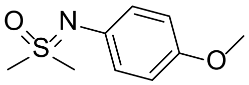

S2

(^{1}H NMR, 600 MHz, $CDCl_3$)

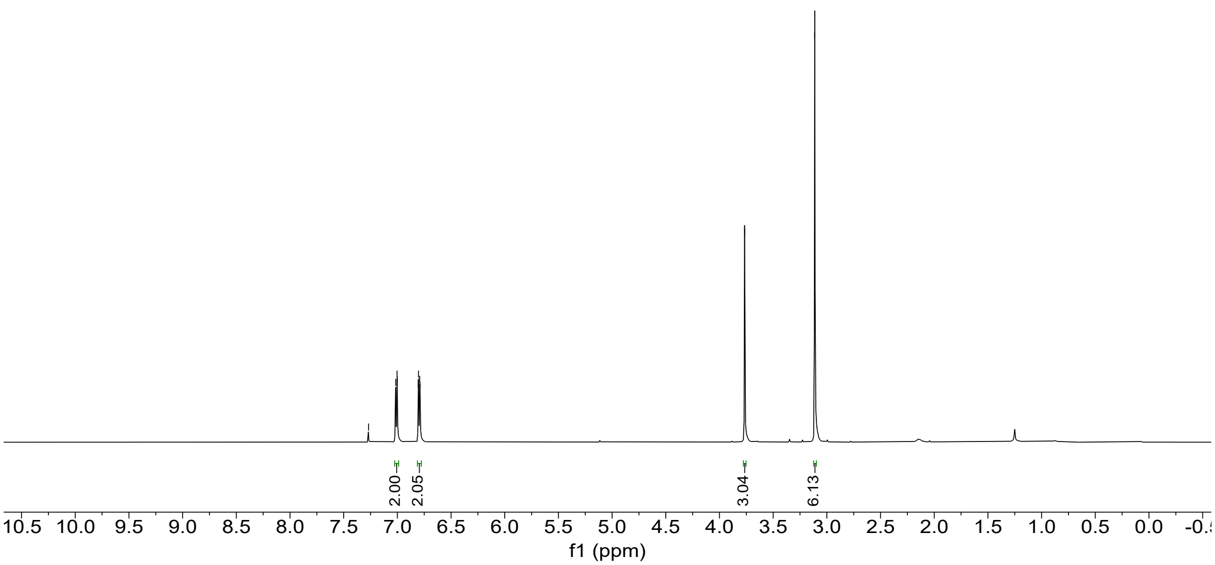

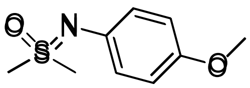

S2

(^{13}C NMR, 151 MHz, $CDCl_3$)

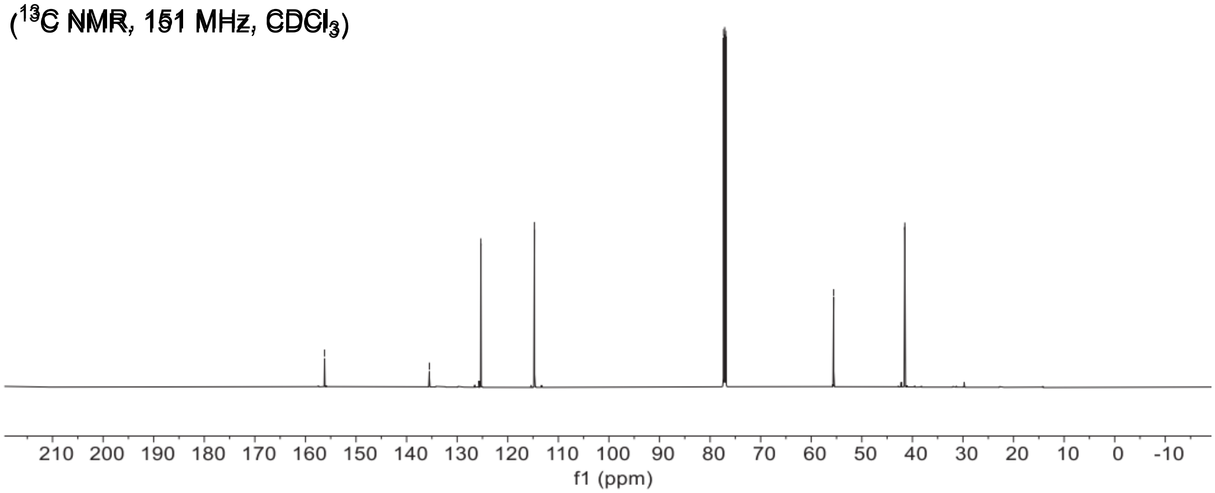

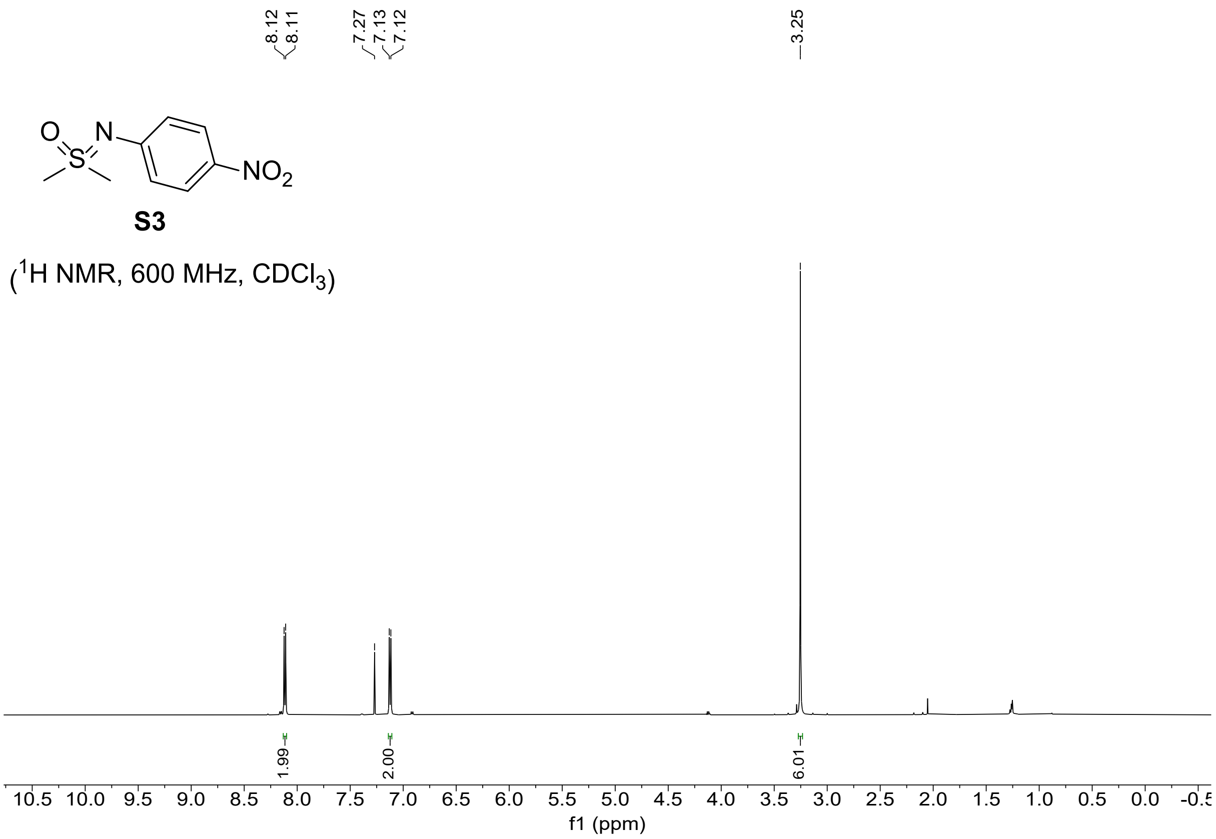
8.12
8.11
7.27
7.13
7.12
3.25
NO2
S3
(1H NMR, 600 MHz, CDCl3)
1.99
2.00
6.01
10.5 10.0 9.5 9.0 8.5 8.0 7.5 7.0 6.5 6.0 5.5 5.0 4.5 4.0 3.5 3.0 2.5 2.0 1.5 1.0 0.5 0.0 -0.5
f1 (ppm)

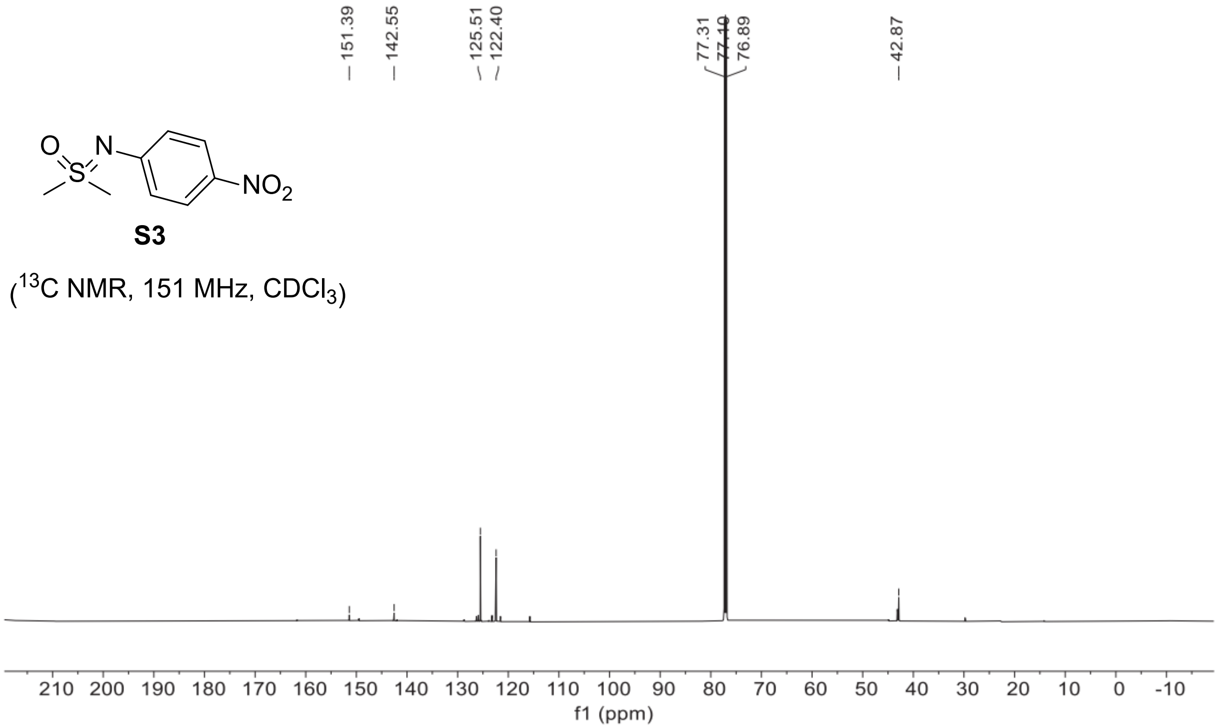
151.39
142.55
125.51
122.40
77.31
77.10
76.89
42.87
NO2
S3
(13C NMR, 151 MHz, CDCl3)
210 200 190 180 170 160 150 140 130 120 110 100 90 80 70 60 50 40 30 20 10 0 -10
f1 (ppm)

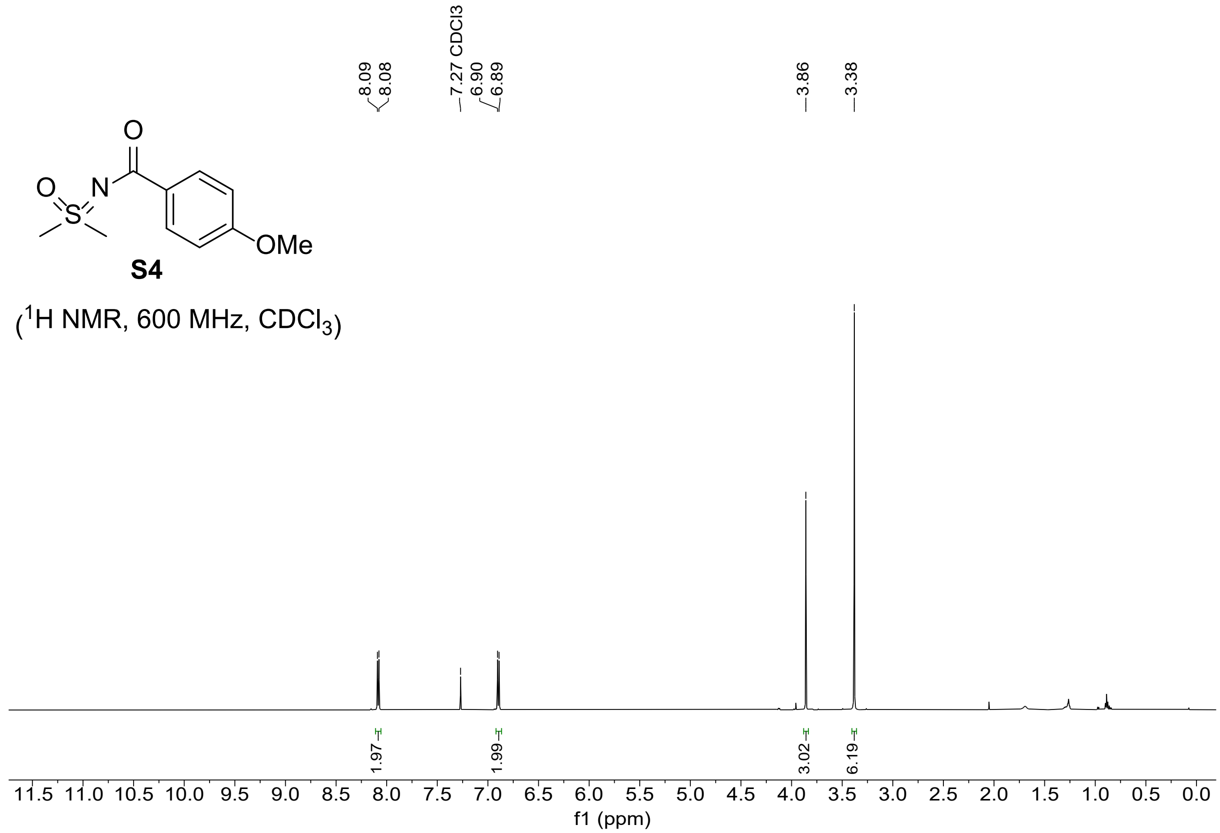
8.09
8.08
7.27 CDCl3
6.90
6.89
3.86
3.38
O
N
O
S
OMe
S4
(1H NMR, 600 MHz, CDCl3)
1.97
1.99
3.02
6.19
11.5 11.0 10.5 10.0 9.5 9.0 8.5 8.0 7.5 7.0 6.5 6.0 5.5 5.0 4.5 4.0 3.5 3.0 2.5 2.0 1.5 1.0 0.5 0.0
f1 (ppm)

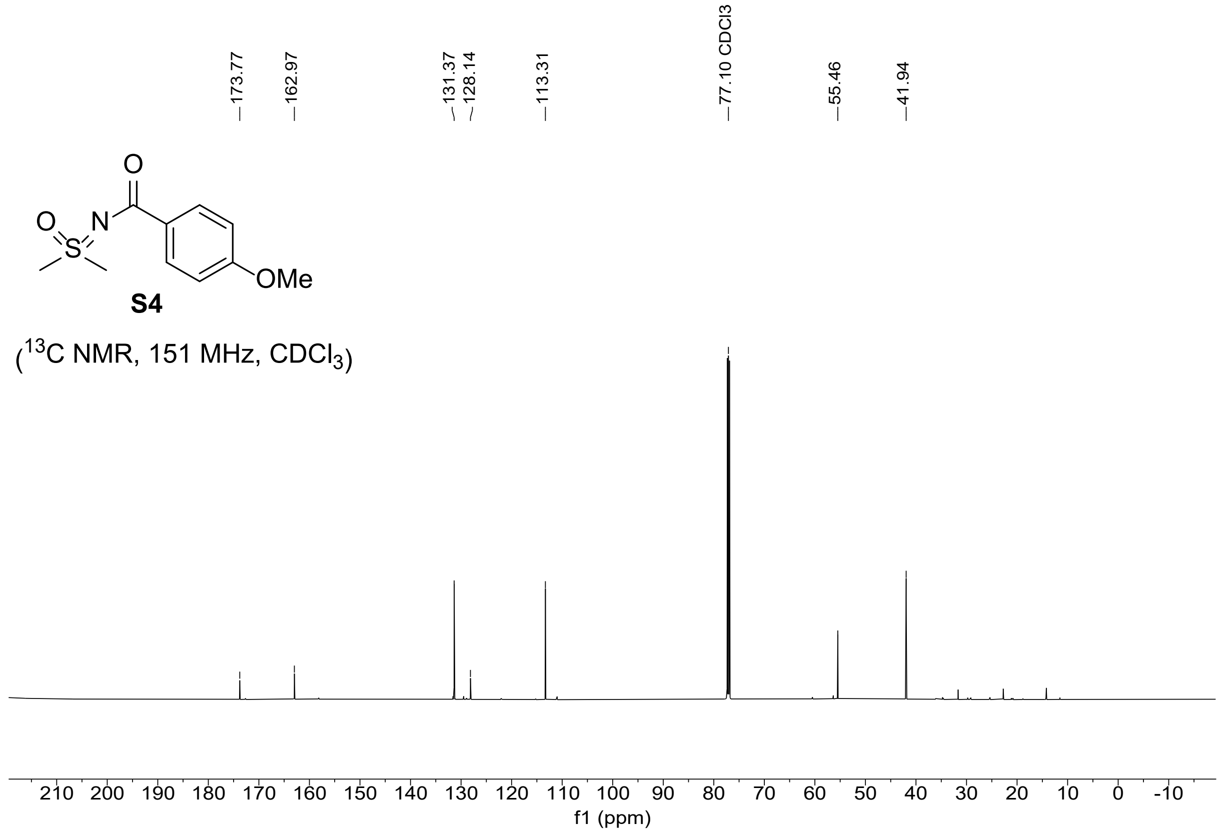
173.77
162.97
131.37
128.14
113.31
77.10 CDCl3
55.46
41.94
O
N
O
S
OMe
S4
(13C NMR, 151 MHz, CDCl3)
210 200 190 180 170 160 150 140 130 120 110 100 90 80 70 60 50 40 30 20 10 0 -10
f1 (ppm)

References

(1) Suzuki, Y.; Iinuma, M.; Moriyama, K.; Togo, H. TEMPO-Mediated Oxidation of Alcohols with Ion-Supported (Diacetoxyiodo)Benzenes. *Synlett* **2012**, *23* (8), 1250–1256. https://doi.org/10.1055/s-0031-1290766.

(2) Doscher, M. S. [48] Solid-Phase Peptide Synthesis. In *Methods in Enzymology*; Enzyme Structure Part E; Academic Press, **1977**; Vol. 47, pp 578–617. https://doi.org/10.1016/0076-6879(77)47050-2.

(3) Pang, X.; Dong, J.; Gao, L.; Wang, L.; Yu, S.; Kong, J.; Li, L. Dansyl-Peptide Dual-Functional Fluorescent Chemosensor for Hg^{2+} and Biothiols. *Dyes Pigm.* **2020**, *173* (1), 107888. https://doi.org/10.1016/j.dyepig.2019.107888.

(4) Friis, S. D.; Lindhardt, A. T.; Skrydstrup, T. The Development and Application of Two-Chamber Reactors and Carbon Monoxide Precursors for Safe Carbonylation Reactions. *Acc. Chem. Res.* **2016**, *49* (4), 594–605. https://doi.org/10.1021/acs.accounts.5b00471.

(5) Qi, X.; Jiang, L.-B.; Li, H.-P.; Wu, X.-F. A Convenient Palladium-Catalyzed Carbonylative Suzuki Coupling of Aryl Halides with Formic Acid as the Carbon Monoxide Source. *Chem. - Eur. J.* **2015**, *21* (49), 17650–17656. https://doi.org/10.1002/chem.201502943.

(6) Miller, M. K.; Wang, H.; Hanaya, K.; Zhang, O.; Berlaga, A.; Ball, Z. T. Copper-Mediated Peptide Arylation Selective for the N-Terminus. *Chem. Sci.* **2020**, *11* (38), 10501–10505. https://doi.org/10.1039/D0SC02933E.

(7) Correa, A.; Bolm, C. Iron-Catalyzed C-N Cross-Coupling of Sulfoximines with Aryl Iodides. *Adv. Synth. Catal.* **2008**, *350* (3), 391–394. https://doi.org/10.1002/adsc.200700508.

(8) Vaddula, B.; Leazer, J.; Varma, R. S. Copper-Catalyzed Ultrasound-Expedited N-Arylation of Sulfoximines Using Diaryliodonium Salts. *Adv. Synth. Catal.* **2012**, *354* (6), 986–990. https://doi.org/10.1002/adsc.201100808.

(9) Moessner, C.; Bolm, C. $Cu(OAc)_2$-Catalyzed N-Arylations of Sulfoximines with Aryl Boronic Acids. *Org. Lett.* **2005**, *7* (13), 2667–2669. https://doi.org/10.1021/ol050816a.

(10) Hande, S.; Mfuh, A.; Throner, S.; Wu, Y.; Ye, Q.; Zheng, X. Photoredox Mediated C-N Cross Coupling of Sulfoximines with Aryl Iodides. *Tetrahedron Lett.* **2019**, *60* (41), 151100. https://doi.org/10.1016/j.tetlet.2019.151100.

(11) Han, S. H.; Lee, K.; Noh, H. C.; Lee, P. H. Palladium(II)-Catalyzed *N* -Carbonylative Cross-Coupling Reaction of Sulfoximines with Aryl, Heteroaryl, and Alkenyl Halides Using Tungsten Hexacarbonyl as Carbon Monoxide Source. *Asian J. Org. Chem.* **2021**, *10* (9), 2397–2405. https://doi.org/10.1002/ajoc.202100388.
